# Silver(I) Ions Bridging Oxadiazole-2-thione Derivatives with Mitochondriotropic Agents: A Promising Strategy for Potent Anticancer Chemotherapy

**DOI:** 10.1021/acs.inorgchem.5c01095

**Published:** 2025-06-24

**Authors:** Christina N. Banti, George Vagenas, Catherine P. Raptopoulou, Vassilis Psycharis, John C. Plakatouras, Sotiris K. Hadjikakou

**Affiliations:** † Biological Inorganic Chemistry laboratory, Department of Chemistry, 37796 University of Ioannina, Ioannina 45110, Greece; ‡ Institute of Nanoscience and Nanotechnology, NCSR “Demokritos”, Athens 15310, Greece; § Institute of Materials Science and Computing, University Research Center of Ioannina (URCI), Ioannina 45110, Greece

## Abstract

The conjugation of phenyl-substituted 1,3,4-oxadiazole-2-thione derivatives (X-PODTH, where X = H (PODTH) (**1a**), o-F (o-F-PODTH) (**2a**), p-F (p-F-PODTH) (**3a**), o-Cl (o-Cl-PODTH) (**4a**), and m-Cl (m-Cl-PODTH) (**5a**)) with triphenylphosphine (TPP) via silver­(I) ions results in the formation of heteroleptic compounds [Ag­(TPP)_3_(X-PODTH)] (X = H (**1**), o-F (**2**), p-F (**3**), o-Cl (**4**), and m-Cl (**5**)). These conjugates were thoroughly characterized using various techniques, including melting point (mp), X-ray fluorescence (XRF), attenuated total reflection Fourier-transform infrared (ATR-FTIR), ultraviolet–visible (UV–vis), and nuclear magnetic resonance (^1^H, ^31^P NMR) spectroscopies. The crystal structures of **1a**–**5a**, and their complexes (**1**–**5**) were determined through single-crystal X-ray diffraction (XRD) and powder X-ray diffraction (PXRD) analyses. Compounds **1**–**5** demonstrated significant inhibition of human breast adenocarcinoma cell proliferation in both hormone-dependent (HD, MCF-7) and hormone-independent (HI, MDA-MB-231) cell lines. Their nongenotoxicity was confirmed *in vitro* using the micronucleus (MN) assay. The mechanism of action was further explored through studies of cell morphology, acridine orange ethidium bromide (AO/EB) staining, cell cycle arrest analysis, mitochondrial membrane permeabilization (MMP) assays, DNA binding affinity studies, and lipoxygenase (LOX) inhibitory activity. These findings were supported and rationalized through regression analysis, providing valuable insights into their structural biological activity.

## Graphical abstract

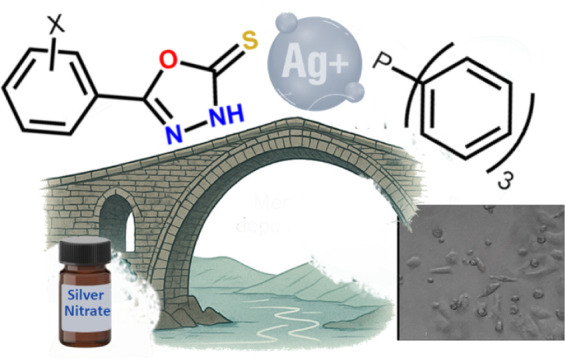

## Introduction

Oxadiazole-2-thione derivatives have emerged as a significant class of heterocyclic compounds in medicinal chemistry, known for their diverse pharmacological activities.^1,2^ These compounds exhibit a range of biological properties, including anticancer,^3–5^ antimicrobial^6^ and anti-inflammatory^7–9^ activities, making them valuable candidates in drug discovery and development. Moreover, several drugs featuring a 1,3,4-oxadiazole core are currently used in various therapeutic areas, such as zibotentan which is used for cancer treatment,^10^ furamizole is employed as antibiotic,^11^ raltegravir serves as an antiviral drug for HIV infection,^12,13^ etc. There are few studies on the anticancer activity of oxadiazoles and their derivatives, against various cancer cell types^14^ and even less for their metal complexes.^15–21^ Additionally, their anticancer properties are of particular interest, as these derivatives have been shown to induce apoptosis and inhibit the proliferation of cancer cells by interacting with key cellular pathways.^22^

Triaryl derivatives of pnictogens (tpE; E= P, As, or Sb) exhibit significant biological activity against various cell types.^23,24^ These lipophilic, mitochondriotropic compounds disrupt mitochondrial membrane permeabilization, triggering the intrinsic apoptotic pathway and leading to programmed cell death.^25^ Phosphorus-containing molecules have shown promising anticancer activity by specifically targeting mitochondria.^26–30^

Therefore, there is great interest in investigating whether the combination of an oxadiazole-2-thone derivative with a triphenyl derivative phosphorus could lead to a better antiproliferative formulation. Silver­(I) ions were chosen as the linker in this case due to their low toxicity to humans and their previously established antitumor activity.^26–30^ Moreover, silver­(I) metallodrugs demonstrate significant *in vitro* antiproliferative activity against various cancer cell lines, while their effectiveness surpasses that of cisplatin.^30^ The antiproliferative effects of silver­(I) ions are primarily due to their ability to bind to DNA bases, interact with protein thiol groups, and disrupt mitochondrial function, which thereby triggering the mitochondrial apoptotic pathway.

In the course of our studies in the field of drug design and development,^26–33^ a series of new efficient metallodrugs for the breast cancer chemotherapy by the combination of phenyl-substituted 1,3,4-oxadiazole-2-thione derivatives (X-PODTH, where X = H (PODTH) (**1a**), o-F (o-F-PODTH) (**2a**), p-F (p-F-PODTH) (**3a**), o-Cl (o-Cl-PODTH) (**4a**), and m-Cl (m-Cl-PODTH) (**5a**)) (Scheme 1) with TPP through silver­(I) ions were prepared. These new compounds of formula [Ag­(TPP)_3_(X-PODTH)] (X = H (**1**), o-F (**2**), p-F (**3**), o-Cl (**4**), and m-Cl (**5**)), were characterized by mp, XRF (X-ray fluorescence spectroscopy), ATR-FTIR, UV–vis, and ^1^H, ^31^P NMR spectroscopies. The crystal structures of **1a**–**5a**, and their complexes **1**–**5** were determined by XRD and PXRD analyses. The *in vitro* cytotoxic activity of **1**–**5** and their ligands was evaluated against MCF-7 (HD) and MDA-MB-231 (HI) and correlate with cisplatin. Their molecular mechanism of action and the nature of their interaction with intracellular components (DNA and LOX) is also investigated. The structure activity relationship is investigated by employing substituents (X) at o-, m-, p-positions on the phenyl ring, causing different electronic effects, which consequently influence the charge distribution on the sulfur atom. This regulates the geometry of the compounds and therefore their biological activity. The tautomerism between thiol–thione forms undergo in solution ensure the structural diversity of this type of compound.

**Scheme 1:**
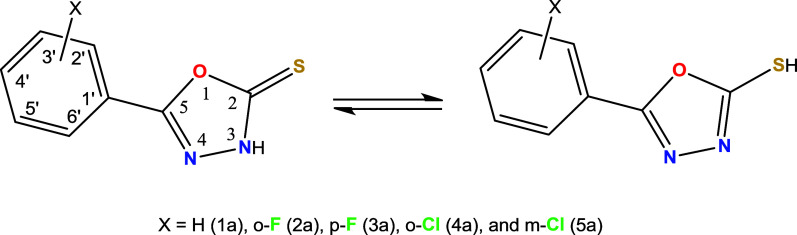
(*o*-,*m*-,*p*-)­X-2-Phenyl-1,3,4-oxadiazole-2-thione and Its Tautomeric Form

## Results and Discussion

### General Aspects

The five ligands **1a**–**5a** used in this study were previously synthesized as described in refs 34 and 35 New samples were prepared following these reported procedures (Scheme 2). Among these compounds, only **1a** had been previously characterized by single-crystal X-ray diffraction. The **2a**–**5a** had not been structurally characterized by XRD until now.

**Scheme 2:**
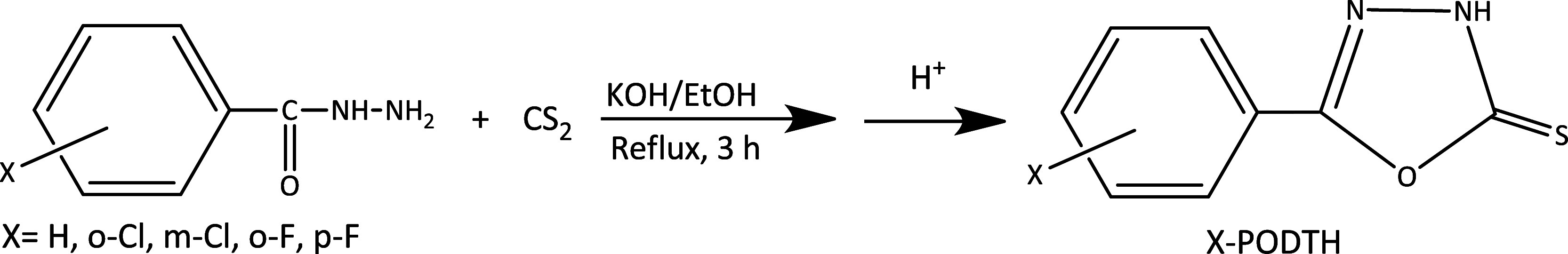
Synthetic Route of the Ligands R-PODTH

Aqueous solution of silver nitrate reacts with the ligand X-PODTH which has been previously treated with an equimolar amount of KOH to form the [Ag­(L)]*_n_* intermediate (Scheme 3). The intermediate reacts with the mitochondriotropic pnictogen derivative of TPP in DMSO in 1:3 molar ratio. The resulting crystalline product was collected after a few days filtered off and dried over silica. The formula of compounds was initially determined using spectroscopic methods. The crystal and molecular structures of the conjugates were solved and refined through XRD analysis.

**Scheme 3:**
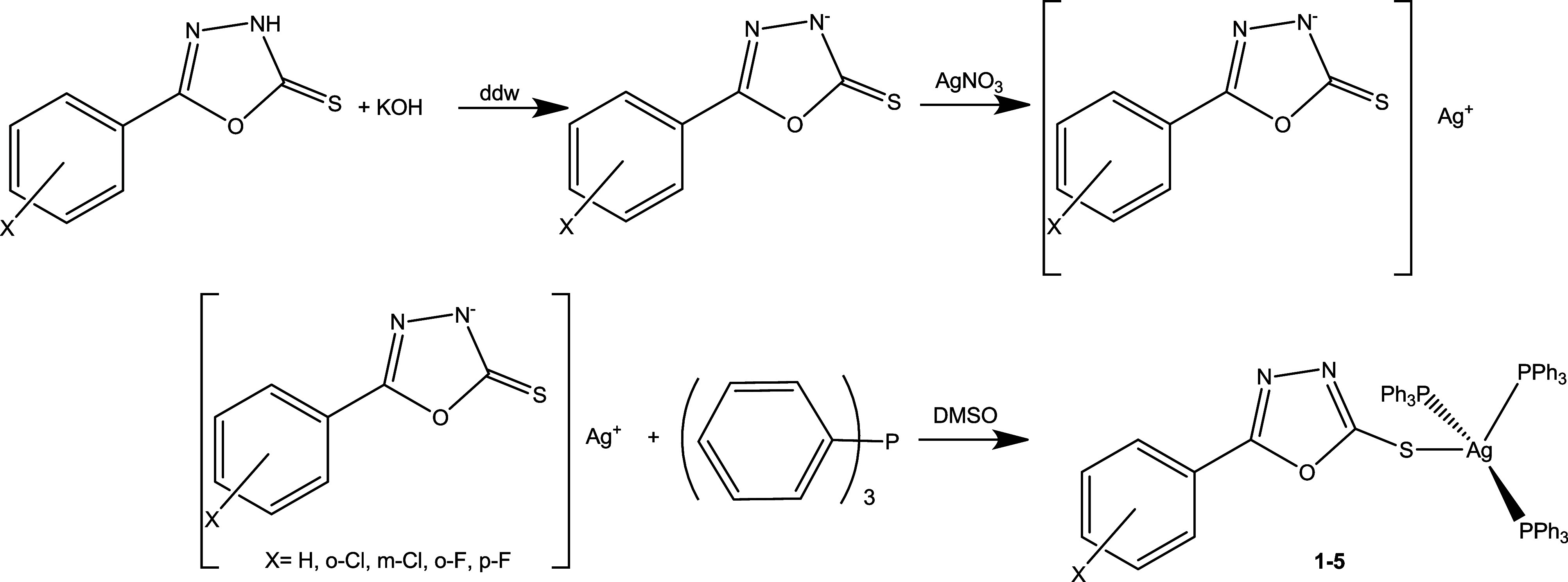
Synthesis of the Complexes **1**–**5** (ddw= Double Distilled Water)

### Solid State Studies

#### Crystal and Molecular Structures of the Ligands **1a**–**5a**

Molecular diagrams along with their selected bond lengths and angles of **1a**–**5a** are shown in Figure 1 and Table S1 respectively.

**Figure 1:**
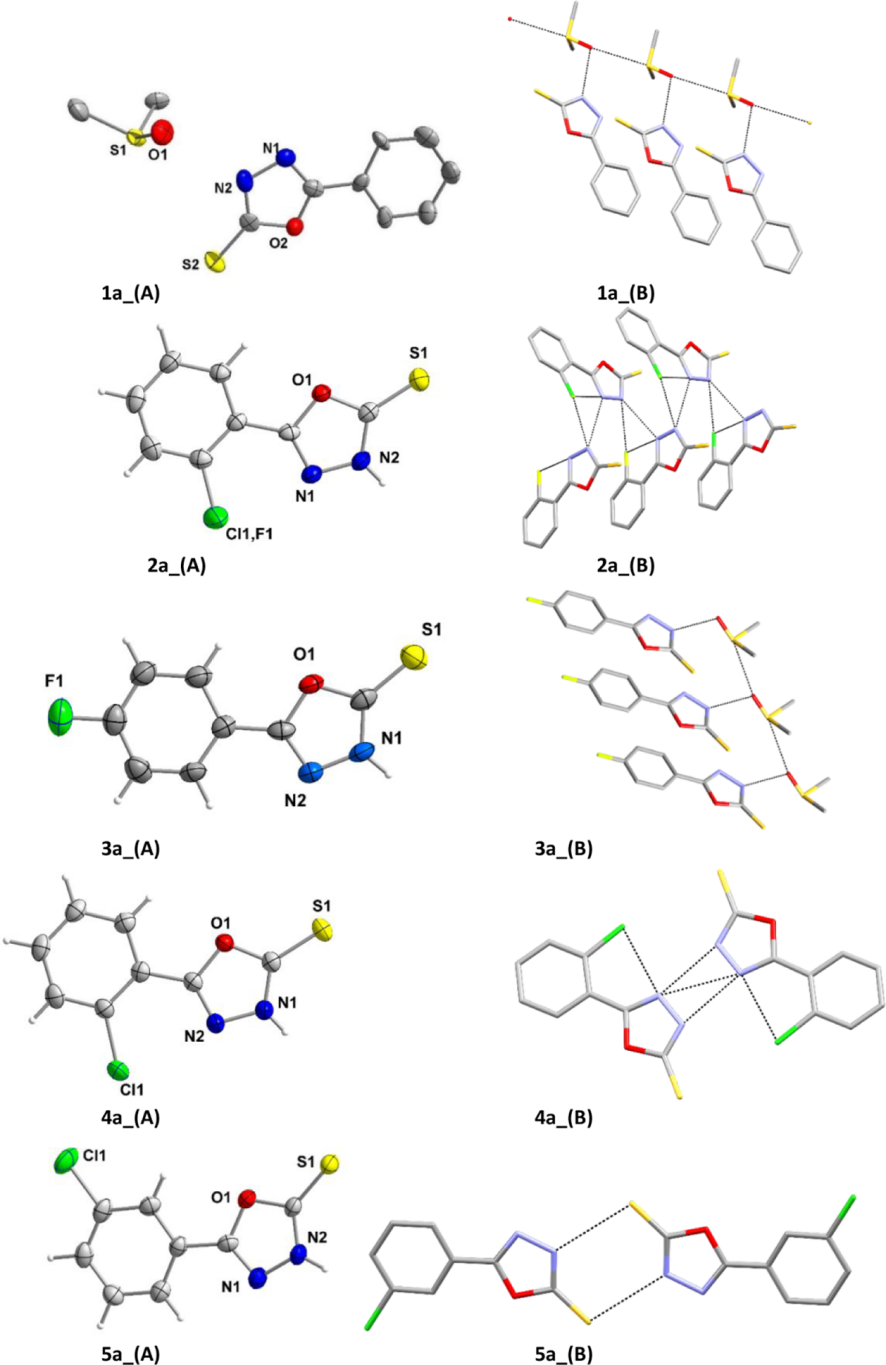
Ortep type plots of **1a**–**5a** with thermal ellipsoids shown at 50% probability. (B) Strong intermolecular interactions leading to supramolecular assembly **1a–5a**.

The crystal and molecular structure of **1a** is already reported elsewhere.^36^ Compound **1a** displays multiple polymorphic forms. The unit cell parameters of 1a determined within this work are space group *P*2_1_2_1_2_1_, *a* = 4.7615(8) Å, *b* = 9.8297(19) Å, *c* = 26.787(6) Å, *V* = 1253.74 Å^3^, *Z* = 4, while in the previously published structures are as follows: space group: *P*2_1_/*n*; unit cell dimensions: *a* = 11.341(1) Å, *b* = 4.659(1) Å, *c* = 16.024(0) Å, β = 103.84(0)°^36^ space group: *Pbca* Cell: *a* = 10.017(2), *b* = 8.668(1); *c* = 17.634(4) Å^36^ and space group: *P*2_1_/*c* Cell: *a* = 5.577(0), *b* = 19.988(1), *c* = 7.228(0), *b* = 100.46(0)°.^36^ Therefore, **1a** is a polymorphic form of those reported previously. The diversity of polymorphs prompted us to collect X-ray diffraction data and to proceed with structure solution and refinement of **1a**. The rest of the ligands **2a**–**5a** had not been structurally characterized by XRD until now. In this study, their crystal and molecular structures, as well as those of their corresponding silver­(I) complexes, are reported for the first time. Despite numerous attempts to grow single crystals of **1a** suitable for detailed structural analysis, the data quality remained low. Using the available data, we were able to generate a preliminary structural model (Figure 1A). To further verify the structure of **1a**, its powder diffraction pattern was analyzed (Figure S1). The PXRD pattern (red line) recorded from compound **1a**, compared to the simulated pattern of the compound which was calculated using the structural model provided in the CIF file with CCDC #636720, confirms the formation of phenyl-oxadiazole-2-thione.^36^

The structures of phenyl derivatives of oxadiazol-2-thiones **1a**–**5a** show typical bond lengths and angles for the core oxadiazole-thione moiety. Moreover, the refinement of the crystal structure of the ligands **1a**–**5a** shows the prevalence of the thione form (−NH–C=S). This is confirmed by the presence of the H atom on the N atom of the oxadiazol ring and the S–C bond distance, which ranges from 1.638(3) Å to 1.648(5) Å (Table S1), close to the corresponding one found by Chaves et.al (1.643(2) Å).^37^ The S–C bond distance remains consistent across compounds, with minor variations likely due to structural constraints (Table S1). The double bond character of the C=S bond and the planar configuration of the >C=S moiety confirms the sp^2^ hybridizm of C atom. The N–C­(=S) bonds in the oxadiazole ring (around 1.325–1.333 Å) are shorter from the corresponding average single N–C bond (1.47 Å) suggesting partial double bond, which is expected due to the phenyl substituent (Table S1). A slight increase in C–N bond length from **1a** to **5a**, suggesting influence from F- or Cl-substituents in o-, m- or p-positions of the phenyl ring on the nitrogen center across different compounds. The N–H bond length varied between 1.11 and 0.83 Å (1.11 (**1a**), 0.84(3) (**2a**), 0.88(6) (**3a**), 0.83(3) (**4a**) and 0.86(5) (**5a**) Å) (Table S1). Moreover, the supramolecular assemblies are stabilized by hydrogen bonds (Figure 1B).

Hydrogen bonding interactions such as H­[N]···O or H­[N]···S, play a significant role in crystal packing. The H­[N]···X hydrogen bonds show typical bond distances of around 2.9–3.2 Å, with bond angles close to linearity (N–H···X angle ranging from 160° to 180°), indicating strong directional hydrogen bonds.

#### Crystal and Molecular Structures of the Complexes **1**–**5**

Molecular diagrams along with their selected bond lengths and angles of **1**–**5** are shown in Figure 2 and Table S2 respectively.

**Figure 2:**
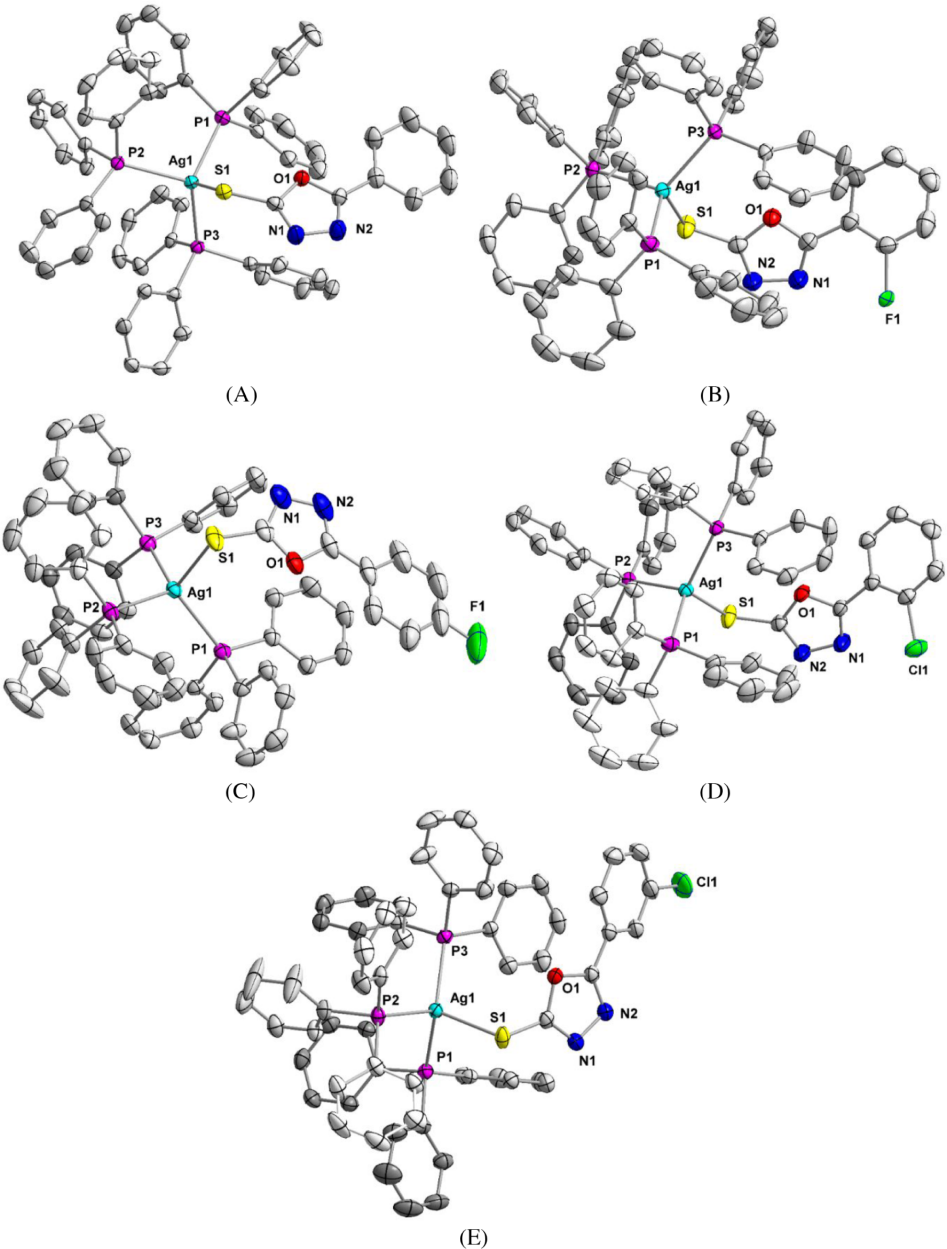
Ortep type plots of [Ag­(TPP)_3_(X-PODTH)] (**A:** X = H (**1**), **B:** o-F (**2**), **C:** p-F (**3**), **D:** o-Cl (**4**), and **E:** m-Cl (**5**)) with thermal ellipsoids shown at 50% probability. Hydrogen atoms are not shown for clarity reasons.

In each compound, the silver ion is coordinated by one sulfur atom from oxadiazole-2-thione ligand and three phosphorus atoms from phosphines, forming a tetrahedral geometry around the metal center. The oxadiazole-2-thione is monodentate coordinated to silver­(I) through S-atom. The range of the >C=S bond (approximately 1.69–1.71 Å) indicates subtle differences in how the sulfur ligand interacts with the silver center.^38^ The slightly shorter Ag–S bond in compounds **3** and **5** suggests a tighter coordination or reduced steric hindrance around the silver–sulfur interaction compared to the others **1, 2** and **4**. Three different Ag–P bonds (Ag1–P1, Ag1–P2, Ag1–P3) in each compound also show slight variations.

The Ag1–P1 bond length in **5** is slightly longer (2.6111(6) Å) than in other compounds, indicating a potential weakening of this interaction due to electronic or steric effects unique to compound **5**.

The Ag1–P3 bond length in compound **3** is notably longer (2.5863(8) Å), possibly again due to steric or electronic influences that weaken the interaction at this specific site. These variations in Ag–P distances suggest a degree of asymmetry in the silver-ligand coordination, with subtle shifts in bonding strength depending on the ligand environment.

The S–Ag–P angles show significant variability, particularly in compounds **2** and **4**, which display larger angles than the others. The P–Ag–P angles further highlight structural diversity.

Each compound demonstrates unique structural characteristics, with differences in bond distances and angles reflecting slight but meaningful shifts in ligand orientations and coordination geometry around silver. These variations likely result from different steric and electronic environments created by the ligands, leading to distinct conformational and packing behaviors in the crystal structures. Figure 3 presents the core parts of all complexes in overlay mode. Table S3 lists the Ag–S–C angles and the smallest dihedral angles with respect to one phosphorus atom coordinated to Ag cation.

**Figure 3:**
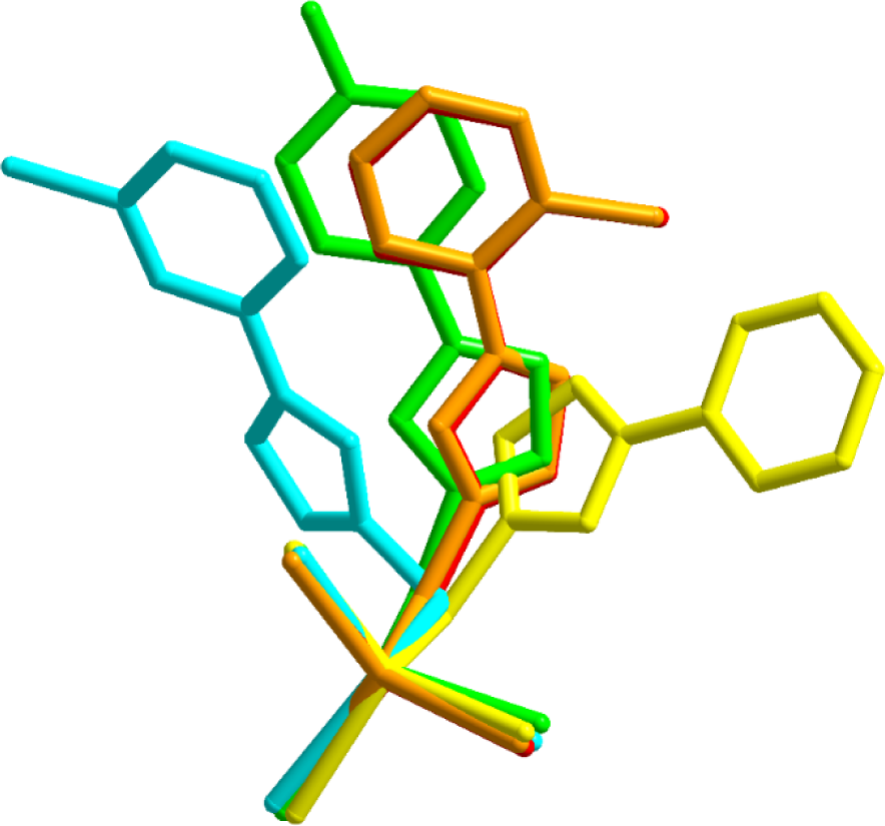
Overlay presentation of the core part of complexes. Yellow, orange, light green, red and light blue colors have been used to present **1**–**5** respectively. Complexes **2** (orange) and **4** (red) have almost identical orientations.

#### Vibrational Spectroscopy

The v­(N–H) stretching band appears at 3225–3276 cm^–1^ in X-PODTH ligands and is absent in complexes **1**–**5**, confirming the deprotonation of thioamide group^34,35^ (Figures S2–S6).

The thioamide I band (δ­(N–H) + ν­(C–N)) occurs at 1499–1506 cm^–1^ in X-PODTH and shifts to 1483–1510 cm^–1^ in complexes **1**–**5**, indicating coordination through the thioamide group. The thioamide II band (ν­(C–N) + δ­(N–H) + ν­(C=S)), observed at 1276–1284 cm^–1^ in ligands, vanishes in **1**–**5**, suggesting deprotonation and S-coordination via the >C=S group.^34,35,39^ The thioamide III band (ν­(C–N) + ν­(C–S)) shifts from 962–968 cm^–1^ in X-PODTH to 949–966 cm^–1^ in **1**–**5**, supporting sulfur coordination. The thioamide IV band (ν­(C–S)) appears at 685–692 cm^–1^ in ligands and shifts to 673–690 cm^–1^ in **1**–**5**, further confirming S-bonding^34,35,39^ (Figures S2–S6).

The C–O–C stretching band shifts from 1069–1080 cm^–1^ in X-PODTH to 1062–1092 cm^–1^ in **1**–**5**, indicating slight perturbation upon complexation^34,35^ (Figures S2–S6).

Finally, the ν_sym_(C–P) bands of free TPP at 511 and 488 cm^–1^ shift to 502–471 cm^–1^ in **1**–**5**, confirming coordination to the metal. Although the presence alone of these bands does not confirm coordination of TPP; the shifts relative to free TPP do^34,35^ (Figures S2–S6). Additional discussion is provided in the Supporting Information.

### Solution Studies

#### ^1^H NMR Spectroscopy

The signals at 7.41–7.39 ppm (m) in the ^1^H NMR spectrum of TPP are attributed to the *m*- and *p*-H­[Ph-], while those at 7.27–7.20 ppm (m) correspond to the *o*-H­[Ph-].

Moreover, the resonance signals in the ^1^H NMR spectra of the oxadiazole-2-thone ligands appears in the range of 7.98–7.84 ppm for H^2′,6′^ or H^6′^ protons (H-PODTH: 7.90–7.88 ppm, *o*-F-PODTH: 7.93–7.89 ppm, *p*-F-PODTH: 7.98–7.93 ppm, *o*-Cl-PODTH: 7.93–7.91 ppm, *m*-Cl-PODTH: 7.87–7.84 ppm) and in the range of 7.73–7.41 ppm for the H^3′,4′,5′^ or H^3′,5′^/H^4′,5′^ protons (H-PODTH: 7.67–7.58 ppm, *o*-F-PODTH: 7.72–7.41 ppm, *p*-F-PODTH: 7.46–7.42 ppm, *o*-Cl-PODTH: 7.72–7.55 ppm, *m*-Cl-PODTH: 7.73–7.61 ppm) (Figures S7–S11).^40^

The resonance signals in the ^1^H NMR spectra of the complexes are observed at 7.47–7.39 ppm (**1**), 7.51–7.40 ppm (**3**), 7.47–7.36 ppm (**5**) for the H^2′,6′^ (Ph-substituted ring), while the H^3′,4′,5′^ or H^4′,5′^ and TPP protons (*p*-, *m*-, o-H) are observed at 7.33–7.24 ppm (**1**), 7.35–7.25 ppm (**3**), 7.33–7.24 ppm (**5**). Compound **2** shows signals for H^6′^[*o*-F-Ph] at 7.65–7.39 ppm and at 7.33–7.23 ppm for H^3′,4′,5′^[*o*-F-Ph] and TPP protons.

The signals in the ^1^H NMR spectrum of compound **4** at 7.57–7.42 ppm is assigned to H^6′^[*o*-Cl-Ph], while those at 7.39–7.32 ppm are due to the H^3′,4′,5′^[*o*-Cl-Ph] and TPP protons. These downfield or upfield shifts compared to the free ligands support complex formation in solution (Figures S7–S11).^40^

Additional discussion is provided in the Supporting Information page S2.

#### ^31^P NMR Spectroscopy

The ^31^P NMR spectra of **1**–**5** in DMSO-*d*_6_ solution, are shown Figure 4. The ^31^P NMR spectrum of TPP displays a characteristic resonance signal at – 6.849 ppm, while the corresponding one of triphenylphosphine oxide (TPP=O) exhibits a resonance signal at 25.500 ppm (Figure 4). The chemical shifts in ^31^P NMR spectra of **1**–**5** show shifted to distinct for each compound: 2.833 (**1**), 2.522 (**2**), 2.335 (**3**), 2.601 (**4**), and 4.108 (**5**) ppm respectively (Figure 4). These data confirm the coordination of TPP to silver ions through phosphorus in **1**–**5**. Although low-temperature ^31^P NMR measurements could potentially reveal the coupling of phosphorus with silver isotopes (^10^_7_Ag and ^109^Ag) by resolving the broad singlet into distinct doublets, such experiments were not feasible in our study. The 250 MHz ^1^H NMR spectrometer used does not support variable-temperature measurements, and the chosen solvent, DMSO-*d*_6_, where **1**–**5** were solubilized, has a freezing point at 19 °C, restricting low-temperature operation.

**Figure 4:**
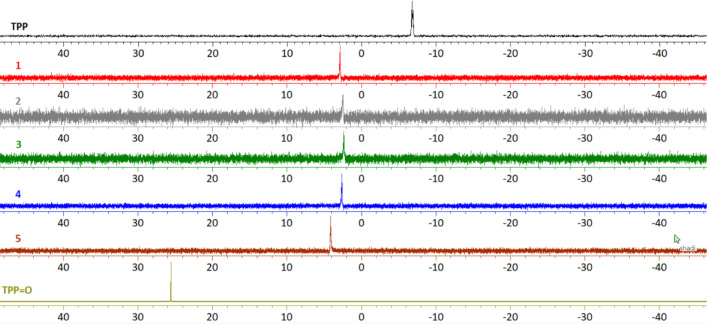
^31^P NMR spectra of **1**–**5** in DMSO-*d*_6_ solution.

### Stability Studies

The retention of the molecular structures of compounds **1**–**5** in DMSO was confirmed using UV–vis and ^1^H NMR spectroscopy over a 48-h period, which corresponds to the incubation period required for the biological experiments (Figures S12–S26).

No changes were observed between the initial UV–vis or ^1^H NMR spectra and those obtained after 48 h. The stability of **1**–**5** was also examined under physiological conditions in PBS buffer solution at 37 °C using UV–vis spectroscopy. Throughout the observation period, no changes were detected between the initial spectra and those recorded after 48 h, confirming the stability of the compounds under physiological conditions (Figures S17–S21). Consequently, no release of the ligands from the complexes is observed at any stage.

### In Vitro Antiproliferative Activity

The antiproliferative properties of compounds **1**–**5**, as well as their ligands, were evaluated against human breast adenocarcinoma cell lines, MCF-7 and MDA-MB-231, using the sulforhodamine B (SRB) assay over a 48-h incubation period (Table 1).

**Table 1:** IC_50_ Values of **1**–**5** and Their Ligands against Human Breast Adenocarcinoma Cell Lines (MCF-7, MDA-MB-231), Normal Human Fetal Lung Fibroblast Cell Line (MRC-5) and Non-Tumorigenic Human Breast Epithelial Cell Line (MCF-10A)

	IC_50_ values (μΜ)^a^								
complex	MCF-7	MDA-MB 231	MRC-5	MCF-10A	TPI^b^ ^,^ ^c^	TPI^d^	TPI^e^	TPI^f^	ref
**1**	2.03 ± 0.09	4.09 ± 0.17	3.77 ± 0.16	10.57 ± 0.31	1.9	0.9	5.2	2.6	this study
**2**	2.50 ± 0.19	3.90 ± 0.14	3.12 ± 0.11	13.91 ± 0.73	1.2	0.8	5.6	3.6	this study
**3**	2.15 ± 0.16	2.52 ± 0.07	2.72 ± 0.19	10.87 ± 0.64	1.3	1.1	5.1	4.3	this study
**4**	3.00 ± 0.15	4.27 ± 0.15	4.07 ± 0.24	16.04 ± 0.50	1.4	1.0	5.3	3.7	this study
**5**	1.69 ± 0.08	3.55 ± 0.19	2.89 ± 0.08	16.13 ± 0.51	1.7	0.8	9.5	4.5	this study
**1a**	>30	>30	>30		–	–	–	–	this study
**2a**	>30	>30	>30		–	–	–	–	this study
**3a**	>30	>30	>30		–	–	–	–	this study
**4a**	>30	>30	>30		–	–	–	–	this study
**5a**	>30	>30	>30		–	–	–	–	this study
TPP	28.9 ± 1.4	>30	>30		–	–	–	–	^28^
cisplatin	5.5 ± 0.4	26.7 ± 1.1	1.1 ± 0.2	11.0 ± 3.0	0.2	0.04	2.0	0.4	^28^,^42^

a The cells’ incubation period was maintained at 48 h in all experiments.

b TPI (Therapeutic Potency Index) is defined as the ratio of the IC_50_ value against normal cell line (same tissue (MCF-10A) or similar tissue (MRC-5)) to the corresponding IC_50_ value against cancer cell lines.

c TPI = {IC_50_^MRC‑5^/IC_50_^MCF‑7^}.

d TPI = {IC_50_^MRC‑5^/IC_50_^MDA‑MB‑231^}.

e TPI = {IC_50_^MCF‑10A^/IC_50_^MCF‑7^}.

f TPI = {IC_50_^MCF‑10A^/IC_50_^MDA‑MB‑231^}.

Compounds **1**–**5** exhibit up to 3.3- and 10.6-fold greater activity compared to cisplatin against MCF-7 and MDA-MB-231 cells, respectively (Table 1).

Moreover, **1**–**5** exhibit selectivity toward hormone receptor-positive breast cancer cells, with IC_50_ values from 1.7 to 3.0 μM, compared to hormone receptor-negative cells, where IC_50_ values lie between 2.5 and 4.3 μM. Against MCF-7 cells, compound **5** shows the most potent cytotoxic activity, with an IC_50_ of 1.69 ± 0.08 μM.

Against MDA-MB-231 cells, compound **3** demonstrates the highest activity, with IC_50_ = 2.52 ± 0.07 μM. The ligands showed no activity in either cell line at concentrations up to 30 μM.

Complexes **3** and **5**, which contain the ligands p-F-PODTH and m-Cl-PODTH, exhibit IC_50_ values between 1.7 to 3.6 μM. In contrast, the presence of halogen substituents in the ortho position (o-X-PODTH, where X = F (**2**) or Cl (**4**)) results in lower activity, with IC_50_ values between 2.5 and 4.3 μM, against both cancer cell lines.

In order to verify whether the biological activity is due to the entire complex itself or to its components, MCF-7 cells were treated as mixtures of 1,3,4-oxadiazole-2-thiones derivatives/TPP in 1:1 molar ratio. The IC_50_ values of the mixtures 1,3,4-oxadiazole-2-thione derivatives/TPP (1:1) are 28.0 ± 0.8 (**1a**/TPP), 29.7 ± 0.9 (**2a**/TPP), 28.3 ± 1.1 (**3a**/TPP), 31.8 ± 1.1 (**4a**/TPP) and 32.6 ± 0.7 (**5a**/TPP) μΜ. The observed antiproliferative effect of mixtures against MCF-7 cells is close to the corresponding one of TPP alone (IC_50_ = 28.9 ± 1.4 μM). Moreover, ligands 1,3,4-oxadiazole-2-thiones derivatives **1a**–**5a** show IC_50_ higher than 30 μM, with (%) cells viability 84.7 (**1a**), 95.7 (**2a**), 90.7 (**3a**), 80.3 (**4a**) and 90.1 (**5a**) %. This confirms that the antiproliferative activity in the case of the complexes is attributed to the entire complex rather than their components.

The toxicity of **1**–**5** was also evaluated against normal human fetal lung fibroblast (MRC-5) cells. Their IC_50_ values lie from 2.7 to 4.1 μM (Table 1). The toxicity of these compounds was also evaluated against the nontumorigenic human breast epithelial cell line (MCF-10A), which is widely used as a model for normal human breast cells. They closely resemble normal human mammary epithelial cells in structure and are regulated by hormones and growth factors. However, they do not express estrogen receptors and show no signs of terminal differentiation.^41^ The IC_50_ values of **1**–**5** are in the range of 10.6 to 16.1 μΜ (10.6 (**1**), 13.9 (**2**), 10.9 (**3**), 16.0 (**4**) and 16.1 μΜ (**5**)). For comparison the IC_50_ values of cisplatin against MCF-10A is IC_50_= 11.0 ± 3.0 μM.^42^

The Therapeutic Potency Index (TPI) is defined as the ratio of the IC_50_ value against normal cell lines to the IC_50_ value against cancer cells of the same or a similar tissue origin. Compounds **1**–**5** exhibit notable antitumor activity against MCF-7 cells, with comparatively lower toxicity toward MRC-5 cells (Table 1). Their TPI values range from 1.2 to 1.9, indicating a favorable therapeutic window

In contrast, cisplatin shows significantly lower TPI values, 0.2 against MCF-7 cells relative to MRC-5, and as low as 0.04 against MDA-MB-231 cells.^26–31^

Moreover, the corresponding TPI values for compounds **1**–**5** in respect to MCF-10A cells range from 5.1 to 9.5 against MCF-7 cells and 2.6 to 4.5 against MDA-MB-231 cells (Table 1). In comparison, the TPI values of cisplatin against MCF-7 and MDA-MB-231 cells, relative to MCF-10A, range between 2.0 and 0.4.^42^

According to the guidelines of the U.S. Food and Drug Administration (FDA), a compound is considered ″selective″ if the ratio of its Minimum Toxic Concentration (MTC or IC_50_ against normal cells) to Minimum Effective Concentration (MEC or IC_50_ against cancer cells) exceeds 2.^43^ Based on this criterion, compounds **1**–**5** demonstrate enhanced antitumor activity relative to their toxicity, particularly against MCF-7 cells (Table 1).

### In Vitro Micronucleus Assay

The *in vitro* MN assay is employed to evaluate the potential of various agents to induce DNA damage, as the presence of MN serves as a biomarker for mutagenic, genotoxic, or teratogenic effects.^44^ The MN assay is also utilized in pharmaceuticals development.^26–31,45^ MN are either chromosome fragments or entire chromosomes.^46,47^

The relative ratio of MN in MRC-5 cell culture after incubation with complexes **1**–**5**, in respect to the corresponding one of the untreated cells, are as follows: 1.38 (**1**), 1.24 (**2**), 1.31 (**3**), 1.69 (**4**), and 2.34 (**5**) %, respectively. In comparison, the relative ratio of MN in MRC-5 cells culture treated with cisplatin, toward its control, is 2.00 (Table 2 and Figure 5).^28^ While the percentage of MN in MRC-5 cells treated with **1**–**5** increases compared to untreated cells, these values remain lower or comparable to those observed with cisplatin, a well-known anticancer drug used in clinical settings (Table 2 and Figure 5). Compound **2** shows the lowest genotoxicity, indicating no significant mutagenic, genotoxic, or teratogenic effects.

**Table 2:** Percentage (%) and Relative Ratio of Micronucleus (MN) after Incubation of **1**–**5** and Cisplatin toward MRC-5 Cells

	Percentage of MN^a^ (%)	Relative ratio	refs
control	2.9 ± 0.2	1.00	this study
**1**	4.0 ± 0.6	1.38	this study
**2**	3.6 ± 0.5	1.24	this study
**3**	3.8 ± 0.2	1.31	this study
**4**	4.9 ± 0.5	1.69	this study
**5**	6.8 ± 1.5	2.34	this study
cisplatin	1.6 (control = 0.8)	2.00	^28^

a The cells’ incubation period was maintained at 48 h in all experiments.

**Figure 5:**
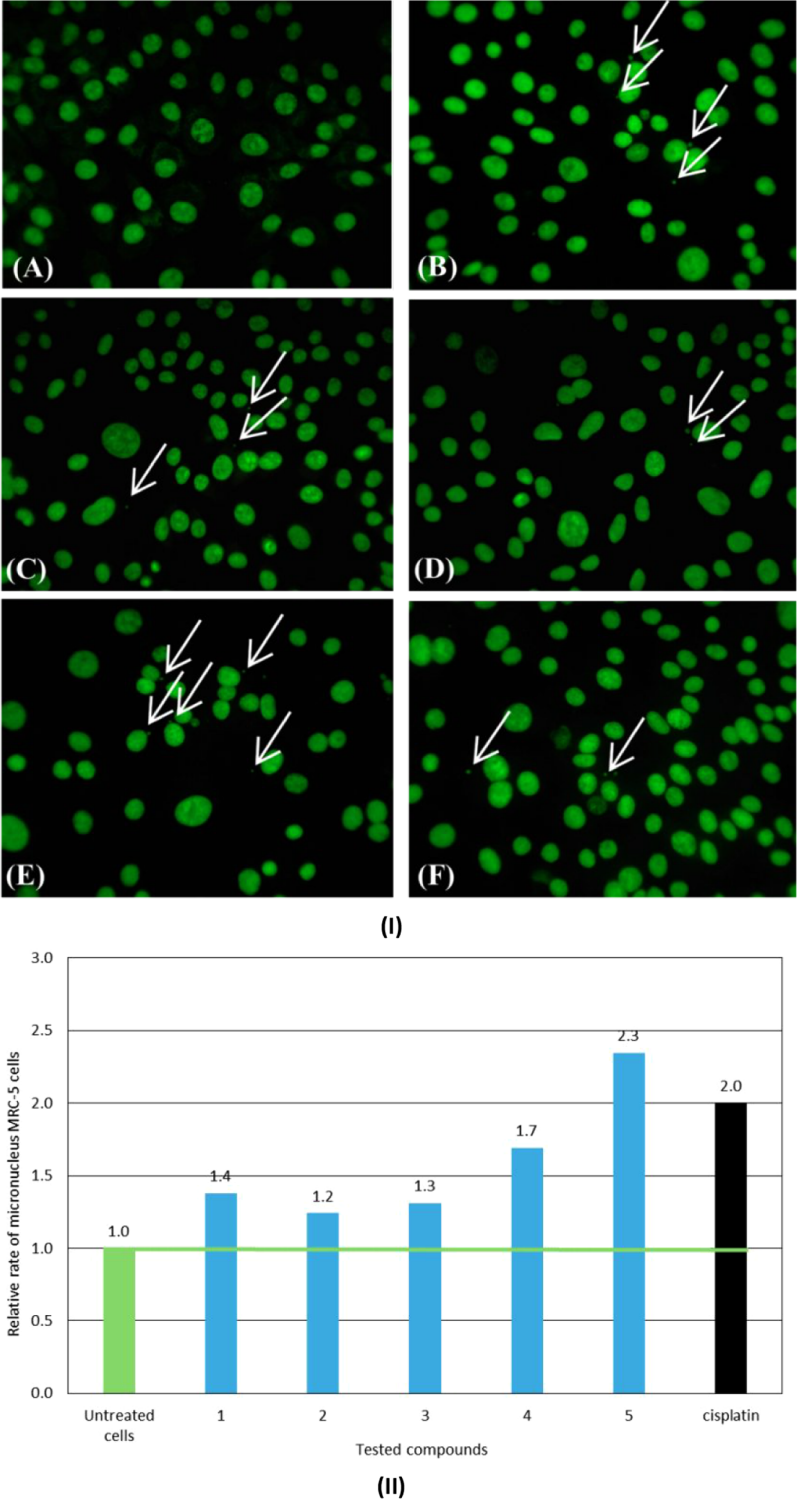
**(I)** Selected pictures with micronucleus formed in nontreated MRC-5 cells (A) and treated with IC_50_ values of **1** (B), **2** (C), **3** (D), **4** (E) and **5** (F) for a period of 48 h; arrows indicate micronucleus in MRC-5 cells. **(II)** The relative ratio of micronuclei (MN) in the in MRC-5 cell cultures after incubation with complexes **1**–**5**, in respect to the corresponding one of the untreated cells.

### In Vitro Mechanism of Action

The *in vitro* mechanism of action of **1**–**5** is clarified by the (i) MCF-7 cells morphology, (ii) AO/EB staining, (iii) cell cycle arrest and (iv) mitochondrial membrane permeabilization tests.

### Cell Morphology Study

The morphological characteristics of MCF-7 cells were evaluated using a phase-contrast inverted microscope after 48 h of exposure to compounds **1**–**5** at their IC_50_ concentrations and compared to the morphology of untreated cells. In MCF-7 cells treated with compounds **1**–**5**, changes in cell morphology were observed, including cell shrinkage, detachment from the culture plate, and rounding. These effects are similar to those seen with cisplatin treatment.^28^ In contrast, the untreated MCF-7 cells exhibited typical morphology, remaining attached to the culture plate, with a stretched shape and maintaining cell-to-cell contact (Figure 6). These findings indicate that compounds **1**–**5** promote apoptosis.

**Figure 6:**
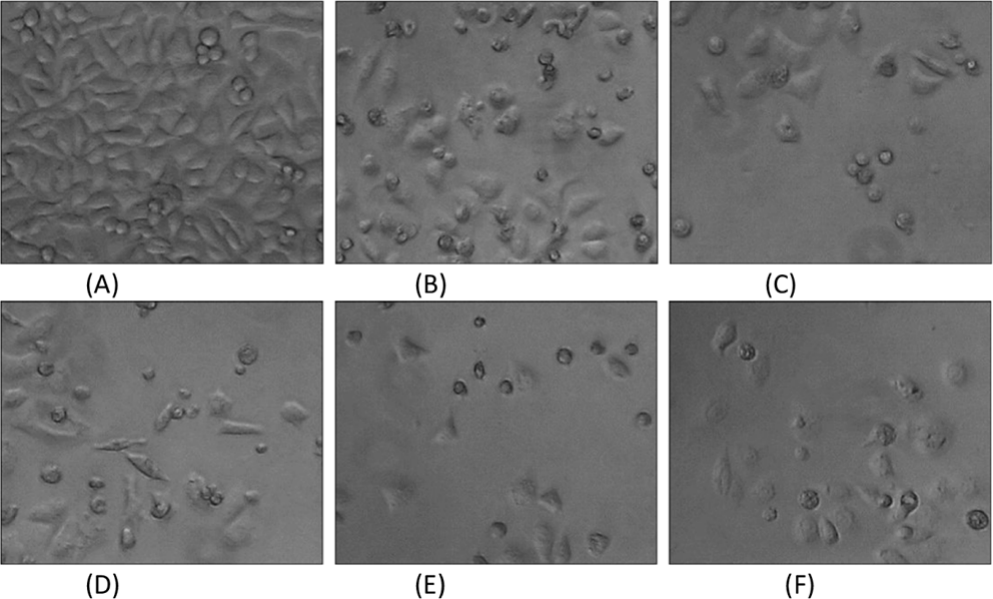
Morphological changes of untreated MCF-7 cells (A), treated MCF-7 cells with **1** (B), **2** (C), **3** (D), **4** (E) and **5** (F).

On the other hand, the untreated MCF-7 cells showed typical morphology, as adherent in culture plate, elongated, and displaying cellular crowding (Figure 6). Therefore, the above observations indicate that compounds **1**–**5** might trigger apoptosis.

### AO/EB Staining Detection of Apoptotic Cells

Apoptotic MCF-7 cells were quantified and characterized using the AO/EB staining assay under fluorescence microscopy, and the results were correlated with previously observed morphological changes in the cells. The type of cell death was evaluated after incubation with complexes **1**–**5** for 48 h. This assay detects nuclear changes based on differences in membrane integrity between live and apoptotic cells. The fluorescent EB dye stains red only those cells that have lost membrane integrity, while the fluorescent AO dye stains both live and dead cells green.^30,38,48,49^ Untreated MCF-7 cells display green radiation emitting nuclei. The percentage of apoptosis in untreated cells was calculated as 11.7 ± 2.8%. In apoptotic cells, varying degrees of changes are observed. Early stages of apoptosis are indicated by greenish-yellow fluorescent nuclei or green-yellow fragments, while late stages are characterized by orange fluorescence or the presence of orange fragments. Overall, the percentage of apoptotic cells in their cell cultures, induced by **1**–**5** upon their treatment, ranges from 46.9% to 64.8% (60.0 ± 5.2% (**1**), 64.8 ± 2.5% (**2**), 46.9 ± 8.0% (**3**), 47.2 ± 5.6% (**4**), and 64.3 ± 8.2% (**5**). The relative rates of apoptotic MCF-7 cells induced by agents **1**–**5** in respect to the corresponding value of untreated-control cells after incubation period of 48 h are 5.13 (**1**), 5.83 (**2**), 4.01 (**3**), 4.03 (**4**) and 5.50 (**5**) (Figures 7 and 8). The corresponding value for cisplatin is 5.88. Therefore, compounds **1**–**5** induce apoptosis with the similar manner with cisplatin.

**Figure 7:**
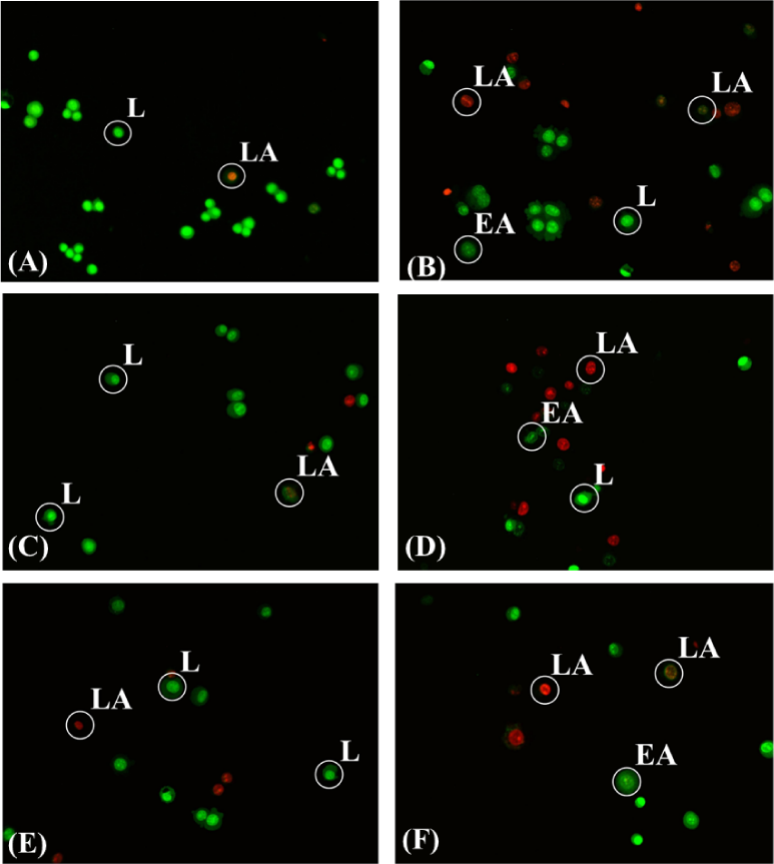
Fluorescence images of the MCF-7 cells incubated with **1**–**5**, untreated cells (A), **1** (B), **2** (C), **3** (D), **4** (E) and **5** (F) for 48 h, 37 °C at IC_50_ values and stained with acridine orange and ethidium bromide (AO/EB). (“L” indicate live cells; “EA” indicate early apoptotic cells; “LA” indicate late apoptotic cells).

**Figure 8:**
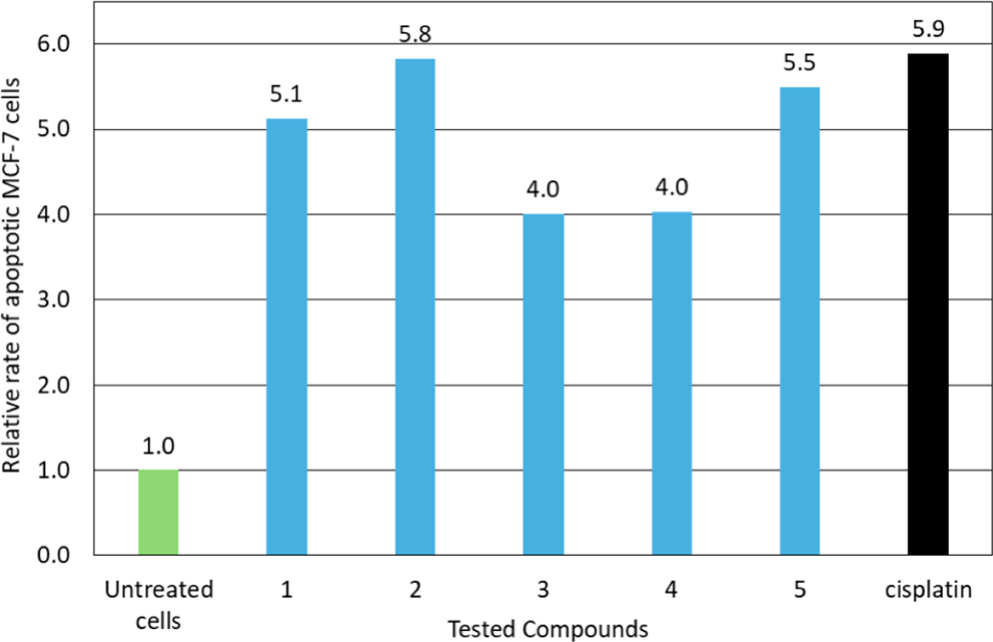
Relative rates of apoptotic MCF-7 cells induced by agents **1**–**5** compared to the corresponding values of untreated control cells.

### Cell Cycle Study

Cell cycle arrest can be utilized to clarify the potential cellular mechanisms through which metallodrugs induce apoptosis.^24,28–30,50^ Metal complexes can interfere with and inhibit cell cycle progression at various stages.^51^ DNA frequency histograms from flow cytometry assays are used to identify apoptotic cells by counting their population in the sub-G_1_ peak.^24,28–30^

Figure 9 shows the effect of **1**–**5** on the cell cycle as the number of MCF-7 cells versus DNA content in sub-G_1_, G_0_/G_1_, S, and G_2_/M phases.

**Figure 9:**
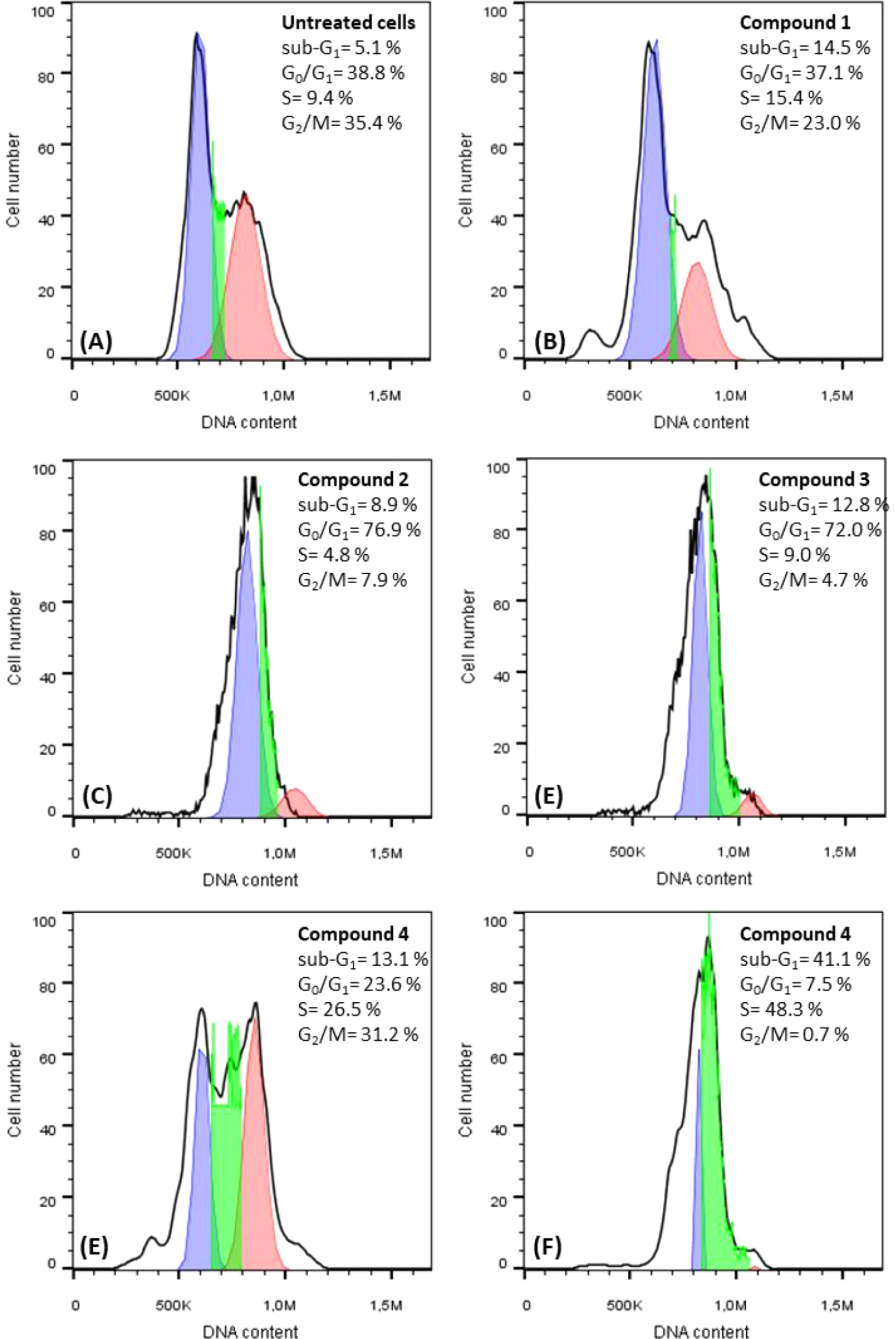
MCF-7 cell populations in the sub-G_1_, G_0_/G_1_, S, and G_2_/M phases of the cell cycle after treatment with compounds **1** (B), **2** (C), **3** (D), **4** (E) and **5** (F) compared to untreated cells (A).

Treatment of MCF-7 cells with the IC_50_ values of **1**–**5** resulted in a significant increase in the proportion of the cells in sub-G_1_ phase (14.5 (**1**), 8.9 (**2**), 12.8 (**3**), 13.1 (**4**) and 41.1% (**5**)), in contrast to untreated cells (5.1%), indicating that the compounds can induce apoptosis. The histogram (Figure 10) illustrates the relative rate in the cells population of the sub-G_1_ phase following the treatment of MCF-7 cells with compounds **1**–**5**, compared to the control cells (untreated cells). Compounds **1–3** and **5** induce a similar increase in the population of MCF-7 cells in the sub-G_1_ phase of the cell cycle, comparable to the effect observed with cisplatin. However, the compound **4** causes a 3-fold higher increase in the MCF-7 cell population in sub-G_1_ phase, compared to both the compounds and cisplatin, indicating significantly greater apoptotic activity, which is even surpassing that of the widely used broad-spectrum agent such as cisplatin.

**Figure 10:**
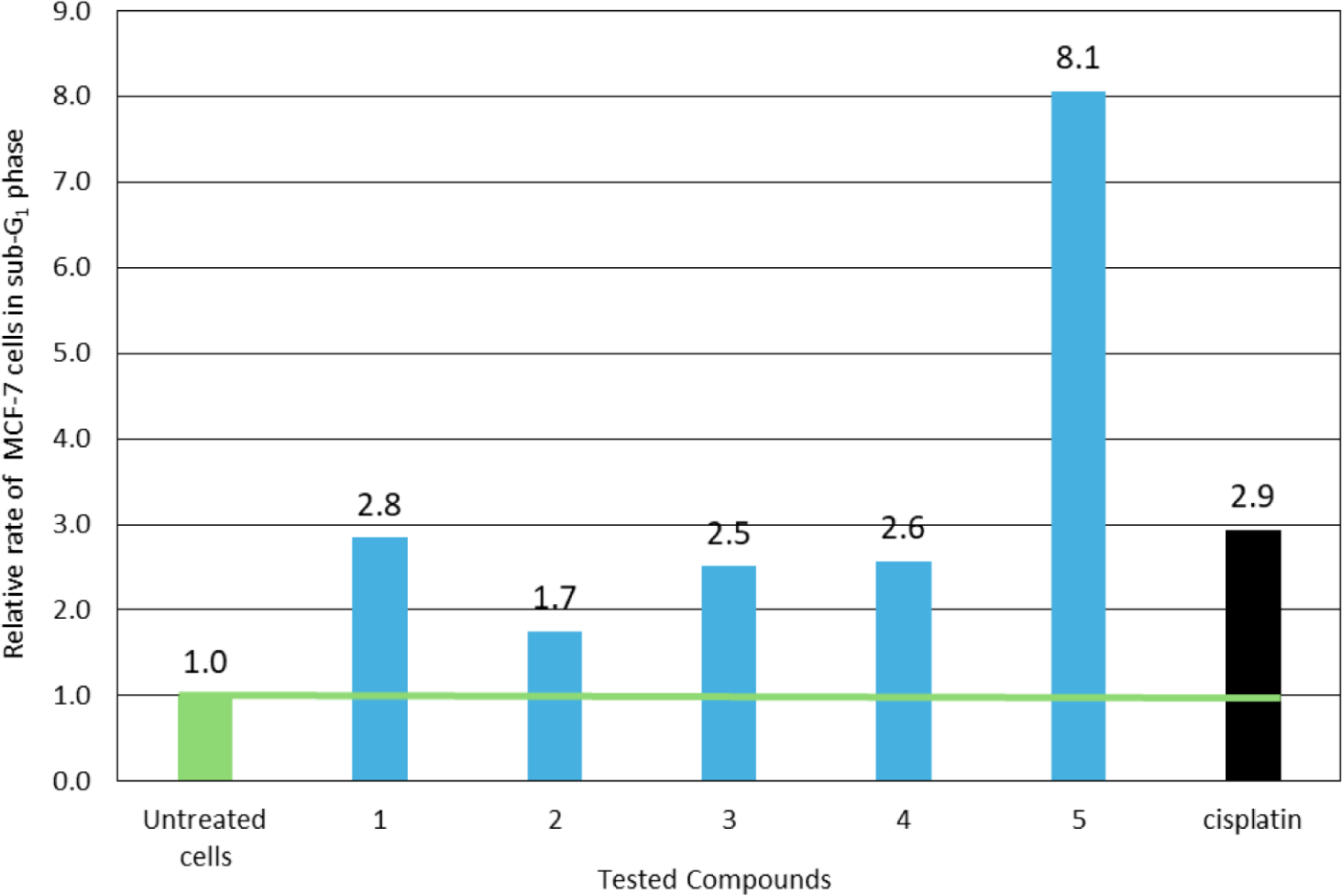
Relative rate in the MCF-7 cell population of the sub-G_1_ phase with compounds **1**–**5**, compared to the untreated cells.

Additionally, compounds **1, 4**, and **5** cause cell cycle arrest in the S-phase, with respective percentages of 15.4% (**1**), 26.5% (**4**), and 48.3% (**5**), compared to 9.4% in untreated cells, thereby inhibiting DNA synthesis.^24,28–30^ However, compounds **2** and **3** induce G_0_/G_1_ phase cell cycle arrest, with 76.9% and 72.0% of cells, respectively, compared to 38.8% in untreated cells.

Cisplatin, on the other hand, is known to induce apoptosis by increasing the MCF-7 cell population in the sub-G_1_ phase, resulting in cell cycle arrest in either the S or G_2_/M phase.^52^ On the contrary the results indicate that the apoptotic sub-G_1_ phase population becomes detectable following treatment with compounds **1**–**5**, and these compounds can induce cell cycle arrest in both the S and G_0_/G_1_ phases.

### Loss of the Mitochondrial Membrane Permeabilization (MPP)

One of the primary targets of metallodrugs is the disruption of mitochondrial membrane permeability in cells.^24,28–30^ Furthermore, treatment of human cancer cells with silver­(I) complexes led to a decrease in MMP, likely triggering the release of mitochondrial proteins that initiate early apoptosis.^24,28–30,53^

After treating MCF-7 cells with compounds **1**–**5** at their IC_50_ values for 48 h, the impact on mitochondrial membrane function was assessed. The assay relies on the fluorescence of a cationic hydrophobic dye, which accumulates in the mitochondrial membrane. Any quenching of the dye’s emitted fluorescence is attributed to the disruption of the mitochondrial membrane, resulting in the release of cytochrome C into the cytosol.^24,28–30^ The percentage of fluorescence quenching in MCF-7 cells treated with compounds **1**–**5** is up to 20.7%, with the specific values being 20.0% (**1**), 20.7% (**2**), 2.9% (**3**), 5.1% (**4**), and 9.6% (**5**). For cisplatin, a fluorescence quenching of 54.9% is observed.^54^ Therefore, this type of formulations either moderately affect the mitochondria or do not interfere with it at all. Consequently, cell morphology, AO/EB staining, cell cycle arrest and MMP studies indicate that compounds **1**–**5** induce apoptosis in MCF-7 cells, primarily through direct or indirect interaction with DNA.

### In Vivo Toxicity Evaluation and Uptake of **1**–**5** by Brine Shrimp 

The *in vivo* toxicity of compounds **1**–**5** was assessed using the assay, a model that shows a strong correlation with toxicity data from rodent and human studies.^55,56^ The U.S. Environmental Protection Agency (EPA) utilizes the zooplanktonic crustacean as a model organism for *in vivo* toxicological testing.^55,56^

The survival rates (%) of larvae exposed to compounds **1**–**5** were assessed at concentrations approximately equal to IC_50_^max^ (≈5 μM), as well as at concentrations up to five times higher than IC_50_^max^, following a 24 h incubation period. For the tested compounds, no toxic effects on the survival of were observed, indicating that the compounds are nontoxic.

 was also observed under an optical microscope. In the case of untreated specimens, their guts appeared empty, and they exhibited a translucent body without the presence of any particles (Figure 11). Upon exposure to compounds **1**–**5**, larvae ingested the compounds, and the gut became nearly fully filled, as indicated by a dark line visible inside the gut (Figure 11). However, this accumulation did not lead to any mortality. Moreover, the accumulated compounds, such as compound **3** at a concentration of 25 μM, were excreted by (Figure 11).

**Figure 11:**
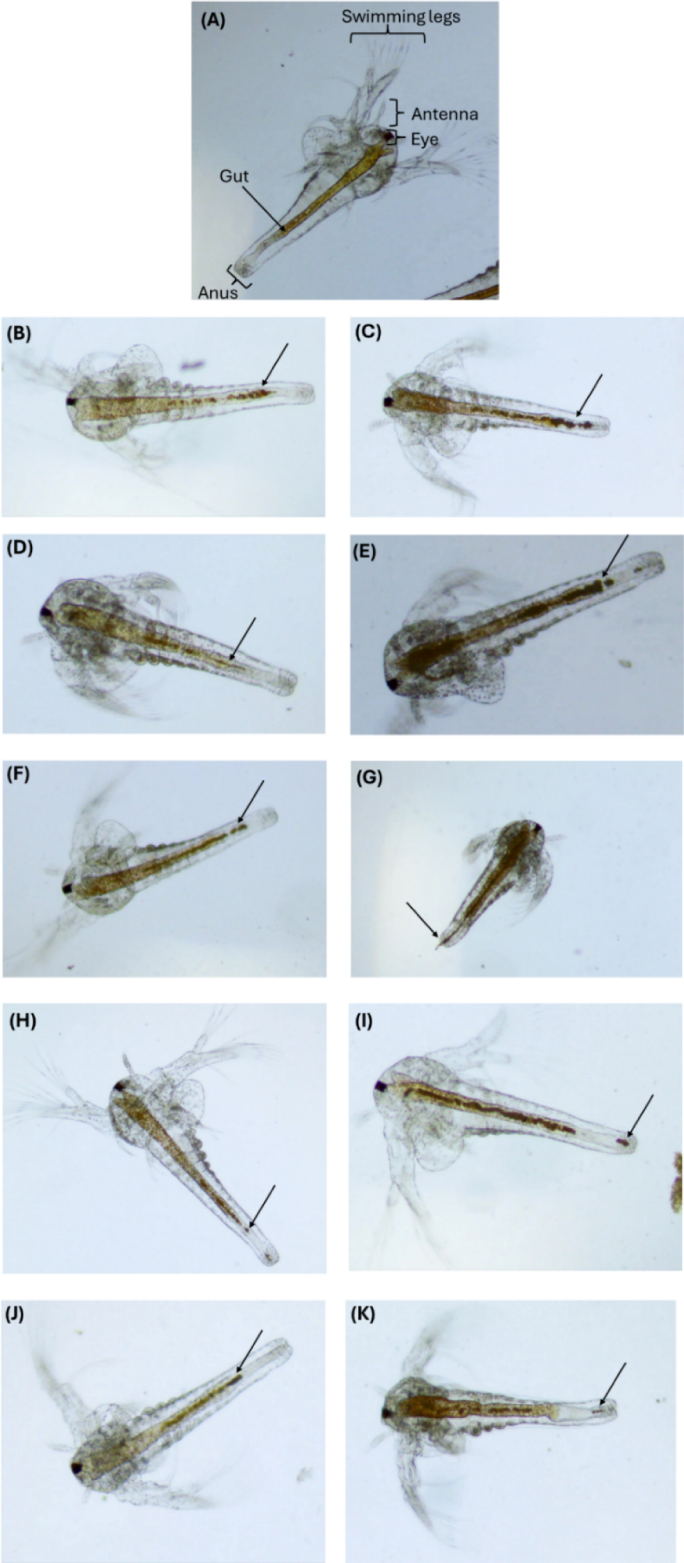
Optical microscope images of the ingestion of **1**–**5** (**1** at 5 (B) and 25 (C), **2** at 5 (D) and 25 (E), **3** at 5 (F) and 25 (G), **4** at 5 (H) and 25 (I), and **5** at 5 (K) and 25 (L) μΜ) (black arrows) by larvae after 24 h exposure to at 5 and 25 μΜ. The gut is empty in the control (**A**), **1**–**5** is visible as a dark line inside the gut of treatment (**B–K**).

In general, brine shrimp are nonselective filter feeders and can easily ingest particles up to 50 μm in diameter.^57,58^ The accumulation of silver nanoparticles in the gut of has previously been reported in studies on the ecotoxicity of various other nanoparticles.^56–58^

### Ex Vivo Mechanism Studies

The molecular mechanism of action of **1–5** was further studied by their (i) binding affinity toward the calf thymus (CT)-DNA and (ii) inhibitory activity against lipoxygenase (LOX), an enzyme which is mainly distributed in mitochondrion and it is associated with its function.

### CT-DNA Binding Affinity Studies

In order to elucidate the mechanism of action of compounds **1**–**5** against MCF-7 cells, their interaction with CT-DNA was investigated using UV absorption and fluorescence spectroscopy. The UV absorption spectra of compounds **1**–**5** were recorded both in the presence and absence of CT-DNA (Figure S27). Gradual additions of compounds **1**–**5** to the DNA solution caused hyperchromism, suggesting groove binding of the complexes to DNA.^26,28,30^ The percentage of hyperchromism observed for **1**–**5** are 4.0 (**1**), 7.6 (**2**), 28.0 (**3**), 8.5 (**4**) and 1.2 (**5**) %, respectively. Moreover, the binding constants (*K*_b_) of **1**–**5** toward CT-DNA were evaluated by monitoring the absorbance changes of the UV spectra of complexes (25 × 10^–6^ M), at 300–310 nm, with increasing concentrations of CT-DNA (10–100 μΜ) (Figure S28). The calculated *K*_b_ values for **1**–**5** are (10.5 ± 1.8) × 10^4^ (**1**), (6.3 ± 0.6) × 10^4^ (**2**), (11.3 ± 1.6) × 10^4^ (**3**), (7.5 ± 1.0) × 10^4^ (**4**) and (10.2 ± 1.1) × 10^4^ (**5**) M^–1^, respectively.

By comparing the *K*_b_ of **1**–**5** toward CT-DNA with their IC_50_ against human breast cancer cells either with hormone receptors (MCF-7) or without (MDA-MB-231), compound **5** shows the lowest IC_50_ value against MCF-7, with a high *K*_b_ (10.2 × 10^4^ M^–1^). This indicates a good correlation between strong DNA binding and low IC_50_. Compound **3** exhibits the best activity against MDA-MB-231 with the lowest IC_50_ and the highest *K*_b_ (11.3 × 10^4^ M^–1^). Thus, compounds **3** (p-F-PODTH) and **5** (m-Cl-PODTH) stand out due to their high *K*_b_ and relatively low IC_50_ values across the different cell lines, suggesting that stronger DNA binding correlates with better biological activity in these cases. This is due to the structure of these compounds, which allows the formation of intermolecular bonds between the electronegative halogens (X) of the *p*- or *m*-X-PODTH and the electropositive atoms of neighboring molecules, such as the nitrogen or oxygen atoms in nucleotides and DNA bases.^26^

A similar behavior was observed in the case of *p*-hydroxy-benzoic acid and its silver complex with TPP, [Ag­(p-Hbza)­(TPP)_2_] (where *p*-HbzaH = 4-hydroxybenzoic acid). In this system, the polar–OH group favors the formation of hydrogen bonds with nitrogen or oxygen atoms in nucleotides and DNA bases. These interactions contribute significantly to the stabilization of the complex.^26^

The DNA-complex binding properties can also be studied by fluorescent spectroscopy can also be studied by fluorescent spectroscopy.^24,28–30^ EB emits intense fluorescence in the presence of DNA due to its strong intercalation between adjacent DNA base pairs. As EB binds to DNA within the minor groove, its displacement by a compound could indicate either intercalative binding or minor groove interaction, among other possibilities.^24,28–30^ The emission spectra of the CT-DNA-EB complex were recorded in both the absence and presence of compounds **1**–**5** (Figure S29). The fluorescence intensity at λ_max_^em^ = 588 nm was quenched by 60.2 (**1**). 58.5 (**2**), 62.0 (**3**), 61.9 (**4**) and 61.6 (**5**) %, for **1**–**5** respectively, compared to the initial fluorescence intensity of the EB-DNA solution.

The calculated *K*_app_ values are 6.3 × 10^4^ (**1**), 6.7 × 10^4^ (**2**), 7.0 × 10^4^ (**3**), 6.9 × 10^4^ (**4**) and 6.5 (**5**) × 10^4^ M^–1^, respectively for **1**–**5**. These values suggest minor groove binding mode for **1**–**5**, since they lie in the range 10^4^–10^5^ M^–1^.^24,28–30^ Intercalation typically results in high *K*_app_ values, as observed with EB, where *K*_app_ can reach up to 10_7_ M^–1^. Therefore, the lower *K*_app_ values for compounds **1**–**5** suggest that they bind through a minor groove binding mode.^24,28–30,59^ .

The viscosity of a DNA solution is highly sensitive to structural changes in DNA and is widely used to investigate the binding modes of agents interacting with it.^30^ When a molecule binds to DNA, it alters the DNA’s length, thereby affecting the solution’s viscosity. Specifically: [i] intercalation results in a significant increase in the relative viscosity of the DNA–agent solution, due to unwinding and elongation of the DNA double helix;^30^ [ii] groove binding or electrostatic interactions, by contrast, do not markedly change the DNA length, leading to minimal effects on viscosity;^30^ [iii] cleavage of the DNA strands by an agent produces shorter DNA fragments, resulting in a notable decrease in viscosity;^30^ and [iv] covalent bond formation between an agent and DNA—as seen in cisplatin–DNA complexes—induces bending of the DNA backbone, shortening the helical axis and thus decreasing the solution’s viscosity.^30^

The values of the relative specific viscosity, (η/η_0_)^1/3^, were plotted against the ratio *r* = [complex]/[DNA] (Figure 12). Compounds **1**–**5** were no alter the relative viscosity of the DNA solution, either decreasing it or increasing it, indicating possible groove binding or electrostatic interactions with DNA.^30^

**Figure 12:**
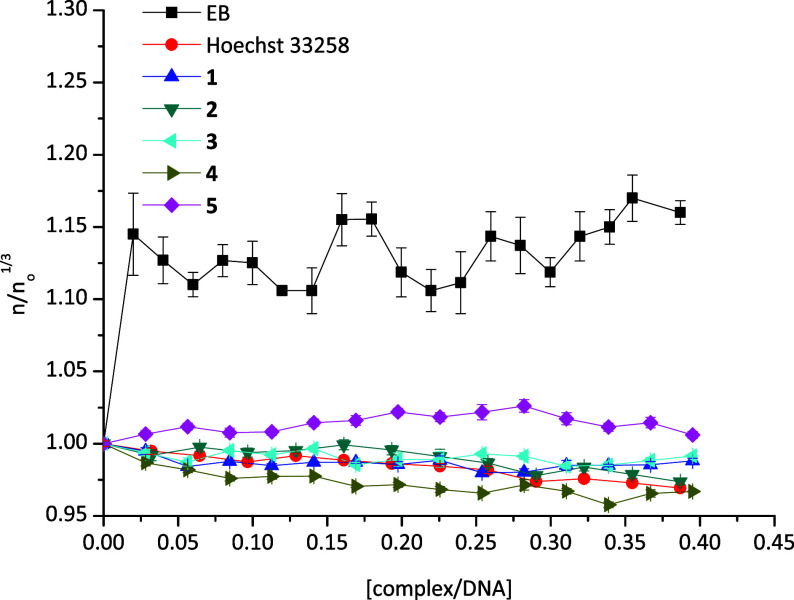
Relative viscosity of CT-DNA with increasing concentrations of **1**–**5**, Hoechst 33258 and EB ([DNA] = 10 mM, *r* = [compound]/[DNA], η is the viscosity of DNA in the presence of the compounds and η_o_ is the viscosity of DNA alone).

In order to ascertain the groove binding of the compounds toward DNA, the viscosity of their solution is compared with the corresponding one of the minor groove binders or intercalators (such as minor groove binding agent Hoechst 33258 and intercalator EB (Figure 12). For DNA lengthening studies, the model proposed^60^ predicts that a plot of (η/η_0_)^1/3^ versus *r* yield a slope close to 1 for a “classical” intercalator. This prediction is based on the estimated 3.4 Å increase in DNA helix length upon insertion of an intercalator between adjacent base pairs. In practice, most classical intercalators produce slopes between 0.5 and 1 in such plots.^60^ Classical groove binders, on the other hand, such as Hoechst 33258, typically result in a slope of zero. The results confirmed our assumption for groove binding of **1**–**5** to DNA.

### Study of the Inhibitory Activity **1**–**5** toward the Peroxidation of Linoleic Acid by the LOX Enzyme

To explore whether complexes **1**–**5** interact with mitochondria-associated enzymes, interaction studies with LOX were conducted to gain deeper insights into the underlying mechanisms. The enzyme LOX is primarily distributed in the mitochondria and is involved in the inflammatory process through the peroxidation of arachidonic acid to produce leukotrienes.^19,21–26,61^ Inhibition of LOX by metallodrugs induces cell apoptosis^19,21–26,61^ either through the disruption of mitochondrial membrane integrity, which subsequently leads to the release of cytochrome *c* into the cytosol,^62^ or by increasing mitochondrial hydrogen peroxide levels, which would otherwise be consumed during the peroxidation reaction.^63,64^

Figure 13 illustrates the relationship between LOX activity (A%) and the negative logarithm of inhibitor concentration (pLog­(C)). The results demonstrate a significant reduction in LOX catalytic activity at low inhibitor concentrations. The IC_50_ values for compounds **1**–**5** against LOX range from 1.8 to 7.5 μM, (2.5 (**1**), 7.3 (**2**), 6.8 (**3**), 7.5 (**4**) and 1.8 (**5**) μΜ)) (Table S4). indicating notably higher inhibitory activity compared to cisplatin (IC_50_= 65.9 μM).^23^

**Figure 13:**
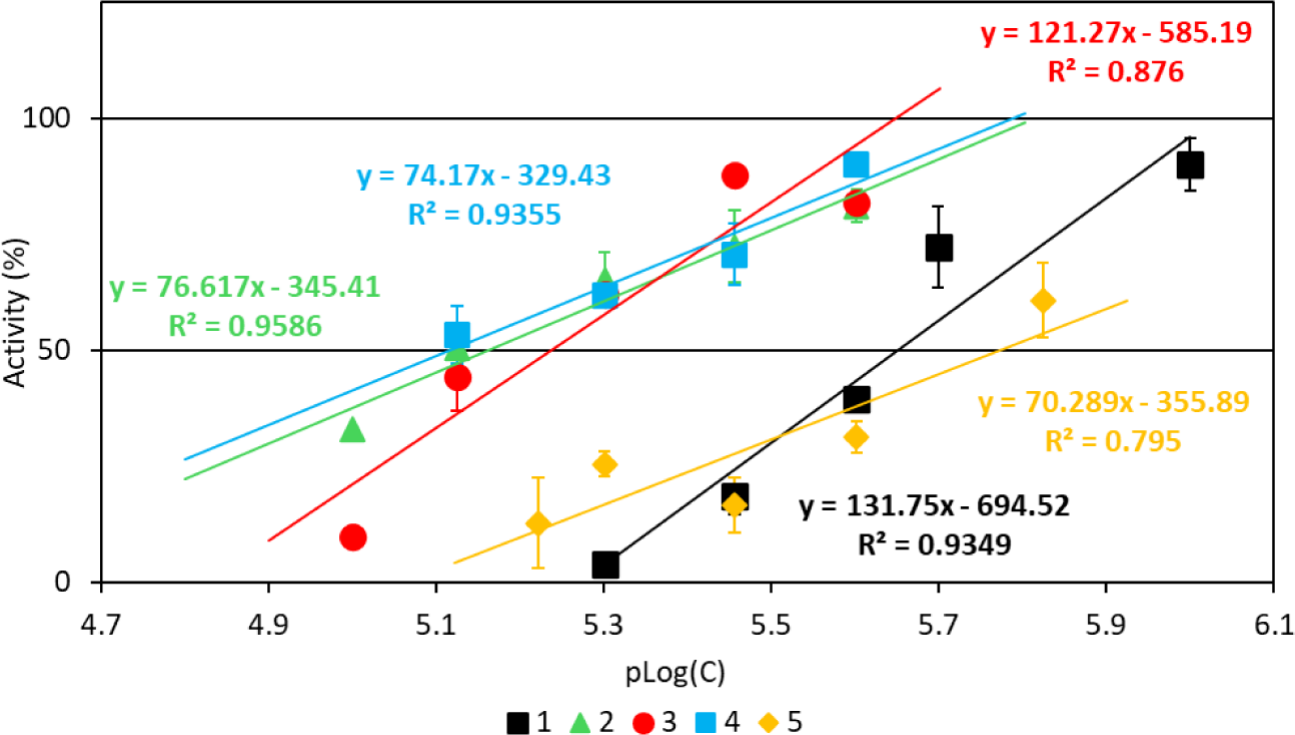
LOX activity (A%) vs negative logarithm of inhibitor (**1**–**5**) concentration (pLog­(*C*)).

Interestingly, the order of LOX inhibitory potency for compounds **1**–**5** (**5** < **1** < **3** < **2** < **4**) mirrors the sequence observed for their antiproliferative activity against MCF-7 cells (**5** < **1** < **3** < **2** < **4**), but differs from the order found against MDA-MB-231 cells (**3** < **5** < **2** < **1** < **4**). Therefore, in general, compounds exhibiting strong LOX inhibitory activity also display potent antiproliferative effects, suggesting that their mechanism of action may involve LOX inhibition, which in turn activates the intrinsic apoptotic pathway in cancer cells.^19,21–26,61^

### Structure–Activity Relationship (SAR) Analysis

A multiple linear regression (MLR) analysis was conducted to investigate the relationship between the antiproliferative activity of the compounds against MCF-7 cells (expressed as pIC_50_) and selected molecular descriptors, including LOX inhibitory activity (pIC_50_^LOX^), molecular weight (Log­(MW)), lipophilicity (LogP), and DNA binding affinity (Log­(*K*_b_)) (Table S4). The analysis was performed on a set of 11 silver­(I) complexes, utilizing experimental and computational data (Table S5).

The derived regression equation is as follows:

$${{\mathrm{pIC}}_{50}}^{\mathrm{MCF‐7}}=-0.11-0.02\times {{\mathrm{pIC}}_{50}}^{\mathrm{LOX}}+0.24\times \log\left(\mathrm{MW}\right)+0.02\times \log P+0.98\times \log\left(K_{b}\right)$$

The model exhibited a strong fit, with an *R* ^2^ value of 0.88, indicating that approximately 88% of the variability in pIC_50_^MCF‑7^ is explained by the selected descriptors. Among the evaluated descriptors, log­(*K*_b_) (the logarithm of the DNA binding constant) was identified as the only statistically significant predictor (*p* = 0.001), demonstrating a strong positive correlation with the antiproliferative activity. This finding highlights the critical role of DNA binding affinity in modulating the cytotoxic effects of these silver­(I) complexes against MCF-7 cells. In contrast, the other descriptors, including pIC_50_^LOX^, Log­(MW), and Log *P*, did not exhibit statistically significant contributions to the model (*p* > 0.05), though they may still have synergistic effects within the multivariate framework. Overall, this analysis underscores the dominant influence of DNA binding affinity (log *K*_b_) on the antiproliferative activity of the studied complexes, suggesting that enhancing the DNA binding capability could be a key strategy for improving their cytotoxic potential.

## Conclusion

The combination of mitochondriotropic agent like triphenyl phosphine with 5-aryl-1,3,4-oxadiazole-2-thione derivatives via the silver ions leads in the formation of novel effective cancer chemotherapeutic metallodrugs.

Compounds **1**–**5** and their ligands **1a**–**5a** were synthesized and tested against MCF-7 and MDA-MB-231. Compounds **1a**–**5a** show IC_50_ values greater than 30 μM against both MCF-7 and MDA-MB-231, suggesting they are largely inactive against these cells. To the contrary compounds **1**–**5** exhibit lower IC_50_ values, indicating notable activity (Tables 1 and S4). Compounds **5** and **3** show the strongest activity against MCF-7 (**5:** 1.69 μM) and MDA-MB-231 (**3:** 2.52 μM) respectively. Compounds with halogen substituents (F or Cl) generally show lower IC_50_ values than the unsubstituted compound (**1**, H−). The presence of halogen substituents, especially at the meta- or para-positions on the phenyl ring, improves the cytotoxic activity against both MCF-7 and MDA-MB 231 cell lines. m-Cl- (compound **5**) and p-F**-** (compound **3**) are the most effective modifications for enhancing cytotoxicity. Cisplatin has much higher IC_50_ values for MCF-7 (5.5 μM) and especially for MDA-MB-231 (26.7 μM), showing that compounds (**1**–**5**) are more effective than cisplatin against MCF-7 (2–3 fold more effective) but significantly more effective against MDA-MB-231 (6–11 fold more effective).

Moreover, a structure–activity relationship (SAR) study is made by comparing their structural parameters (bond distances and angles), along with their physical properties (molecular weight (MW), lipophilicity (Log *P*), DNA binding affinities (*p*(*K*_b_)) with their cytotoxic activity against breast cancer cell lines (MCF-7 and MDA-MB-231) (Tables 1 and S4). A comprehensive SAR analysis was performed using both correlation and multivariate regression models. Our findings demonstrate that the cytotoxic potency of these complexes is significantly influenced by their metal–ligand interactions, bond distances, and coordination geometries. Specifically, shorter Ag–P and Ag–S distances were found to enhance cytotoxicity in both MCF-7 and MDA-MB-231 cells, indicating that strong metal-phosphine and metal-thione bonding plays a key role in biological activity.

Additionally, smaller P–Ag–P and P–Ag–S bond angles were correlated with enhanced cytotoxic effects, particularly in MDA-MB-231 cells, highlighting the importance of compact coordination geometries in determining anticancer efficacy. Stronger DNA binding (higher *p*(*K*_b_)) significantly improves cytotoxicity, while no secure conclusions can be made for the influence of Log *P* due to the low number of entries. However, higher Log *P* (lipophilicity) weakens cytotoxicity, possibly due to reduced solubility or altered cellular uptake.

## Experimental Section

### Materials for Synthesis and Chemical Studies

All solvents (Sigma-Aldrich, Merck) were of reagent grade and used without further purification. Silver nitrate was obtained from Degussa (Berlin, Germany), triphenylphosphine from Sigma-Aldrich, and dimethyl sulfoxide from Riedel-de Haën.

### Materials for the Biological Studies

Dulbecco’s modified Eagle’s medium (DMEM), fetal bovine serum, and phosphate-buffered saline (PBS) were obtained from Sigma-Aldrich. Trypsin-EDTA and l-glutamine were purchased from Biowest, and penicillin-streptomycin from Gibco (Glasgow, UK). Sulforhodamine B was sourced from Alfa Aesar, while propidium iodide, RNase A, and tris base were from Sigma-Aldrich. Dimethyl sulfoxide and boric acid were supplied by Riedel-de Haën. For toxicity experiments, brine shrimp eggs () were obtained from Ocean Nutrition, and sea salt from Tropic Marin.

### Instruments Used

Melting points were determined in open tubes using a Stuart Scientific apparatus and are uncorrected. Elemental analyses for carbon, hydrogen, nitrogen and sulfur were conducted with a Carlo Erba EA MODEL 1108 elemental analyzer. IR spectra within the 4000–370 cm^–1^ range were obtained on a Cary 670 FTIR spectrometer (Agilent Technologies). The ^1^H NMR spectra were recorded on a Bruker AC 400 MHz FT-NMR instrument in DMSO-*d*_6_ solution. Electronic absorption spectra were measured with a UV-1600 PC series spectrophotometer (VWR), while fluorescence spectra were acquired using a Jasco FP-8200 Fluorescence Spectrometer. XRF measurements were performed on a Rigaku NEX QC EDXRF analyzer (Austin, TX, USA). For cell cycle arrest experiments, a FACS Calibur flow cytometer (Becton Dickinson, San Jose, CA, USA) was used. For viscosity measurements, low volume rotational viscometer was used.

### Synthesis and Crystallization of **1a**–**5a**

The ligands were synthesized as previously described with slight modifications.^34,35^ In brief: potassium hydroxide (50 mmol) was dissolved in 200 mL of absolute ethanol, and the appropriate benzohydrazide (50 mmol) was added to the stirred solution, followed by an excess of CS_2_ (70 mmol), (precautions: CS_2_ is a volatile, flammable, and toxic chemical, and its use requires strict precautions to ensure safety). The mixture was heated under reflux for 2–3 h. Afterward, the solvents were removed under reduced pressure, and the resulting solid was dissolved in water, cooled on ice, and acidified with HCl to precipitate a solid. This solid was filtered, dried, and recrystallized from a 1:1 ethanol–water mixture. Crystals of compounds **1a–5a** suitable for X-ray analysis were obtained by slow evaporation of a DMSO solution over 2–5 days.

**1a**: white powder; melting point: 218–220 °C; Elemental analysis found: C: 54.25; H: 3.24, N: 15.82, S: 18.12%; calculated for C_8_H_6_N_2_OS: C: 53.92; H: 3.39; N: 15.72; S: 17.99%. IR (cm^–1^): 3055 (br), 2922 (br), 1609 (s), 1573 (s), 1507 (vs), 1447 (s), 1336 (vs), 1275 (vs), 1182 (vs), 1156 (s), 1080 (s), 1060 (s), 966 (vs), 940 (s), 748 (m), 634 (vs), 551 (vs), 494 (m), 481 (m). ^1^H NMR (ppm) in DMSO-*d*_6_: 7.90–7.88 ppm (d, H^2′,6′^[Ph‑]), 7.67–7.58 (m, H^3′,4′,5′^[Ph‑]).

**2a**: white powder; melting point: 143–145 °C; Elemental analysis found: C: 49.25; H: 2.24, N: 14.88; S: 16.15%; calculated for C_8_H_5_N_2_OSF: C: 48.98; H: 2.57; N: 14.28; S: 16.34%. IR (cm^–1^): 3078 (br), 2937 (br), 1613 (m), 1598 (m), 1485 (vs), 1424 (m), 1406 (m), 1342 (vs), 1282 (m), 1229 (m), 1172 (m), 1153 (m), 1093 (m), 1073 (s), 1033 (m), 1011 (m), 961 (vs), 935 (vs), 823 (m), 765 (m), 721 (vs), 686 (s), 573 (m), 501 (s), 465 (m), 421 (m), ^1^H NMR (ppm) in DMSO-*d*_6_: 7.93–7.89 (m, H^6′^[*o*-F-Ph-]), 7.72–7.41 (m, H^3′,4′,5′^[*o*-F-Ph-]).

**3a**: yellow powder; melting point: 172–174 °C; Elemental analysis found: C: 48.76; H: 2.55, N: 14.09; S: 16.54%; calculated for C_8_H_5_N_2_OSF: C: 48.98; H: 2.57; N: 14.28; S: 16.34%.%. IR (cm^–1^): 3070 (br), 2930 (br), 1674 (m), 1653 (m), 1603 (s), 1560 (m), 1540 (m), 1505 (vs), 1422 (s), 1281 (m), 1228 (m), 1187 (m), 1156 (s), 1093 (s), 1070 (s), 1012 (s), 963 (m), 940 (m), 832 (vs), 766 (m), 721 (m), 984 (s), 948 (m), 633 (m), 609 (s), 537 (m), 500 (vs), 470 (s), 419 (vs). ^1^H NMR (ppm) in DMSO-*d*_6_: 7.98–7.93 (m, H^2′,6′^[*p*-F-Ph‑]), 7.46–7.42 (m, H^3′,5′^[*p*-F-Ph‑]).

**4a**: white powder; melting point: 168–170 °C; Elemental analysis found: C: 45.50; H: 2.25, N: 13.49; S: 15.34%; calculated for C_8_H_5_N_2_OSCl: C: 45.19; H: 2.37; N: 13.17; S: 15.08%. IR (cm^–1^): 3276 (m), 1606 (s), 1565 (m), 1481 (m), 1461 (vs), 1333 (s), 1280 (m), 1240 (m), 1175 (s)­m 1154 (s), 1127 (m), 1094 (m), 1068 (vs), 1029 (vs), 960 (m), 948 (m), 869 (m), 765 (vs), 766 (vs), 721 (s), 683 (m), 654 (m), 628 (m), 561 (m), 510 (s), 454 (s), 417 (m). ^1^H NMR (ppm) in DMSO-*d*_6_: 7.93–7.91 (m, H^6′^[*o*-Cl-Ph-]), 7.72–7.55 (m, H^3′,4′,5′^[*o*-Cl-Ph-]).

**5a**: white powder; melting point: 181–183 °C; Elemental analysis found: C: 45.00; H: 2.52, N: 13.20; S: 15.22%; calculated for C_8_H_5_N_2_OSCl: C: 45.19; H: 2.37; N: 13.17; S: 15.08% IR (cm^–1^): 3044 (br), 2919 (br), 1610 (m), 1586 (m), 1564 (m), 1500 (m), 1470 (m), 1410 (m), 1344 (s), 1283 (s), 1180 (vs), 1105 (s), 1061 (s), 967 (s), 948 (s), 883 (s), 799 (s), 751 (m), 707 (vs), 671 (m), 650 (m), 557 (m), 497 (m), 434 (m). ^1^H NMR (ppm) in DMSO-*d*_6_: 7.87–7.84 (m, H^2′,6′^[*m*-Cl-Ph-]), 7.73–7.61 (m, H^4′,5′^[*m*-Cl-Ph-]).

### Synthesis of **1**–**5**

A suspension of 0.5 mmol of ligands **1a–1e** (0.089 g for **1a**, 0.098 g for **1b** and **1c**, and 0.106 g for **1d** and **1e**) was treated with 0.5 mL of 1 N KOH. The resulting solution was added dropwise to a solution of silver nitrate (0.5 mmol, 0.085 g) in 5 mL of water at room temperature. A white precipitate formed immediately, which was then filtered and dried under vacuum over calcium chloride. The resulting powder was dissolved in 10 mL of DMSO, and solid triphenylphosphine (1.5 mmol, 0.393 g) was added. The mixture was stirred for 30 min and kept in the dark. Crystals of compounds **1**–**5**, suitable for X-ray analysis, were obtained by slow evaporation of the solution over 2–5 days.

**1**: colorless crystals; melting point: 129–131 °C; Elemental analysis found: C: 69.72; H: 4.55, N: 2.71, S: 3.05, Ag = 10.22%; calculated for C_62_H_50_AgN_2_OP_3_S: C: 69.47; H: 4.70, N: 2.61, S: 2.99, Ag = 10.06%. IR (cm^–1^): 3400 (br), 1653 (m), 1507 (m), 1477 (m), 1433 (m), 1411 (s), 1312 (m), 155 (m), 1092 (m), 1013 (vs), 952 (s), 846 (m), 741 (s), 688 (s), 501 (s), 487 (s). ^1^H NMR (ppm) in DMSO-*d*_6_: 7.47–7.39 (m, H^2′,6′^[Ph-]), 7.33–7.24 (m, H^3′,4′,5′^[Ph-] and *p*-,*m*-,*o*-H­[Ph-] of TPP). UV–vis (DMSO): λ = 268 nm (log ε = 4.45).

**2**: colorless crystals; melting point: 140–144 °C; Elemental analysis found: C: 68.45; H: 4.37, N: 2.71, S: 3.08, Ag = 9.50%. calculated for C_62_H_49_AgFN_2_OP_3_S: C: 68.33; H: 4.53, N: 2.57, S: 2.94, Ag = 9.90%. IR (cm^–1^): 3054 (br), 2115 (br), 1476 (s), 1432 (s), 1408 (s), 1308 (m), 1120 (m), 1089 (m), 1027 (m), 994 (m), 741 (s), 690 (vs), 542 (s), 503 (s), 487 (s). ^1^H NMR (ppm) in DMSO-*d*_6_: 7.65–7.39 (m, H^6′^[*o*-F-Ph-]), 7.33–7.23 (m, H^3′,4′,5′^[*o*-F-Ph-] and *p*-,*m*-,*o*-H­[Ph-] of TPP). UV–vis (DMSO): λ = 265 nm (log ε = 4.52).

**3**: yellowish crystals; melting point: 120–123 °C; Elemental analysis found: C: 68.15; H: 4.30, N: 2.83, S: 3.10, Ag = 9.59%. calculated for C_62_H_49_AgFN_2_OP_3_S: C: 68.33; H: 4.53, N: 2.57, S: 2.94, Ag = 9.90%. IR (cm^–1^): 3055 (br), 2086 (br), 1496 (m), 1476 (s), 1418 (vs), 1308 (m), 1219 (s), 1180 (m), 1157 (m), 1129 (s), 1091 (s), 1052 (m), 10–24 (m), 995 (m), 948 (m), 925 (m), 841 (s), 744 (vs), 691 (vs), 624 (m), 493 (vs), 422 (m). ^1^H NMR (ppm) in DMSO-*d*_6_: 7.51–7.40 (m, H^2′,6′^[*p*-F-Ph-]), 7.35–7.25 (m, H^3′,5′^[*p*-F-Ph-] and *p*-,*m*-,*o*-H­[Ph-] of TPP). UV–vis (DMSO): λ = 268 nm (log ε = 4.49).

**4**: colorless crystals; melting point: 93–95 °C; Elemental analysis found: C: 68.45; H: 4.55, N: 2.34, S: 3.04, Ag = 9.35%; calculated for C_62_H_49_AgClN_2_OP_3_S: C: 68.31; H: 4.46, N: 2.53, S: 2.90, Ag = 9.75%. IR (cm^–1^): 3064 (br), 2929 (br), 1699 (m), 1652 (m), 1601 (m), 1559 (m), 1540 (m), 1506 (s), 1478 (m), 1421 (s), 1282 (m), 1228 (m), 1157 (s), 1092 (vs), 1071 (s), 1034 (m), 1012 (m), 940 (m), 832 (s), 745 (m), 690 (vs), 631 (m), 610 (s), 540 (m), 503 (vs), 471 (m), 419 (vs). ^1^H NMR (ppm) in DMSO-*d*_6_: 7.57–7.42 ppm (m, H^6′^[*o*-Cl-Ph‑]), 7.39–7.32 (m, H^3′,4′,5′^[*o*-Cl-Ph-] and *p*-,*m*-,*o*-H­[Ph-] of TPP). UV–vis (DMSO): λ = 265 nm (log ε = 4.49).

**5**: colorless crystals; melting point: 137–139 °C; Elemental analysis found: C: 68.22; H: 4.35, N: 2.78, S: 2.80, Ag = 9.96%; calculated for C_62_H_49_AgClN_2_OP_3_S: C: 68.31; H: 4.46, N: 2.53, S: 2.90, Ag = 9.75%. IR (cm^–1^): 3052 (br), 2101 (br), 1476 (s), 1432 (s), 1407 (s), 1308 (m), 1157 (m), 1133 (m), 1092 (s), 1068 (m), 1025 (m), 997 (m), 916 (m), 849 (m), 740 (vs), 688 (vs), 509 (vs), 486 (vs), 437 (m). ^1^H NMR (ppm) in DMSO-*d*_6_: 7.47–7.36 (m, H^2′,6′^[*m*-Cl-Ph-]), 7.33–7.24 (m, H^4′,5′^[*m*-Cl-Ph-] *p*-,*m*-,*o*-H­[Ph-] of TPP). UV–vis (DMSO): λ = 268 nm (log ε = 4.62).

### X-ray Structure Determination

Crystals of **1** (0.15 × 0.21 × 0.27 mm), **2** (0.05 × 0.15 × 0.38 mm), **3** (0.11 × 0.23 × 0.40 mm), **4** (0.20 × 0.20 × 0.46 mm), **5** (0.07 × 0.32 × 0.49), **2a** (0.06 × 0.15 × 0.42), **4a** (0.08 × 0.15 × 0.52) and **5a** (0.07 × 0.09 × 0.34) were taken from the mother liquor and immediately cooled to −103 °C (**1** and **2a**) and the rest to −113 °C. Diffraction measurements of the above compounds were made on a Rigaku R-AXIS SPIDER Image Plate diffractometer using graphite monochromated Mo K radiation. Data collection (ω-scans) and processing (cell refinement, data reduction and Empirical/Numerical absorption correction) were performed using the CrystalClear program package.^65^ A crystal of compound **3a** (0.01 × 0.01 × 0.42 mm) was mounted on a Bruker D8 Quest Eco diffractometer. Measurements were conducted using graphite-monochromatic Mo-Kα radiation (λ = 0.71073 Å) and a Photon II detector at 23 °C. The structures were solved by direct methods using SHELXS v.2013/1 and refined by full-matrix least-squares techniques on F^2^ with SHELXL ver.2014/6.^66,67^ Hydrogen atoms were located by difference maps and were refined isotropically or were introduced at calculated positions as riding on bonded atoms. All non-hydrogen atoms were refined anisotropically. Plots of the structure were drawn using the Diamond 3 program package.^68^

The characterization of **1a** was based on a powder pattern recorded by a SMART LAB Rigaku diffractometer with Bragg–Brentano geometry, equipped with a pyrolytic graphite monochromator at diffracted beam position and using Cu Kα radiation (CuKα1 Å: 1.54060, Cu Kα2 Å: 1.54439). For the measurements standard aluminum holders with cavity were used. The power conditions were set at 40 kV/35 mA and the aperture as well as the antiscatter slit was set at 2/3°. The continuous step-scanning technique was used at steps of 0.02° with measuring time of 14 s/step and the recorded 2Θ range was from 2.0 to 70.0 °

**1a**: C_8_H_6_N_2_OS, C_2_H_6_OS, MW = 256.34 g/mol, orthorhombic, space group *P*2_1_2_1_2_1_, *a* = 4.7615(8) Å, *b* = 9.8297(19) Å, *c* = 26.787(6) Å, *V* = 1253.74 Å^3^, *Z* = 4.

**2a**: C_8_H_5_(Cl_0.54_,F_0.46_)­N_2_OS, MW = 205.08 g/mol, monoclinic, space group *P*2_1_/*c*, *a* = 11.3499(8) Å, *b* = 4.6851(3) Å, *c* = 16.3599(14) Å, α = 94.492(3)°, *V* = 867.3(1) Å^3^, *Z* = 4, *T* = 170 K, radiation Mo Kα, ρ­(calc) = 1.571 g cm^–3^, μ = 0.502 mm^–1^, reflections with *I* > 2σ­(*I*) = 1318, *R*_1_ = 0.0520, w*R*_2_^a^ = 0.1211, 2θ_max_ = 54°; reflections collected/unique/used, 7945/1871 [*R*_int_ = 0.0518]/1871; 131 parameters refined; (Δ/σ)_max_ = 0.001; (Δρ)_max_/(Δρ)_min_ = 0.789/–0.779 e/Å^3^; *R*1/w*R*2 (for all data), 0.0786/0.1360. The halogen site is shared by Cl an F atoms with occupancies 0.54(1) and 0.46(1) respectively, forming solid solution.

**3a**: C_8_H_5_FN_2_OS, C_2_H_6_OS, MW = 274.33 g/mol, monoclinic, space group *C*2/*c*, *a* = 25.888(3) Å, *b* = 4.7350(5) Å, *c* = 23.606(3) Å, α = 117.910(4)°, *V* = 2557.1(5) Å^3^, *Z* = 8, *T* = 296 K, radiation Mo Kα, ρ­(calc)= 1.425 g cm^–3^, μ = 0.420 mm^–1^, reflections with *I* > 2σ­(*I*) = 1627, *R*_1_ = 0.0898, w*R*_2_^a^ = 0.1483, 2θ_max_ = 50°; reflections collected/unique/used, 18,886/2220 [*R*_int_ = 0.1397]/ 2220; 159 parameters refined; (Δ/σ)_max_ = 0.000; (Δρ)_max_/(Δρ)_min_ = 0.298/–0.406 e/Å^3^; *R*1/w*R*2 (for all data), 0.1278/0.1599. The halogen site is shared by Cl an F atoms with occupancies 0.54(1) and 0.46(1) respectively, forming solid solution.

**4a**: C_8_H_5_ClN_2_OS, MW = 212.65 g/mol, monoclinic, space group *P*2_1_/*c*, *a* = 4.7421(4) Å, *b* = 16.1601(14) Å, *c* = 11.4921(9) Å, α = 94.621(2)°, *V* = 877.8(1)­Å^3^, *Z* = 4, *T* = 160 K, radiation Mo Kα, ρ­(calc) = 1.609 g cm^–3^, μ = 0.627 mm^–1^, reflections with *I* > 2σ­(*I*) = 1421, *R*_1_ = 0.0416, w*R*_2_^a^ = 0.0844. 2θ_max_ = 54°; reflections collected/unique/used, 10,250/1910 [*R*_int_ = 0.0295]/ 1910; 138 parameters refined; (Δ/σ)_max_= 0.000; (Δρ)_max_/(Δρ)_min_ = 0.318/–0.251 e/Å^3^; *R*1/w*R*2 (for all data), 0.0615/0.0960.

**5a**: C_8_H_5_ClN_2_OS, MW = 212.65 g/mol, monoclinic, space group *P*2_1_/*n*, *a* = 4.7297(5) Å, *b* = 15.1382(14) Å, *c* = 12.7060(13) Å, α = 97.354(4)°, *V* = 902.26(16) Å^3^, *Z* = 4, *T* = 160 K, radiation Mo Kα, ρ­(calc) = 1.566 g cm^–3^, μ = 0.610 mm^–1^, reflections with *I* > 2σ­(*I*) = 1078, *R*_1_ = 0.0576, w*R*_2_^a^ = 0.1082, 2θ_max_ = 54°; reflections collected/unique/used, 7908/1954 [*R*_int_ = 0.1083]/1954; 138 parameters refined; (Δ/σ)_max_ = 0.006; (Δρ)_max_/(Δρ)_min_ = 0.366/–0.389 e/Å^3^; *R*1/w*R*2 (for all data), 0.1214/0.1338.

**1**: C_62_H_50_AgN_2_OP_3_S, MW = 1071.88 g/mol, monoclinic, space group *P*2_1_/*n*, *a* = 11.7903(2) Å, *b* = 26.9548(5) Å, *c* = 16.4586(3) Å, α = 91.585(1)°, *V* = 5228.6(2) Å^3^, *Z* = 4, *T* = 170 K, radiation Mo Kα, ρ­(calc) = 1.362 g cm^–3^, μ = 0.561 mm^–1^, reflections with *I* > 2σ­(*I*) = 9415, *R*_1_ = 0.0315, w*R*_2_^a^ = 0.0699, 2θ_max_ = 54°; reflections collected/unique/used, 59,806/11,393 [*R*_int_ = 0.0423]/11,393; 831 parameters refined; (Δ/σ)_max_ = 0.002; (Δρ)_max_/(Δρ)_min_ = 0.379/–0.454 e/Å^3^; *R*1/w*R*2 (for all data), 0.0412/0.0728.

**2**: C_62_H_49_AgFN_2_OP_3_S, MW = 1089.87 g/mol, monoclinic, space group *P*2_1_/*c*, *a* = 14.1567(3) Å, *b* = 13.6903(3) Å, *c* = 27.3472(6) Å, α = 99.822(1)°, *V* = 5222.5(2) Å^3^, *Z* = 4, *T* = 160 K, radiation Mo Kα, ρ­(calc) = 1.386 g cm^–3^, μ = 0.566 mm^–1^,

Reflections with *I* > 2σ­(*I*) = 9415, *R*_1_ = 0.0315, w*R*_2_^a^ = 0.0699, 2θ_max_ = 54°; reflections collected/unique/used, 49,996/11,387 [*R*_int_ = 0.0581]/11,387; 640 parameters refined; (Δ/σ)_max_ = 0.001; (Δρ)_max_/(Δρ)_min_ = 1.333/–0.468 e/Å^3^; *R*1/w*R*2 (for all data), 0.0688/0.1156.

**3**: C_62_H_49_AgFN_2_OP_3_S, 2­(C_2_H_6_OS), MW = 1246.12 g/mol, triclinic, space group *P*1̅, *a* = 13.3904(3) Å, *b* = 14.4372(3) Å, *c* = 16.8200(4) Å, α = 108.073(1)°, β = 101.546(1)°, γ = 92.851(1)°, *V* = 3006.8(1) Å^3^, *Z* = 2, *T* = 160 K, radiation Mo Kα, ρ­(calc) = 1.376 g cm^–3^, μ = 0.570 mm^–1^, reflections with *I* > 2σ­(*I*) = 11,461, *R*_1_ = 0.0450, w*R*_2_^a^ = 0.1207, 2θ_max_ = 54°; reflections collected/unique/used, 55,868/13,108 [*R*_int_ = 0.0386]/13,108; 780 parameters refined; (Δ/σ)_max_ = 0.001; (Δρ)_max_/(Δρ)_min_ = 3.104/–0.506 e/Å^3^; *R*1/w*R*2 (for all data), 0.0508/0.1246; one ordered DMSO solvate molecule per cluster was refined, while an additional molecule was accounted for using the SQUEEZE procedure.

**4**: C_62_H_49_AgClN_2_OP_3_S, MW = 1106.32 g/mol, monoclinic, space group *P*2_1_/*c*, *a* = 14.0954(3) Å, *b* = 13.7480(3) Å, *c* = 27.2903(7) Å, α = 99.661(1) °, *V* = 5213.4(2) Å^3^, *Z* = 4, *T* = 160 K, radiation: Mo Kα, ρ­(calc) = 1.410 g cm^–3^, μ = 0.615 mm^–1^, reflections with *I* > 2σ­(*I*) = 8920, *R*_1_ = 0.0322, w*R*_2_^a^ = 0.0733, 2θ_max_ = 54°; reflections collected/unique/used, 62,891/11,123 [*R*_int_ = 0.0331]/11,123; 640 parameters refined; (Δ/σ)_max_ = 0.002; (Δρ)_max_/(Δρ)_min_ = 0.595/–0.494 e/Å^3^; *R*1/w*R*2 (for all data), 0.0439/0.0770.

**5**: C_62_H_49_AgClN_2_OP_3_S, 2­(C_2_H_6_OS), MW = 1262.57 g/mol, triclinic, space group *P*1̅, *a* = 13.4415(3) Å, *b* = 14.4916(3) Å, *c* = 16.9666(3) Å, α = 109.938(1)°, β = 100.322(1)°, γ = 92.903(1)°, *V* = 3034.1(1) Å^3^, *Z* = 2, *T* = 160 K, radiation Mo Kα, ρ­(calc) = 1.382 g cm^–3^, μ = 0.606 mm^–1^, reflections with *I* > 2σ­(*I*) = 11,366, *R*_1_ = 0.0335, w*R*_2_^a^ = 0.0798, 2θ_max_ = 54°; reflections collected/unique/used, 56,586/13,227 [*R*_int_ = 0.0295]/ 13,227; 914 parameters refined; (Δ/σ)_max_ = 0.003; (Δρ)_max_/(Δρ)_min_ = 0.789/–0.779 e/Å^3^; *R*1/w*R*2 (for all data), 0.0408/0.0825. One of the two DMSO solvate molecule was ordered, while the second was refined in two sites with 0.706(3) and 0.294(3) occupancies, respectively.

Crystallographic data (excluding structure factors) for the structures reported in this paper have been deposited with the Cambridge Crystallographic Data Centre as supporting publication no. CCDC- 2428860 (**2a**), 2429308 (**3a**), 2428863 (**4a**), 2428865 (**5a**), 2428858 (**1**), 2428859 (**2**), 2428861 (**3**), 2428862 (**4**) and 2428864 (**5**). Copies of the data can be obtained free of charge on application to CCDC, 12 Union Road, Cambridge CB2 1EZ, UK (fax: (+ 44) 1223–336–033; e-mail: deposit@ccdc.cam.ac.uk).

### Biological Tests

#### Solutions Used

Stock solutions of compounds **1a**–**5a** and **1**–**5** (0.01 M) in DMSO were freshly prepared and subsequently diluted with cell culture media to appropriate concentrations for various biological experiments. These included the assessment of cell viability using the SRB assay, micronucleus testing, cell morphology studies, cell cycle arrest, AO/EB staining, and mitochondrial membrane permeabilization tests. For CT-DNA binding studies, experiments were conducted in DMSO/buffer solutions. All biological experiments were performed in triplicate or more.

#### In Vitro Antiproliferative Activity

Stock solutions of **1a**–**5a** and **1**–**5** (0.01 M) were dissolved in DMSO and subsequently diluted with cell culture medium to the desired concentrations (0.5–30 μM). MCF-7, MDA MB-231, and MRC-5 cells were plated in 96-well flat-bottom microplates at various densities: 6000, 8000, and 2000 cells per well, respectively. The cells were incubated for 24 h at 37 °C and then exposed to **1a**–**5a** and **1**–**5** for 48 h.^24,28–30^

#### SRB Assay

Cytotoxicity was evaluated using the SRB colorimetric assay at λ = 568 nm, with cell survival percentages calculated relative to the untreated control cells. Detailed information on the SRB assay procedure can be found in the previous publication.^24,28–30^

#### In Vitro Micronucleus Assay

MRC-5 cells were seeded at a density of 2 × 10^4^ cells/well onto glass coverslips, which were placed in six-well plates containing 3 mL of cell culture medium and incubated for 24 h. The cells were then exposed to **1a–5a** and **1**–**5** at their respective IC_50_ values for 48 h. After exposure, the coverslips were washed three times with PBS and treated with a hypotonic solution (75 mM KCl) for 10 min at room temperature. The hypotonized cells were fixed with at least three changes of a 1/3 acetic acid/methanol solution. Following fixation, the coverslips were washed with cold methanol containing 1% acetic acid. Acridine orange staining (50 μg/mL) was applied for 15 min at 37 °C, after which the coverslips were rinsed three times with PBS to remove any excess stain. The number of micronucleated cells per 1000 cells was then counted.^24,28–30,44^

#### Cell Morphology Studies

MCF-7 cell morphology was observed under an inverted microscope after incubation with **1a**–**5a** and **1**–**5** for 48 h.

#### AO/EB Staining Detection of Apoptotic Cells

The MCF-7 cells were seeded into a 24-well plate (70,000 cells per well) and cultured at 37 °C for 24 h. Afterward, the cells were treated with **1a**–**5a** and **1**–**5** at their IC_50_ value for 48 h. Control and treated cells were collected after 48 h, the cells were harvested, washed twice with PBS and centrifuged. Twenty-five μL of cell suspension in PBS were incubated with 50 μg/mL AO and 50 μg/mL EB solution. Then, the cells were mixed gently and 10 μL of cells in suspension was placed on a glass slide and a coverslip was placed over it. At random, 300 cells were immediately observed in a fluorescent microscope (Optika inverted fluorescence microscope) and examined at ×60 magnification.^30,39^

#### Cell Cycle Arrest

The MCF-7 cells were seeded into a 24-well plate (70,000 cells per well) and cultured at 37 °C for 24 h. Afterward, the cells were treated with **1a**–**5a** and **1**–**5** at their IC_50_ value for 48 h. For each sample, 10,000 events were recorded. The resulting DNA histograms were drawn and quantified using the FlowJo software (version FlowJo X 10.0.7r2). This assay was performed as reported previously.^24,28–30^

#### Loss of the Mitochondrial Membrane Permeabilization (MPP)

The MMP assay was conducted using the ″Mitochondria Membrane Potential Kit for Microplate Readers″ (MAK147), purchased from Sigma-Aldrich. MCF-7 cells were treated with **1**–**5** at their IC_50_ concentration. Fluorescence intensity was measured at an excitation wavelength of λ_ex_ = 540 nm and an emission wavelength of λ_em_ = 590 nm. The experimental results included fluorescence intensities from the treated and untreated cell solutions.^24,28–30^

#### In Vivo Toxicity Evaluation and Uptake of **1**–**5** by Brine Shrimp 

Brine shrimp assay was performed as previously reported.^55^ Briefly, one gram of cysts was initially hydrated in freshwater for 1 h in a separating funnel or cone shaped container. Seawater was prepared by dissolving 17 g of sea salt in 500 mL of distilled water.^55^ The culture was facilitated with good aeration for 48 h at room temperature and under continuous illumination. After hatching, nauplii released from the eggshells were collected at the bright side of the cone (near the light source) by using micropipette. The larvae were isolated from the eggs by aliquoting them in small beaker containing NaCl 0.9%.^55^ An aliquot (0.1 mL) containing about 8–12 nauplii was introduced to each well of 24-well plate, and aliquots of **1**–**5** from stock solutions (0.01 M in DMSO,) were added in each well, at IC_50_^max^ (≈5 μM), as well as at concentrations up to five times higher than IC_50_^max^. The final volume of each well was 1 mL with NaCl 0.9%. Brine shrimps were observed after 24 h, using a stereoscope. Larvae were considered dead if they did not exhibit any internal or external movement in 10 s of observation. Each experiment was repeated three times.

### Ex Vivo Mechanism Studies

#### DNA Binding Studies

To prepare the DNA stock solution, CT-DNA was diluted in a buffer containing 15 mM trisodium citrate and 150 mM NaCl at pH 7, and stirred for several days (no longer than a week) at 4 °C.^24,26–30^ The absorbance of the DNA stock solution yielded a ratio of *A*_260_/*A*_280_ between 1.8 and 1.9, indicating that the DNA was sufficiently free from protein contamination.^24,26–30^ The concentration of CT-DNA was determined by measuring absorbance at 260 nm, using an extinction coefficient of 6600 M^–1^ cm^–1^.^24,26–30^

#### UV–Vis Spectroscopy Measurements

For the experiments of titration, UV spectra of CT-DNA in buffer solution in the absence and presence of **1**–**5** at *r* values of 0–0.13 (*r* = [complex]/[DNA], [DNA] = 10^–4^ M) were recorded.^24,26–30^ In order to determine the constant *K*_b_ value, UV spectra of **1**–**5** in the absence and presence of CT-DNA at *r* values 1–0.1 ([complex] = 10 μM, [CT DNA] = 10–100 μM) were investigated.^24,26–30^ The *K*_b_ value is obtained from the ratio of the slope to the *y*-intercept in plots [DNA]/(ε_a_–ε_f_) versus [DNA] (*e*_a_ = *A*_obsd_/[compound], *e*_f_ = the extinction coefficient for the free compound).^24,26–30^

#### Fluorescence Studies

The fluorescence spectroscopy method using EB was employed to determine the relative DNA binding properties of complexes **1**–**5** into CT-DNA. The emission spectra at 588 nm of EB (2.3 μM) solutions which contain CT-DNA (26 μM) in the absence or presence of various concentrations of complexes **1**–**5** (0–600 μM) were recorded upon their excitation at 527 nm.^24,28–30^ The apparent binding constant (*K*_app_) was calculated using the equation:

$$K_{\mathrm{EB}}^{*}\left[\mathrm{EB}\right]=K_{\mathrm{app}}^{*}\left[\mathrm{DNA binder}\right]$$

where [DNA binder] is the concentration of the complex at a 50% reduction of the fluorescence, *K*_EB_= 10^7^ M^–1^, and [EB] = 2.3 μM.^24,28–30^

The concentration of the drug required to achieve a 50% reduction in fluorescence is determined from the plot of *I*_o_/*I* versus the concentration of the complex [DNA binder],

$$\frac{I_{o}}{I}=1+K_{\mathrm{SV}}^{*}\left[\mathrm{DNA binder}\right]$$

where *I*_o_ and *I* represent the fluorescence intensities of CT-DNA in the absence and presence of complexes **1–5**, respectively. *K*_SV_ is the Stern–Volmer dynamic quenching constant and [DNA binder] is the total concentration of the quencher (complexes **1**–**5**).^24,28–30^

#### Viscosity Measurements

This study was carried out as previously reported.^30^ The kinematic viscosity of DNA solutions with or without **1**–**5** (0–0.40 [**1**–**5**]/[DNA] molar ratios) was measured with a rotational viscometer.

#### Study of the Inhibitory Activity **1**–**5** toward the Peroxidation of Linoleic Acid by the Enzyme LOX

This study performed as previously reported.^30,31,61^ In brief, a 0.2 M borate buffer solution at pH 9 was prepared by dissolving boric acid in double-distilled water (ddw). The pH was then adjusted to 9.0 using a 50% w/w sodium hydroxide solution.

The linoleic acid substrate solution was prepared as follows: 0.05 mL of linoleic acid was dissolved in 0.05 cm^3^ of 95% ethanol within a volumetric flask. Subsequently, double-distilled water was gradually added until reaching a final volume of 5 cm^3^. This solution was then diluted with 30 cm^3^ of borate buffer.

The LOX enzyme solution was prepared in an ice-cold bath by diluting a stock solution of 10,000 U/mL. For each reaction, 500 U of enzyme was used per 3 cm^3^ of reaction mixture. One unit of LOX activity corresponds to an increase in absorbance at 234 nm of 0.001 per minute.

Enzyme activity was monitored by UV spectrophotometry. Specifically, 0.05 cm^3^ of enzyme solution was added to a cuvette containing 2 cm^3^ of linoleic acid solution, along with the appropriate volumes of buffer and inhibitor solutions **1**–**5** (concentration range of 1.5 to 12.5 μΜ). The enzymatic activity was determined by measuring the increase in absorbance at 234 nm, which corresponds to the oxidation of linoleic acid (ε = 25,000 M^–1^ cm^–1^). During the assays, the substrate concentration was maintained constant at 0.3 mM, while the buffer and inhibitor volumes were adjusted to achieve final inhibitor concentrations ranging from 2 to 30 μM.

The degree of LOX activity (*A*%) in the presence or absence of the **1**–**5** was calculated according to the following equation:^30,31,61^

$$A\%=\left(\frac{V_{o}\;(\mathrm{in}\;\mathrm{the}\;\mathrm{presence}\;\mathrm{of}\;\mathrm{inhibitor}}{V_{o}\;\left(\mathrm{without}\;\mathrm{inhibitor}\right)}\right)\times 100\left(\%\right)$$

where *V*_o_ is the initial rate.

The value of the initial rate (*V*_o_, μΜ min^–1^) was calculated according to the following formula:

$$V_{o}=\frac{\Delta C}{\Delta t}=\frac{\Delta A}{\varepsilon \times \Delta t}=\frac{\mathrm{tg}\alpha }{\varepsilon }$$

where *C* is the concentration of the product (hydroperoxy-linoleic acid), *t* is the reaction time, *ε* is the molar extinction coefficient of hydroperoxy-linoleic acid, and tgα represents the slope of the kinetic curve obtained by plotting absorbance against time.

#### Calculation of **1**–**5** Lipophilicity

The lipophilicity of compounds **1**–**5** was calculated using MarvinSketch software version 24.1.2 (ChemAxon, 2024 release). The crystallographic *.mol files, obtained from the 3D structures of the compounds derived from single-crystal X-ray diffraction data were directly imported into the software for lipophilicity calculations. Lipophilicity was estimated as the log *P* value, representing the logarithm of the partition coefficient between *n*-octanol and water. The software employs fragment-based methods to predict log *P*, considering the contributions of various atomic and structural fragments to the overall lipophilicity. All calculations were performed under default settings without additional geometry optimization, to maintain consistency with the experimentally determined molecular structures.

## Supplementary Material



## Notes

Detailed analysis of vibrational spectroscopy and ^1^H NMR spectroscopy; tables of selected bond lengths and angles of the complexes and their ligands; biological parameters of compounds and other heteroleptic Ag­(I) complexes; Regression statistics summary output; powder XRD pattern of **1a**; IR and ^1^H NMR spectra of complexes and ligands; UV spectra of complexes; UV spectra of CT-DNA with complexes, emission spectrum of CT-DNA-EB complex with the complexes (PDF)
