# Supplementary material for: Silver(I) Ions Bridging Oxadiazole-2-thione Derivatives with Mitochondriotropic Agents: A Promising Strategy for Potent Anticancer Chemotherapy
Source: Inorg Chem. 2025 Jun 24;64(26):13010–29. doi: 10.1021/acs.inorgchem.5c01095 (PMC12239085; doi:10.1021/acs.inorgchem.5c01095)
Supplement: Supplementary file 1 [file ic5c01095_si_001.pdf]

Supporting Information

Silver(I) ions Bridging Oxadiazole-2-Thione Derivatives with Mitochondriotropic Agents: A Promising Strategy for Potent Anticancer Chemotherapy

Christina N. Banti[a,*] George Vagenas[a], Catherine P. Raptopoulou[b], Vassilis Psycharis[b,*], John C. Plakatouras[a] Sotiris K. Hadjikakou[a,c,*]

[a] Biological Inorganic Chemistry laboratory, Department of Chemistry, University of Ioannina, 45110 Ioannina, Greece;
[b] Institute of Nanoscience and Nanotechnology, NCSR "Demokritos", 15310, Athens Greece
[c] Institute of Materials Science and Computing, University Research Center of Ioannina (URCI), 45110 Ioannina, Greece

*All correspondence should be addressed to:
Dr. C.N. Banti (Adjunct Lecturer); email: cbanti@uoi.gr
Dr. V Psycharis (Researcher); email: v.psycharis@inn.demokritos.gr
Dr. S.K. Hadjikakou (Professor); e-mail: shadjika@uoi.gr; tel. x30-26510-08374, x30-26510-08362

Vibrational spectroscopy: The vibrational band observed at 3200-3300 cm^{-1} in the ATR-FTIR spectrum of X-PODTH (3225.0 cm^{-1} for H-PODTH, 3225.0 cm^{-1} for o-F-PODTH, 3234.0 cm^{-1} for p-F-PODTH, 3276.0 cm^{-1} for o-Cl-PODTH, and 3273.5 cm^{-1} for m-Cl-PODTH) is attributed to the ν(N-H) bond. This band is absent in the spectra of compounds **1-5**, indicating that the silver(I) ion is coordinated through this nitrogen atom [34-35] (Figures S2-S6). The tautomeric equilibrium between oxadiazole-2-thione and oxadiazole-2-thiole (Scheme 1) is known to favor the thionate form in the solid state and thiolate in solution. This equilibrium gives rise to the acidic behavior of the labile thioamide hydrogen atom. This hydrogen is deprotonated in the presence of potassium hydroxide (Scheme 3), leading to the formation of the anionic species of the thionate/thiolate form, which subsequently coordinates covalently to the silver cation. Thus, in the case of the uncoordinated ligands the position of this labile hydrogen differs between the solid and solution states. In the solid state, it is bonded with the nitrogen atom, whereas in solution, it resides on the sulfur atom. Since both the XRD and FT-IR measurements were performed in the solid state the presence of H in the N atom of thioamide group is confirmed. Consequently, the absence of N–H stretching vibrations serves as evidence for the deprotonation of the thioamide group, confirming its coordination to the silver cation.

The band at 1499-1506 cm^{-1} in the spectra of X-PODTH (1506 for H-PODTH, 1499 for o-F-PODTH, 1506 for p-F-PODTH, 1505 for o-Cl-PODTH and 1501 cm^{-1} for m-Cl-PODTH) is assigned to the thioamide I band (δ(N–H) + ν(C-N)). These are slightly shifted in the case of the **1-5** at 1509 (**1**), 1494 (**2**), 1498 (**3**), 1483 (**4**) and 1510 cm^{-1} (**5**), respectively [34-35] (Figures S2-S6).

The band at 1276-1282 cm^{-1} in the spectra of X-PODTH (1276 for H-PODTH, 1284 for o-F-PODTH, 1282 for p-F-PODTH, 1280 for o-Cl-PODTH and 1282 cm^{-1} for m-Cl-PODTH) are assigned to the thioamide II (ν(C–N) + δ(N–H) + ν(C=S)) band, which mainly consisted as ν(ring), bond vibrations [34-35,39]. These bands are absent in the compounds **1-5** suggesting coordination of the ligands through the >C-S bond (Figures S2-S6).

The band at 962-968 cm^{-1} in the spectra of X-PODTH (965 for H-PODTH, 962 for o-F-PODTH, 962 for p-F-PODTH, 963 for o-Cl-PODTH and 968 cm^{-1} for m-Cl-PODTH) are assigned to the thioamide III band (ν(C–N) + ν(C–S)). These bands undergo strong shift in the case of **1-5** at 951 (**1**), 957 (**2**), 949 (**3**), 961 (**4**) and 966 cm^{-1} (**5**), respectively confirming the coordination though the >C-S sulfur [34-35,39] (Figures S2-S6).

The thioamide IV (ν(C–S) *band* is observed at 685-692 cm^{-1} in the spectra of X-PODTH (692 for H-PODTH, 687 for o-F-PODTH, 685 for p-F-PODTH, 689 for o-Cl-PODTH and 690 cm^{-1} for m-Cl-PODTH) and these bands are shifted in the case of **1-5** at 690 (**1**), 690 (**2**), 690 (**3**), 684 (**4**) and 673 cm^{-1} (**5**), respectively [34-35,39] (Figures S2-S6).

The vibration band of C-O-C is observed 1069-1080 cm^{-1} in the spectra of X-PODTH (1080 for H-PODTH, 1074 for o-F-PODTH, 1072 for p-F-PODTH, 1071 for o-Cl-PODTH and 1069 cm^{-1} for m-Cl-PODTH) and these bands are shifted in the case of **1-5** at 1092 (**1**), 1070 (**2**), 1091 (**3**), 1069 (**4**) and 1062 cm^{-1} (**5**), respectively [34-35] (Figures S2-S6).

Finally, the vibrational bands at 511.3 and 488.3 cm^{-1} are attributed to the ν_{sym}(C–P) vibrations of uncoordinated triphenylphosphine (TPP). These bands are shifted to 502–487 (**1**), 502–486 (**2**), 499–491 (**3**), 502–471 (**4**), and 504–488 (**5**) cm^{-1} in the IR spectra of complexes **1–5**, confirming the coordination of TPP on the metal center (Figures S2–S6). Thus, while the presence of a band at 504–488 cm^{-1} alone indicates the presence of TPP, it does not confirm its coordination. It is specifically the shift of the ν_{sym}(C–P) band between the complexes and uncoordinated TPP that confirms coordination in the case of these complexes

^{1}H NMR spectroscopy: The signals at 7.41-7.39 ppm (m) in the ^{1}H-NMR spectrum of TPP are attributed to the m- and *p*-H[Ph-], while those at 7.27-7.20 ppm (m) correspond to the *o*-H[Ph-]. Moreover, the resonance signals in the ^{1}H-NMR spectra of the oxadiazole-2-thone ligands appears at: 7.90-7.88 ppm (d) $H^{2',6'}$[Ph-] and 7.67-7.58 ppm (m) $H^{3',4',5'}$[Ph-], (**H-PODTH**); 7.93-7.89 ppm (m) $H^{6'}$[o-F-Ph-] and 7.72-7.41 ppm (m) $H^{3',4',5'}$[o-F-Ph-], (***o*-F-PODTH**); 7.98-7.93 ppm (m) $H^{2',6'}$[*p*-F-Ph-] and 7.46-7.42 ppm (m) $H^{3',5'}$[*p*-F-Ph-] (***p*-F-PODTH**); 7.93-7.91 ppm (m) $H^{6'}$[*o*-Cl-Ph-] and 7.72-7.55 ppm (m) $H^{3',4',5'}$[*o*-Cl-Ph-],(***o*-Cl-PODTH**); 7.87-7.84 ppm (m) $H^{2',6'}$[*m*-Cl-Ph-] and 7.73-7.61 ppm (m) $H^{4',5'}$[*m*-Cl-Ph-])***m*-Cl-PODTH)** (Figures S7-S11) [40].

These resonance signals are observed in the ^{1}H-NMR spectra of thee complexes at : 7.47-7.39 ppm (m) $H^{2',6'}$[Ph-] and at 7.33-7.24 ppm (m) $H^{3',4',5'}$[Ph-] and *p-,m-,o*-H[Ph-] of TPP.(**1**); 7.65-7.39 ppm (m) $H^{6'}$[o-F-Ph-] and 7.33-7.23 ppm (m) $H^{3',4',5'}$[o-F-Ph-] and *p-,m-,o*-H[Ph-] of TPP. (**2**); 7.51-7.40 ppm (m) $H^{2',6'}$[*p*-F-Ph-] and at 7.35-7.25 ppm (m) $H^{3',5'}$[*p*-F-Ph-] and *p-,m-,o*-H[Ph-] of TPP (**3**); 7.57-7.42 ppm (m) $H^{6'}$[*o*-Cl-Ph-] and 7.39-7.32 ppm (m) $H^{3',4',5'}$[*o*-Cl-Ph-] and *p-,m-,o*-H[Ph-] of TPP. (**4**); 7.47-7.36 ppm $H^{2',6'}$[*m*-Cl-Ph-] and 7.33-7.24 ppm (m) $H^{4',5'}$[*m*-Cl-Ph-] and *p-,m-,o*-H[Ph-] of TPP, (**5**) (Figures S7-S11). The strong shift that these signals undergo in respect to their corresponding ones of the ligands suggest the formation of the complexes in solution.

Table S1. Selected bond lengths (Å) and angles (°)of the ligands studied

1a*			2a			3a			4a			5a	
**bond distances**													
**S2-C3**	1.622		**S1-C8**	1.638(3)		**S1-C1**	1.647(6)		**S1-C1**	1.644(2)		**S1-C8**	1.648(5)
**C3-N2**	1.298		**C8-N2**	1.333(4)		**C1-N1**	1.321(6)		**C1-N1**	1.325(3)		**C8-N2**	1.335(5)
**N2-H**	1.106		**N2-H**	0.84(3)		**N1-H**	0.88(7)		**N1-H**	0.83(3)		**N2-H**	0.86(5)
**N2-N1**	1.397		**N1-N2**	1.381(4)		**N1-N2**	1.378(7)		**N1-N2**	1.377(3)		**N1-N2**	1.387(5)
**N1-C4**	1.263		**N1-C7**	1.295(4)		**N2-C2**	1.289(6)		**N2-C2**	1.286(3)		**N1-C7**	1.292(5)
**C4-O2**	1.326		**O1-C7**	1.372(4)		**C2-O1**	1.378(6)		**O1-C2**	1.374(3)		**O1-C7**	1.377(5)
**O2-C3**	1.400		**O1-C8**	1.381(4)		**O1-C1**	1.369(6)		**O1-C1**	1.374(3)		**O1-C8**	1.374(5)
**C4-C5**	1.479		**C7-C6**	1.454(4)		**C2-C3**	1.448(7)		**C2-C3**	1.460(3)		**C5-C7**	1.459(6)
**bond angles**													
**S2-C3-N2**	133.47		**S1-C8-N2**	130.9(3)		**S1-C1-N1**	132.0(4)		**S1-C1-N1**	130.5(2)		**S1-C8-N2**	131.9(3)
**N2-C3-O2**	102.73		**N2-C8-O1**	104.0(2)		**N1-C1-O1**	104.4(5)		**N1-C1-O1**	104.2(2)		**N2-C8-O1**	104.4(4)
**S2-C3-O2**	123.78		**S1-C8-O1**	125.1(2)		**S1-C1-O1**	123.6(3)		**S1-C1-O1**	125.28(17)		**S1-C8-O1**	123.7(3)
**torsion angles**													
**N1-C4-C5-C6**	6.01		**N1-C7-C6-C1**	8.6(5)		**N2-C2-C3-C4**	2.5(9)		**N2-C2-C3-C8**	2.2(4)		**C4-C5-C7-N1**	-4.3(7)
**Hydrogen bonds / Inramolecular Interactions**													
**N2[H]···O1**	2.741		**N2[H]···N1**	2.925(3)		**N2[H]···O2**	2.740(6)		**N1[H]--N2**	2.852(3)		**S1···N2[H]**	3.281(4)
**<N2-H···O1**	163.89		**N2-H···N1**	155(3)		**<N2-H···N1**	171(8)		**<N1-H···N2**	134(3)		**S1···H-N2**	175(5)
**S1···O1 '**	3.262								**N1[H]—Cl1**	3.474(2)(3)			
**<O1'···S1-O1**	166.52								**<N1-H···Cl1**	153(3)			

***** as predicted by the preliminary structural model of collected low quality data

Table S2. Selected bond distances and angles of **1-5**

**1**			**2**			**3**			**4**			**5**	
**bond distances**													
**Ag1-S1**	2.6119(5)		**Ag1-S1**	2.6262(10)		**Ag1-S1**	2.5722(9)		**Ag1-S1**	2.6186(8)		**Ag1-S1**	2.5810(7)
**Ag1-P1**	2.5699(7)		**Ag1-P1**	2.5418(9)		**Ag1-P1**	2.5444(7)		**Ag1-P1**	2.5407(6)		**Ag1-P1**	2.6111(6)
**Ag1-P2**	2.5599(7)		**Ag1-P2**	2.5502(8)		**Ag1-P2**	2.5671(8)		**Ag1-P2**	2.5493(6)		**Ag1-P2**	2.5620(7)
**Ag1-P3**	2.5329(6)		**Ag1-P3**	2.5247(9)		**Ag1-P3**	2.5863(8)		**Ag1-P3**	2.5202(7)		**Ag1-P3**	2.5377(6)
**S1-C1**	1.709(2)		**S1-C1**	1.692(3)		**S1-C1**	1.711(3)		**S1-C1**	1.698(2)		**S1-C55**	1.711(2)
**N1-C1**	1.297(3)		**C2-C1**	1.304(5)		**N1-C1**	1.303(5)		**N1-C2**	1.284(3)		**N1-C55**	1.298(3)
**N1-N2**	1.412(3)		**N1-N2**	1.415(6)		**N1-N2**	1.409(5)		**N1-N1**	1.414(3)		**N1-N2**	1.416(3)
			**F1-C8**	1.660(4)		**F1-C6**	1.384(9)		**Cl1-C8**	1.740(2)		**Cl1-C59**	1.751(3)
**bond angles**													
**S1-Ag1-P1**	110.48(2)		**S1-Ag1-P1**	113.91(4)		**S1-Ag1-P1**	110.25(3)		**S1-Ag1-P1**	113.44(3)		**S1-Ag1-P1**	101.13(2)
**S1-Ag1-P2**	97.63(2)		**S1-Ag1-P2**	86.62(3)		**S1-Ag1-P2**	101.26(3)		**S1-Ag1-P2**	86.47(2)		**S1-Ag1-P2**	101.02(2)
**S1-Ag1-P3**	112.42(2)		**S1-Ag1-P3**	111.35(3)		**S1-Ag1-P3**	105.57(3)		**S1-Ag1-P3**	112.10(2)		**S1-Ag1-P3**	114.20(2)
**P1-Ag1-P2**	107.27(2)		**P1-Ag1-P2**	112.28(3)		**P1-Ag1-P2**	114.61(3)		**P1-Ag1-P2**	112.80(2)		**P1-Ag1-P2**	109.91(2)
**P1-Ag1-P3**	112.39(2)		**P1-Ag1-P3**	111.86(3)		**P1-Ag1-P3**	113.75(3)		**P1-Ag1-P3**	111.94(2)		**P1-Ag1-P3**	113.37(2)
**P2-Ag1-P3**	115.67(2)		**P2-Ag1-P3**	118.61(3)		**P2-Ag1-P3**	110.29(3)		**P2-Ag1-P3**	117.81(2)		**P2-Ag1-P3**	115.65(2)
**torsion angle**													
**N2-C2 -C3 -C4**	9.5(4)		**N1-C2-C3-C8**	-34.8(7)		**N2-C2-C3-C4**	-11.8(5)		**N1-C2-C3-C8**	-35.1(4)		**N2-C56-C57-C58**	19.9(3)

Table S3. Ag-S-C angles and the smallest dihedral angles of **1-5**

compound	Ag1-S1-C1 (°)	Dihedral angle (°)
**1**	109.15(7)	P3-Ag1-S1-C1: 59.72(8)
**2**	116.7(1)	P3-Ag1-S1-C1: 51.7(1)
**3**	108.1(1)	P1-Ag1-S1-C1: 43.7(1)
**4**	117.54(7)	P3-Ag1-S1-C1: 50.39(9)
**5**	106.88(8)	P3-Ag1-S1-C1:-35.91(9)

Table S4. The biological parameters of **1-5** and other heteroleptic Ag(I) complexes of triphenylphosphine and NSAID

	pIC_{50} MCF-7 [1]	pIC_{50} LOX [1]	Log(MW)	LogP	$Log(K_b)$	Ref
**1**	5.7	5.6	3.0	17.9	5.0	*
**2**	5.6	5.1	3.0	18.1	4.8	*
**3**	5.7	5.2	3.0	18.1	5.1	*
**4**	5.5	5.1	3.0	18.5	4.9	*
**5**	5.8	5.7	3.0	18.5	5.0	*
$Ag(mef)(TPP)_2$	5.7	5.1	2.9	13.7	5.1	30
$\{[Ag(asp)(TPP)_3](dmf)\}$	5.6	5.1	3.1	16.4	5	26
$[Ag(dicl)(TPP)_2]$	5.6	>4.5	3	12.9	4.9	29
$\{[Ag(napr)(TPP)_3](H_2O)\}$	6.2	5.3	3.1	17.7	5.5	32
$[Ag(nim)(TPP)_2]$	6	4.9	3	11.7	5.3	28
$[Ag(pHbza)(TPP)_2]$	5.9	5.1	2.9	10.2	5.4	26
$[Ag(salH)(TPP)_2]$	5.6	5.6	2.9	10.2	5.1	26

* in this study; [1] $p(IC_{50})$= $-Log(IC_{50})$; TPP= triphenylphosphine; mefH= mefenamic acid; asp= aspirin; dicl= diclofenac, napr= naproxen; nim= nimesulide; pHbzaH= p- hydroxybenzoic acid; salH= salicylic acid

Table S5. Regression statistics summary output

Regression Statistics								
**Multiple R**	0.9411							
**R Square**	0.8857							
**Adjusted R Square**	0.8095							
**Standard Error**	0.0894							
**Observations**	11.0000							
**ANOVA**								
	df	*SS*	*MS*	*F*	*Significance F*			
**Regression**	4.0000	0.3711	0.0928	11.6200	0.0055			
**Residual**	6.0000	0.0479	0.0080					
**Total**	10.0000	0.4191						
	Coefficients	*Standard Error*	*t Stat*	*P-value*	*Lower 95%*	*Upper 95%*	*Lower 95.0%*	*Upper 95.0%*
**Intercept**	-0.1110	1.9969	-0.0556	0.9575	-4.9973	4.7752	-4.9973	4.7752
**log(MW)**	0.2385	0.7020	0.3397	0.7457	-1.4793	1.9563	-1.4793	1.9563
**logP**	0.0150	0.0173	0.8640	0.4208	-0.0275	0.0574	-0.0275	0.0574
**log(Kb)**	0.9826	0.1682	5.8432	0.0011	0.5711	1.3940	0.5711	1.3940
**pLOX**	-0.0197	0.1133	-0.1739	0.8677	-0.2970	0.2576	-0.2970	0.2576

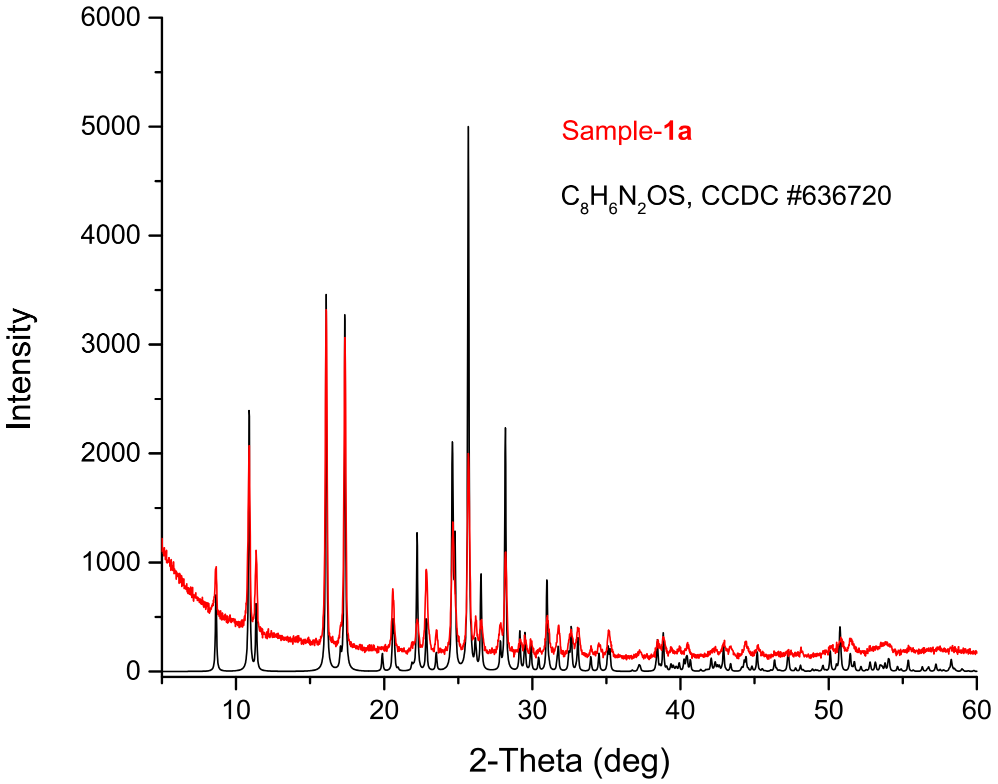

Figure S1. Powder XRD pattern (red line) recorded from a sample of compound-**1a** as compared to a simulated pattern of compound $C_8H_6N_2OS$ which has been calculated using the structural model given in the cif file with CCDC #636720 [36].

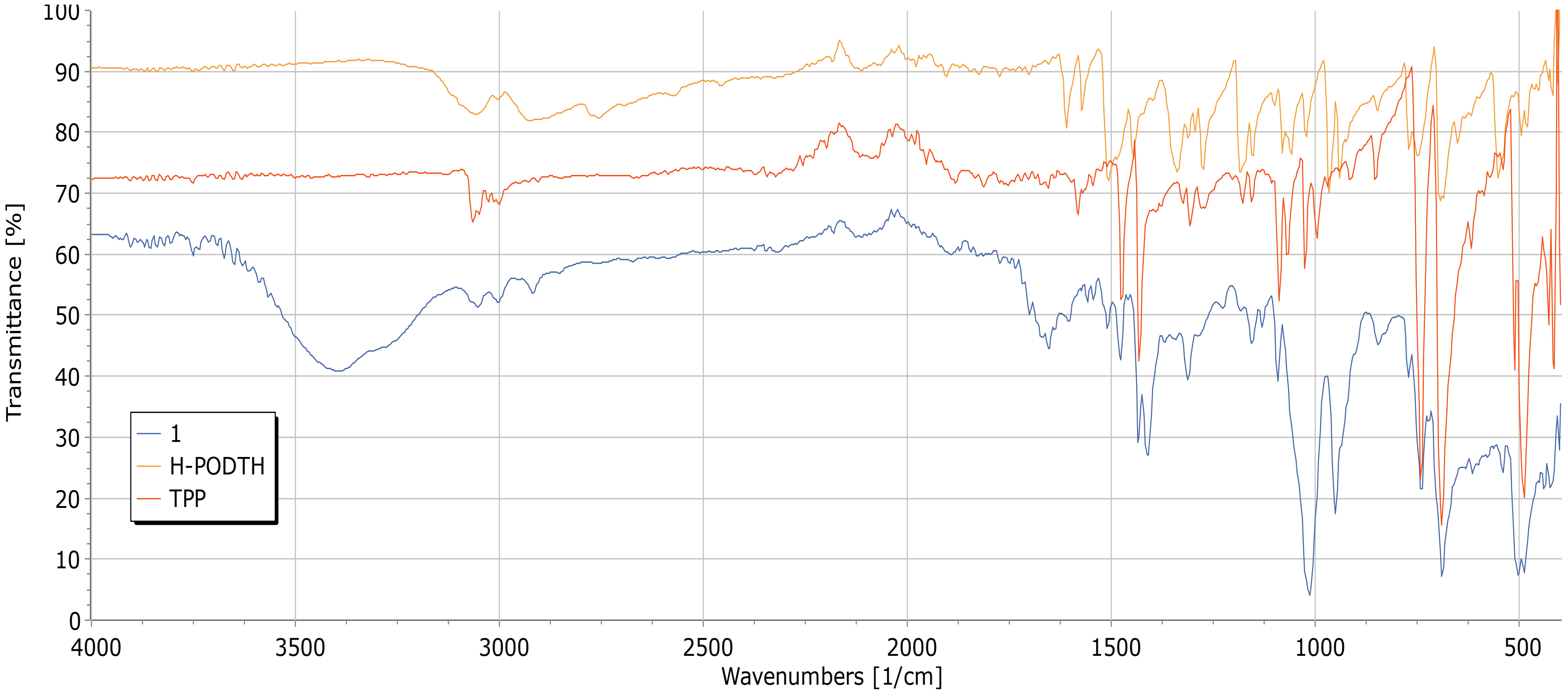

Figure S2. IR spectra of **1**, H-PODTH and TPP.

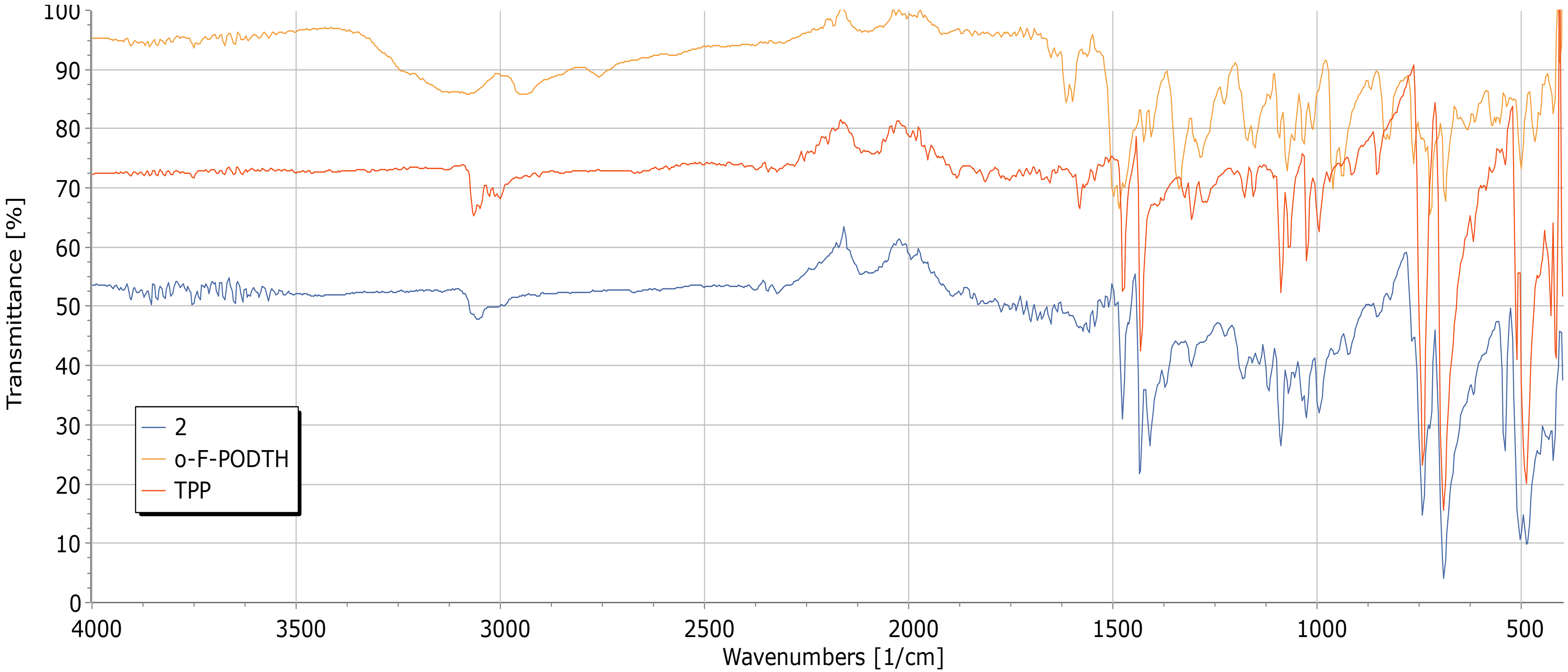

Figure S3. IR spectra of **2**, o-F-PODTH and TPP.

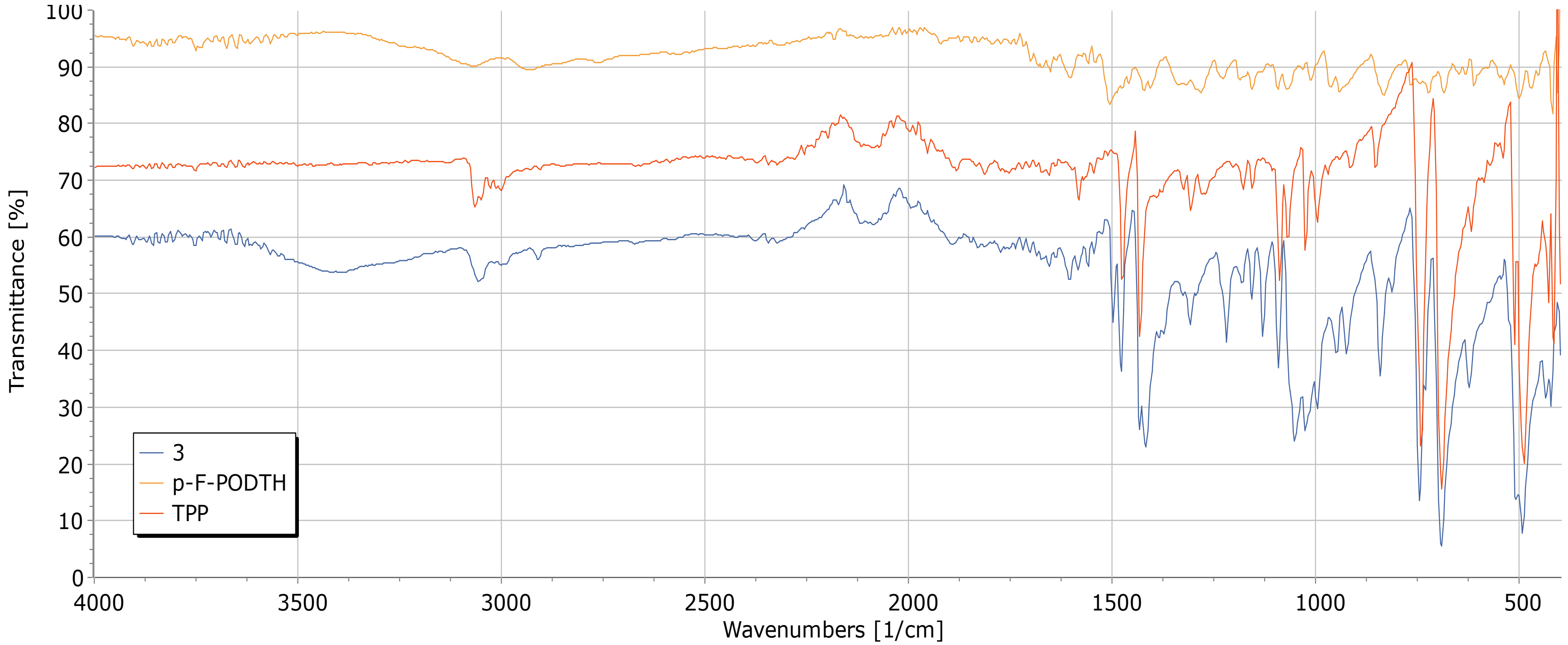

Figure S4. IR spectra of **3**, p-F-PODTH and TPP.

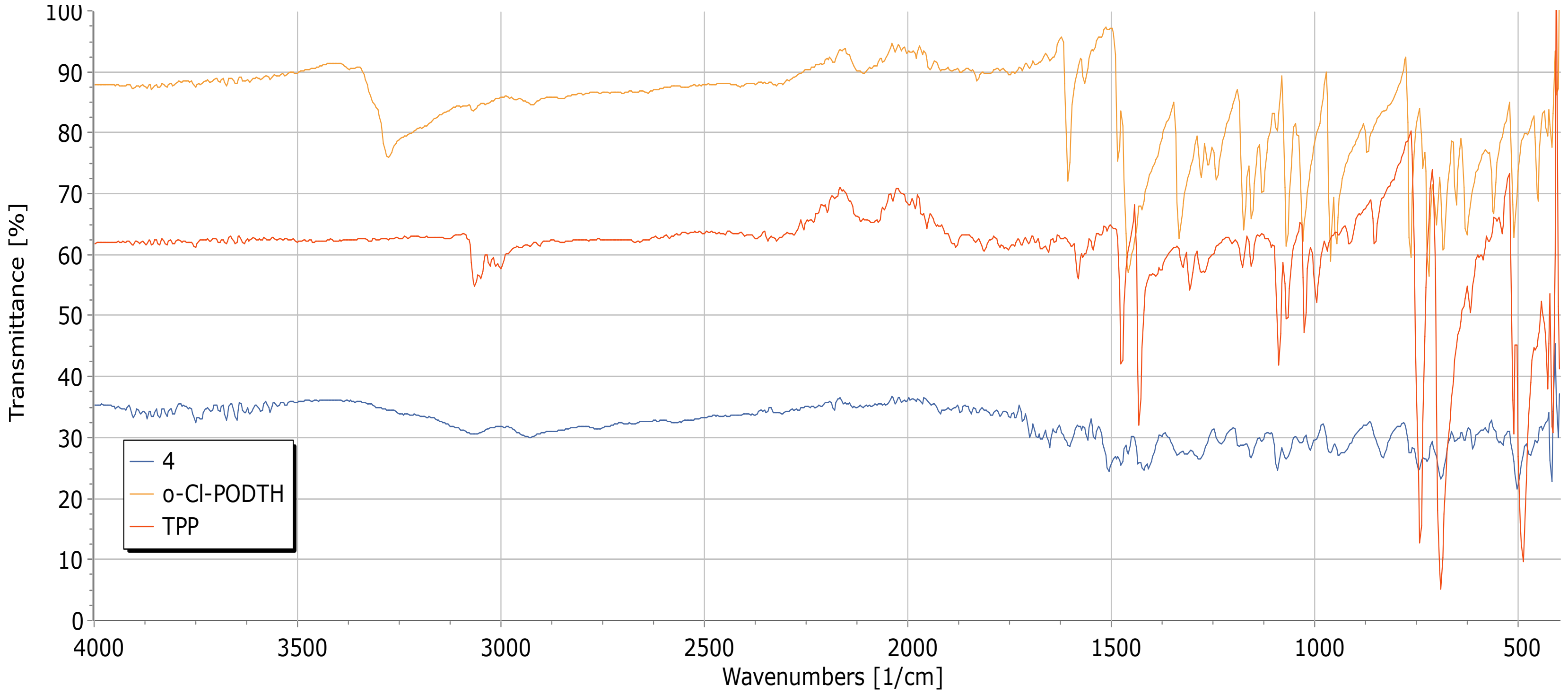

Figure S5. IR spectra of **4**, o-Cl-PODTH and TPP.

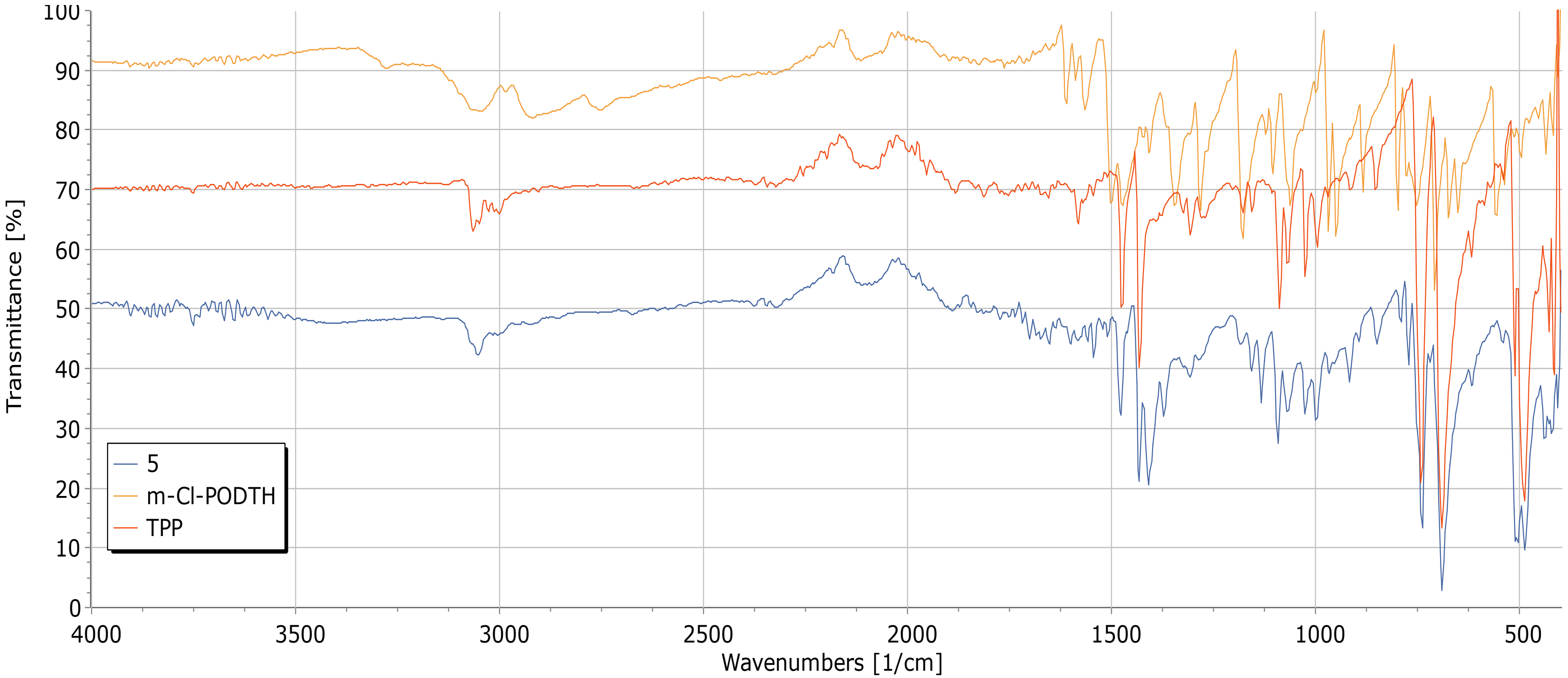

Figure S6. IR spectra of **5**, m-Cl-PODTH and TPP.

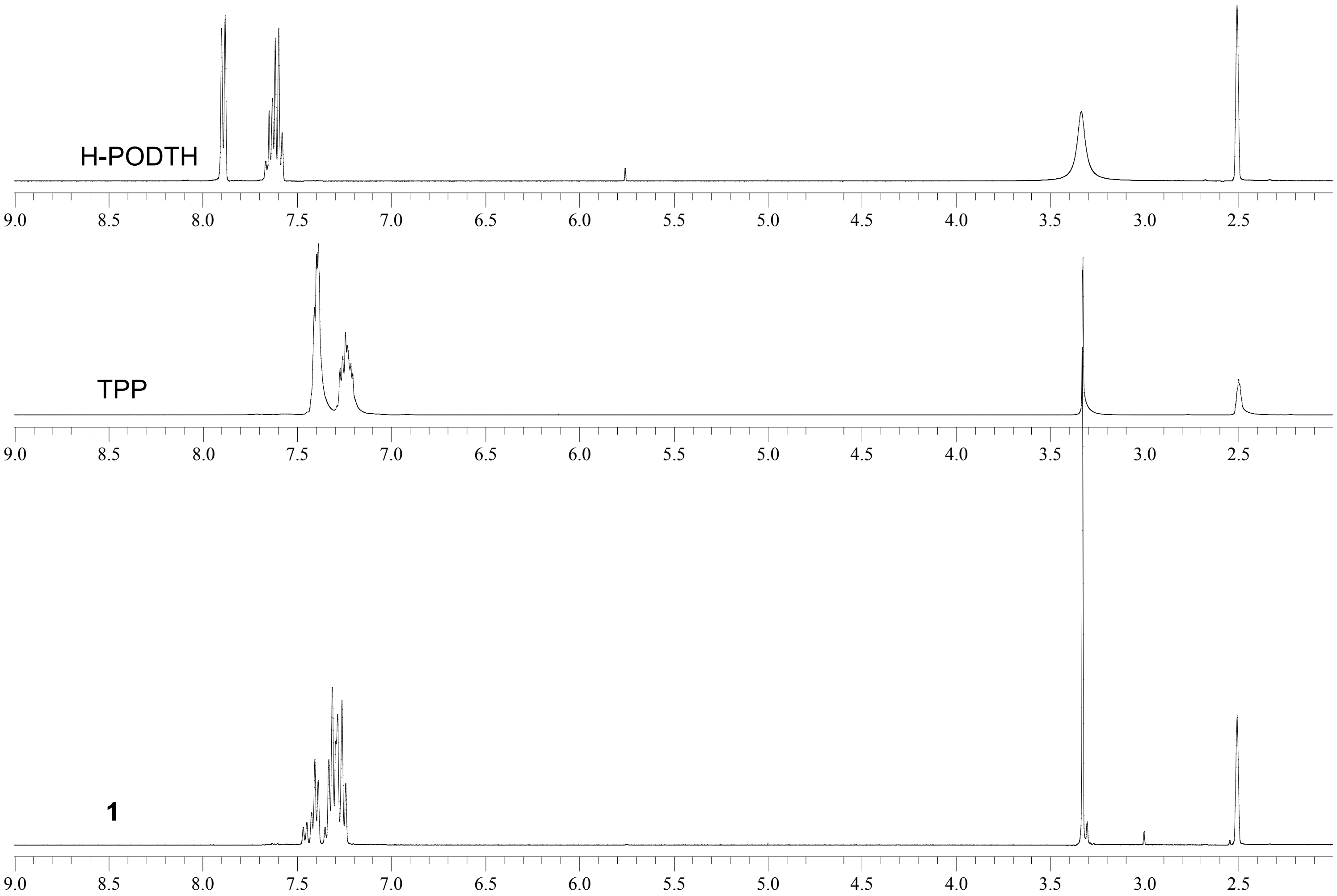

Figure S7. ^{1}H-NMR spectra of H-PODTH, TPP and **1** in dmso-d_6

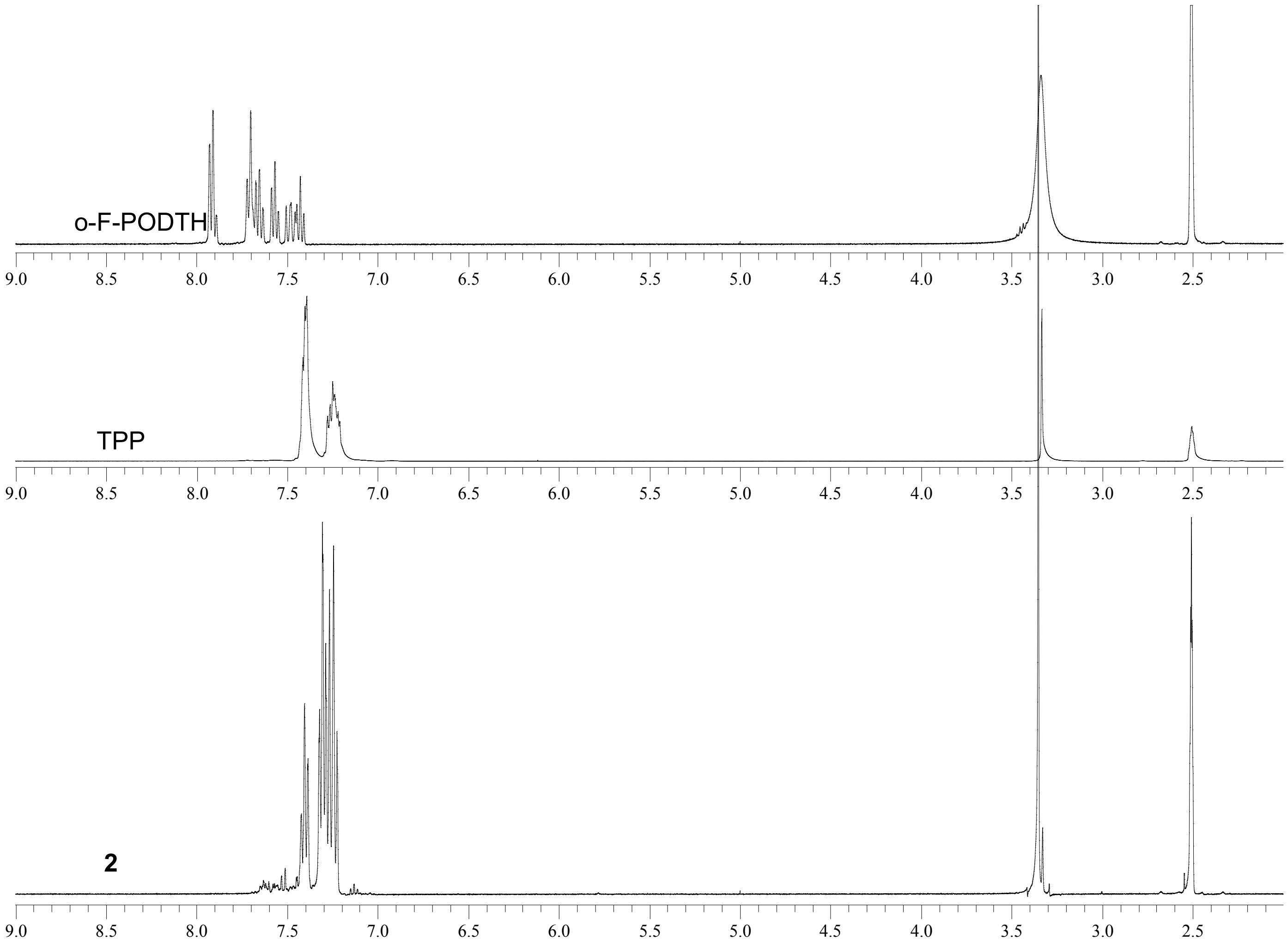

Figure S8. ^{1}H-NMR spectra of o-F-PODTH, TPP and **2** in dmso-d_6

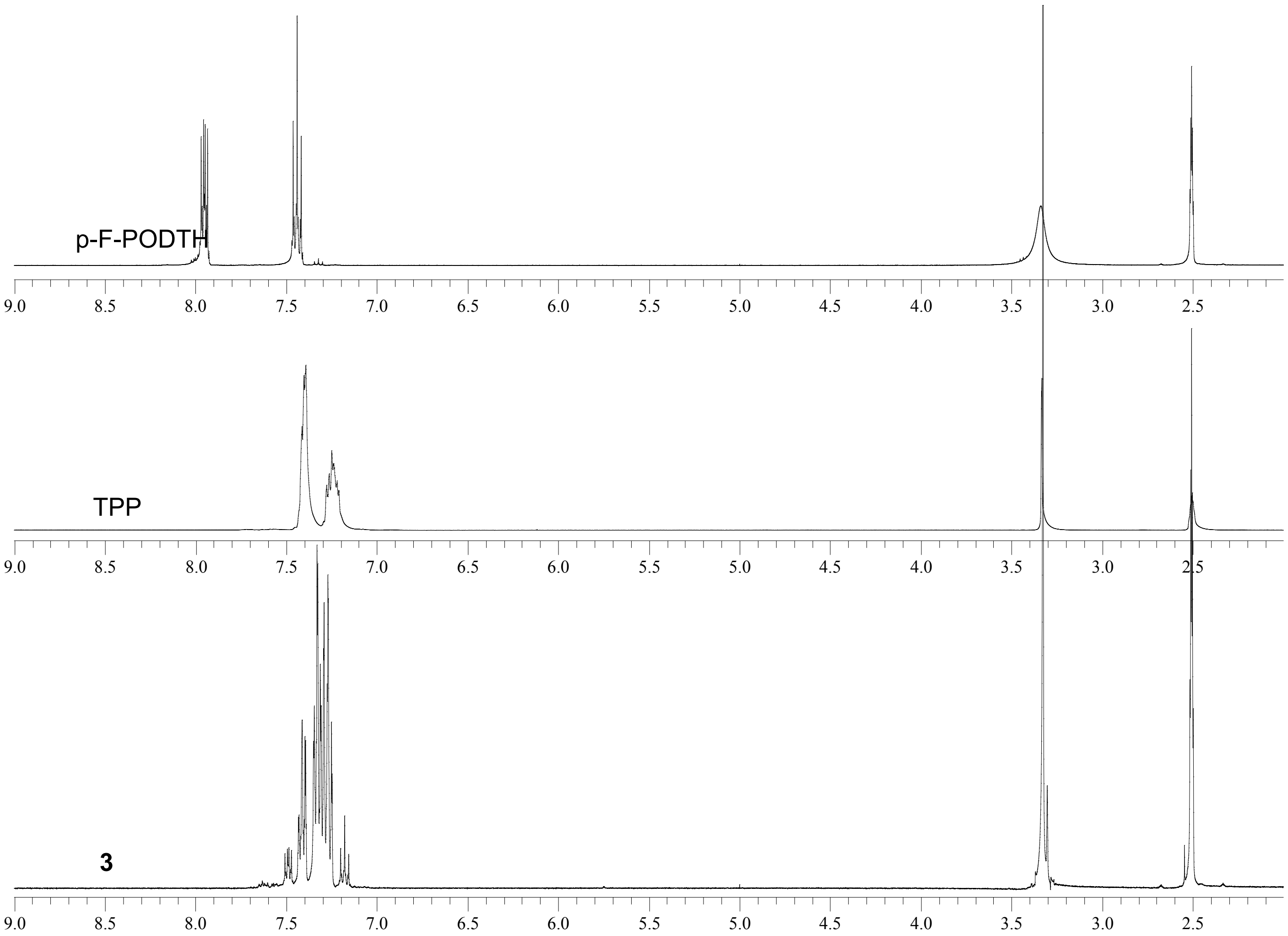

Figure S9. ^{1}H-NMR spectra of p-F-PODTH, TPP and **3** in dmso-d_6

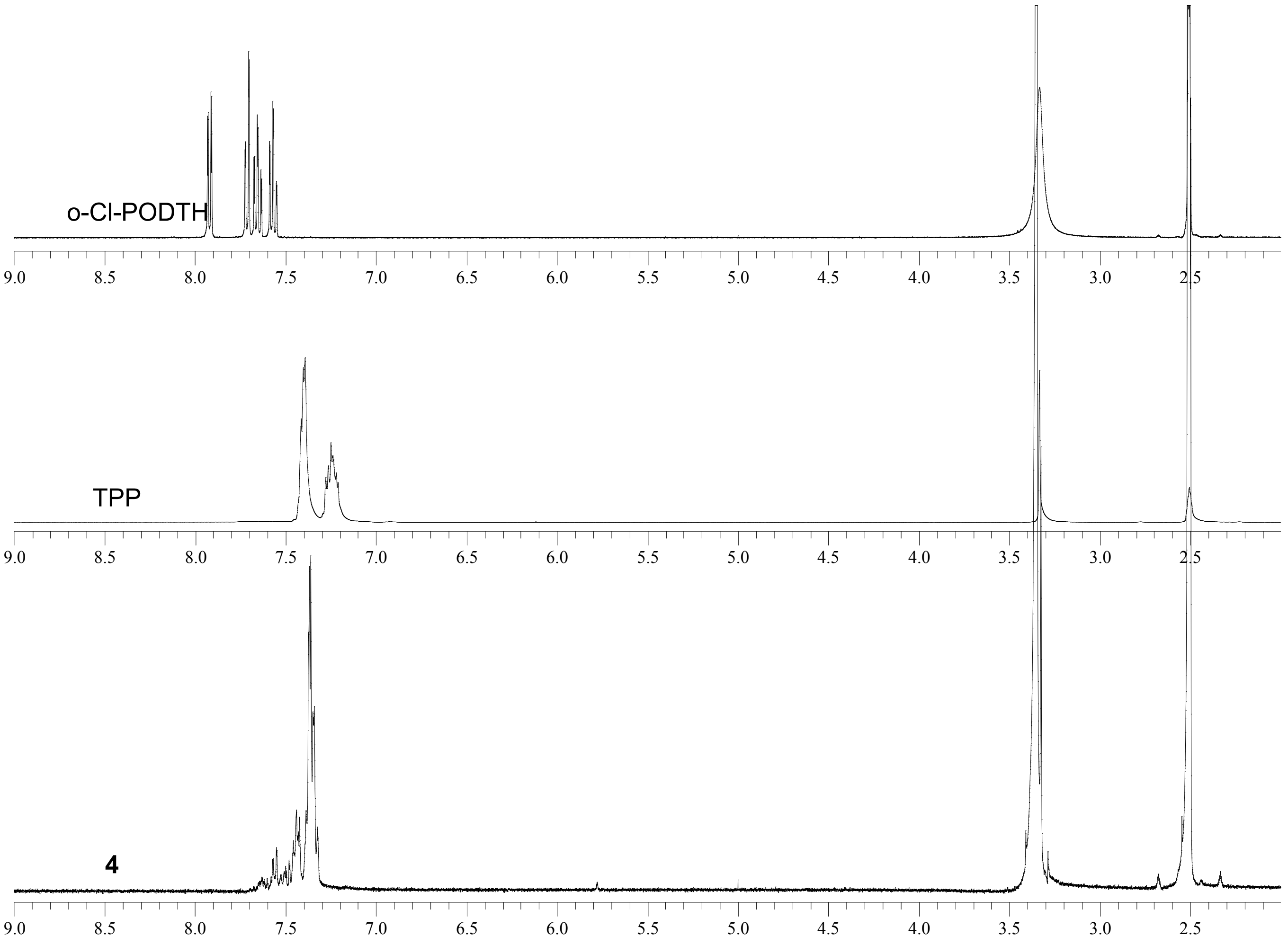

Figure S10. ^{1}H-NMR spectra of o-Cl-PODTH, TPP and **4** in dmso-d_6

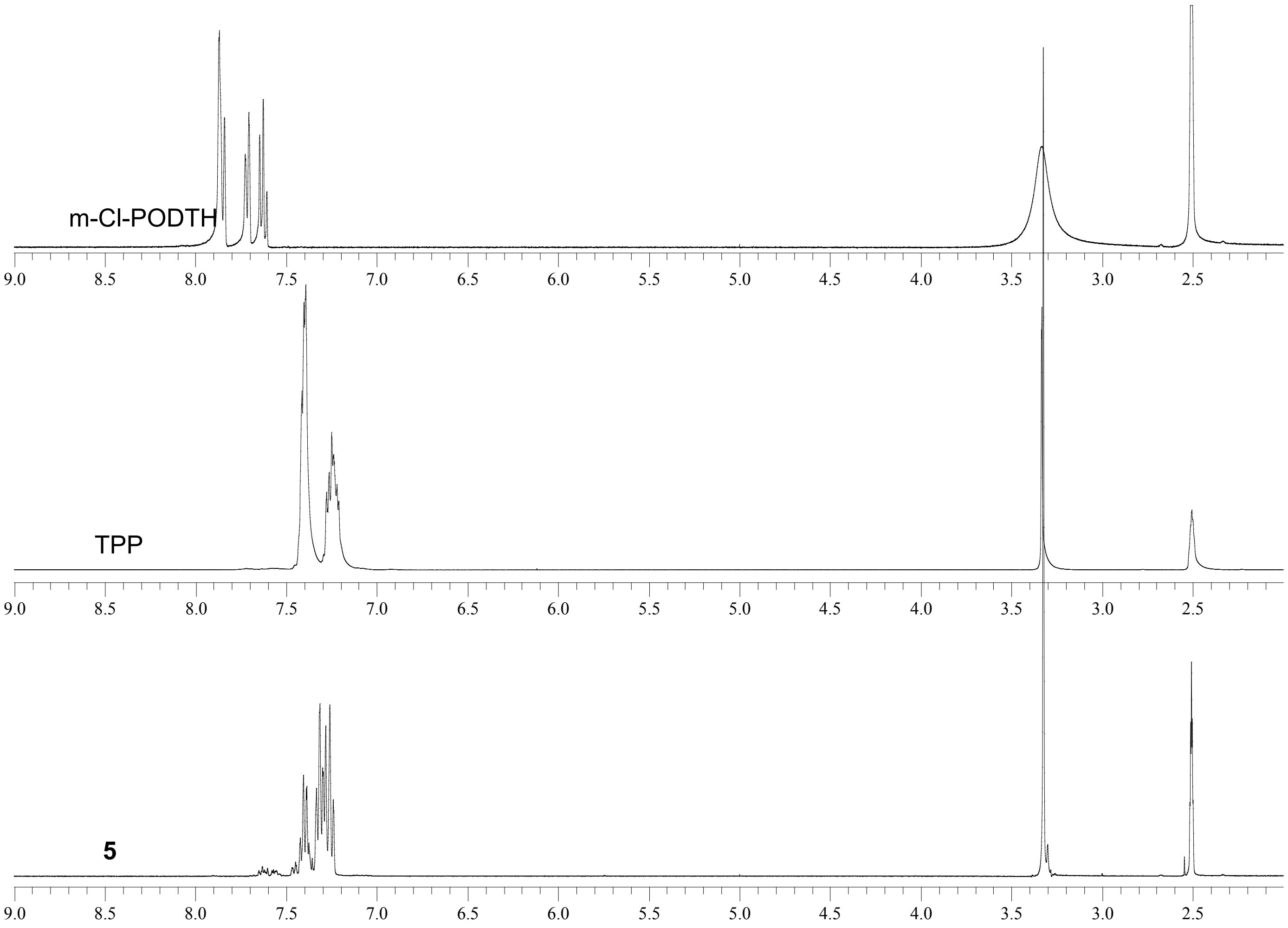

Figure S11. ^{1}H-NMR spectra of m-Cl-PODTH, TPP and **5** in dmso-d_6

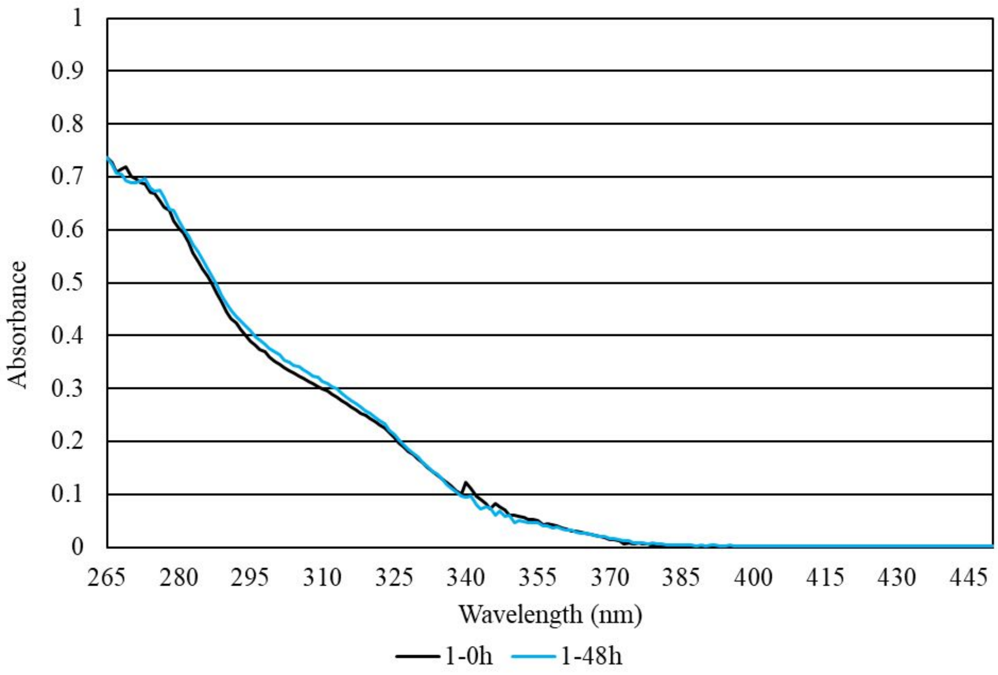

Figure S12. UV spectra of **1** (25 µM) in DMSO in 0 and after 48 hr.

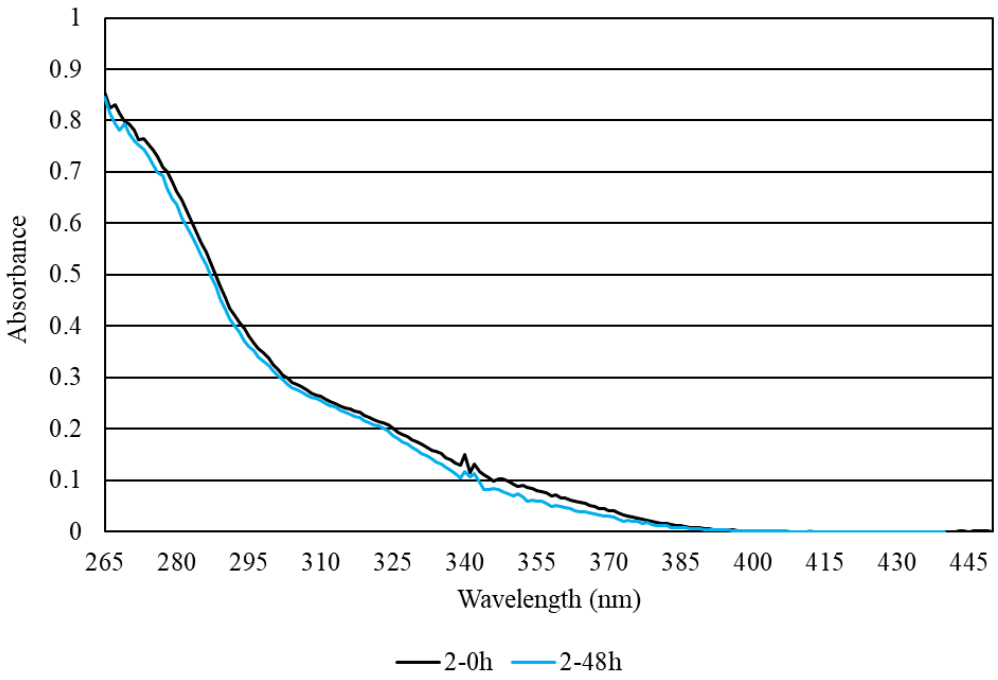

Figure S13. UV spectra of **2** (25 µM) in DMSO in 0 and after 48 hr.

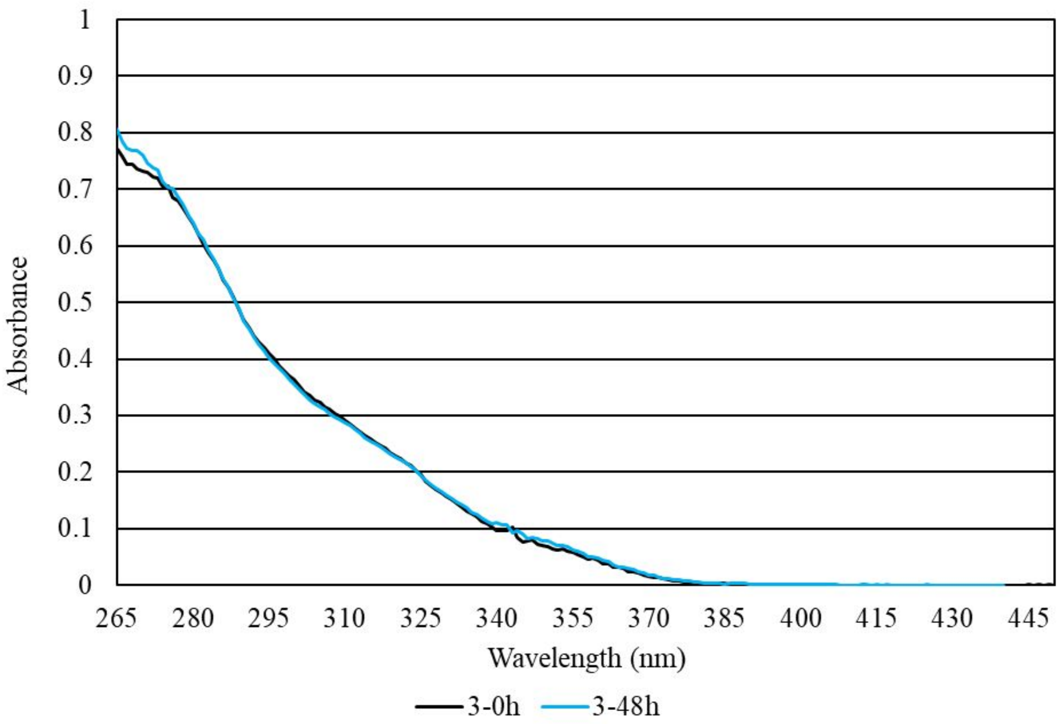

Figure S14. UV spectra of **3** (25 µM) in DMSO in 0 and after 48 hr.

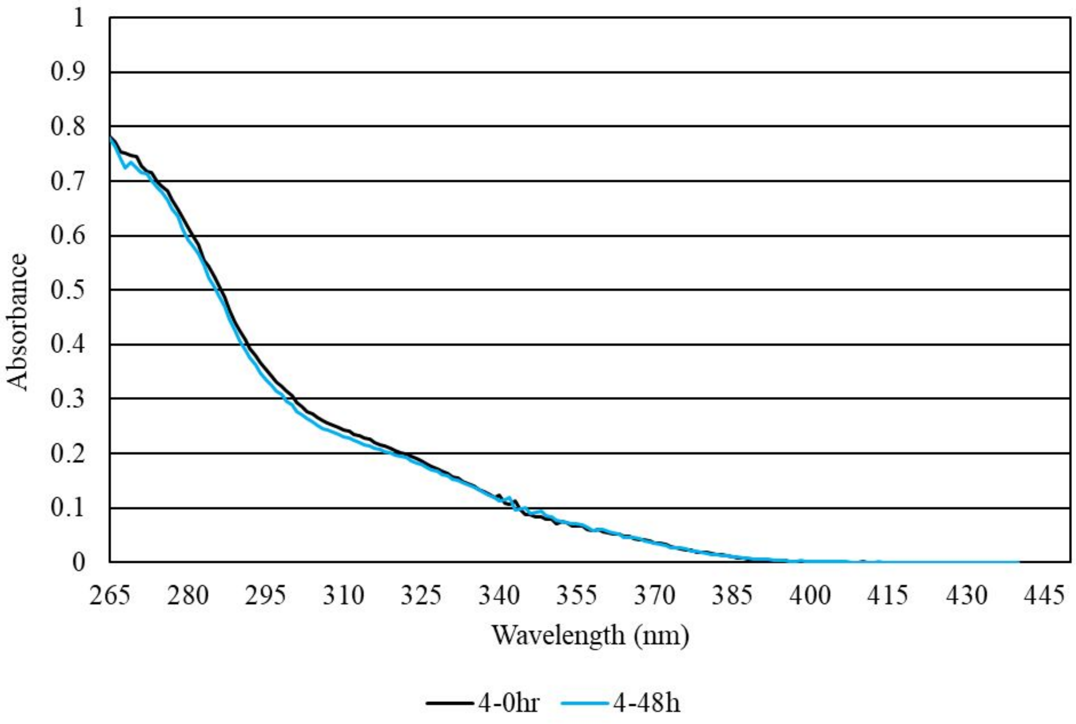

Figure S15. UV spectra of **4** (25 µM) in DMSO in 0 and after 48 hr.

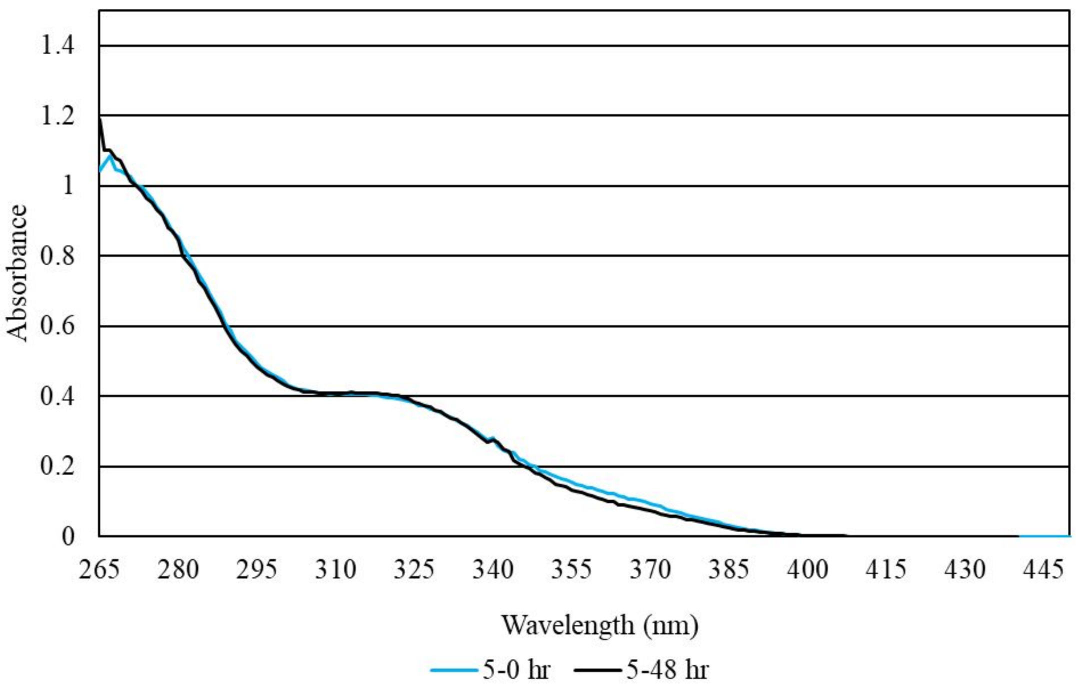

Figure S16. UV spectra of **5** (25 μM) in DMSO in 0 and after 48 hr.

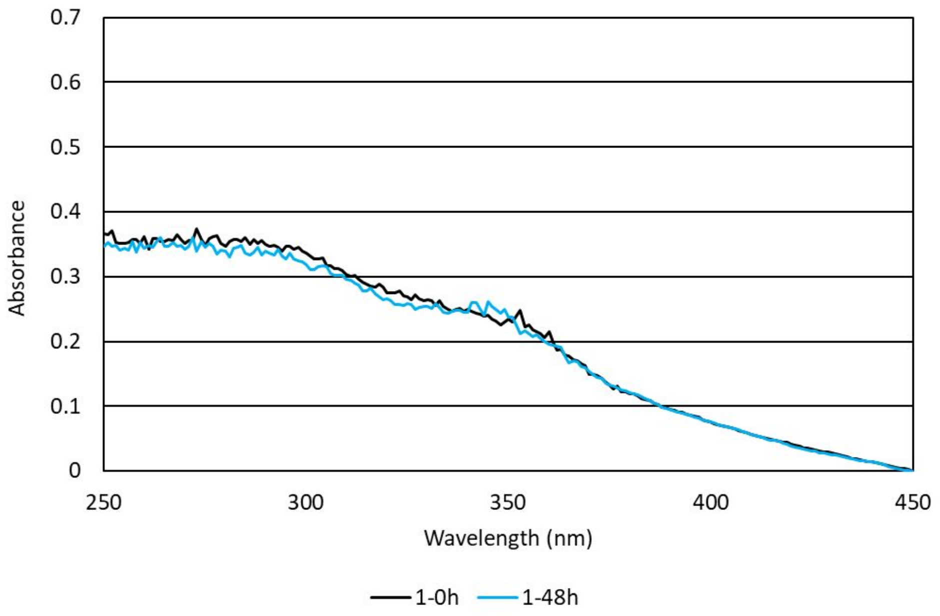

Figure S17. UV spectra of **1** (40 μM) in PBS/DMSO (250/1 v/v) in 0 and after 48 hr.

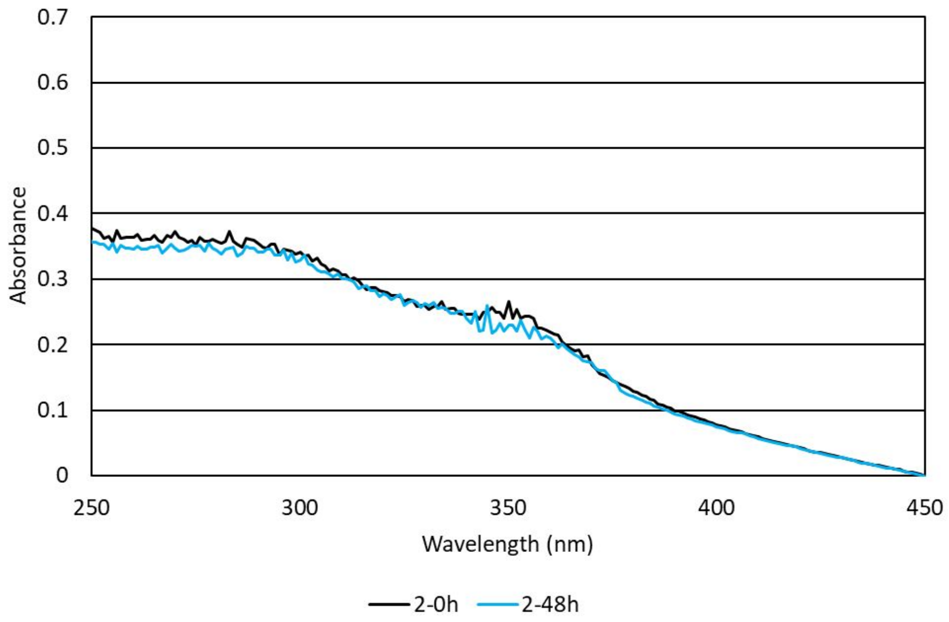

Figure S18. UV spectra of **2** (40 µM) in PBS/DMSO (250/1 v/v) in 0 and after 48 hr.

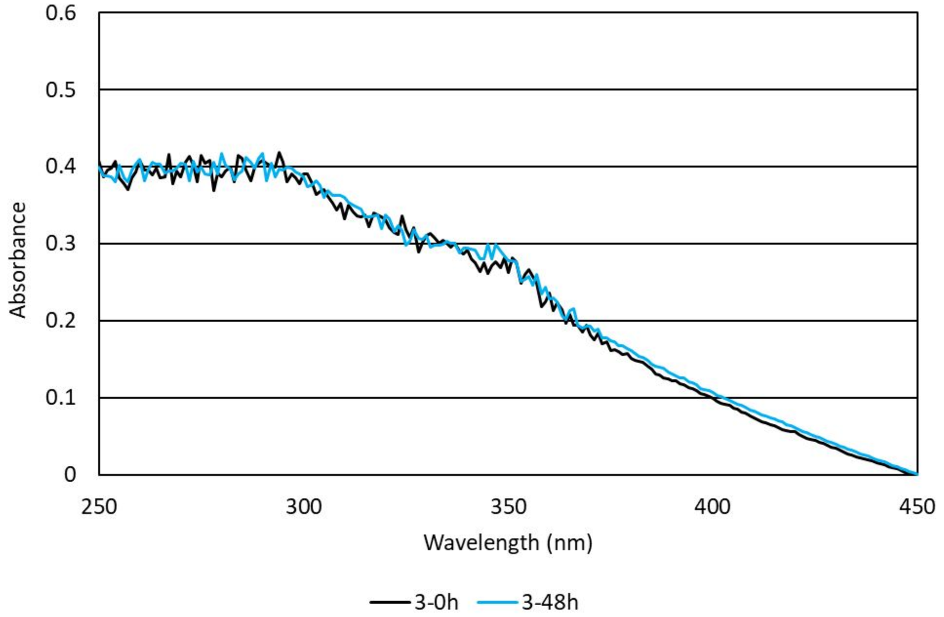

Figure S19. UV spectra of **3** (50 µM) in PBS/DMSO (200/1 v/v) in 0 and after 48 hr.

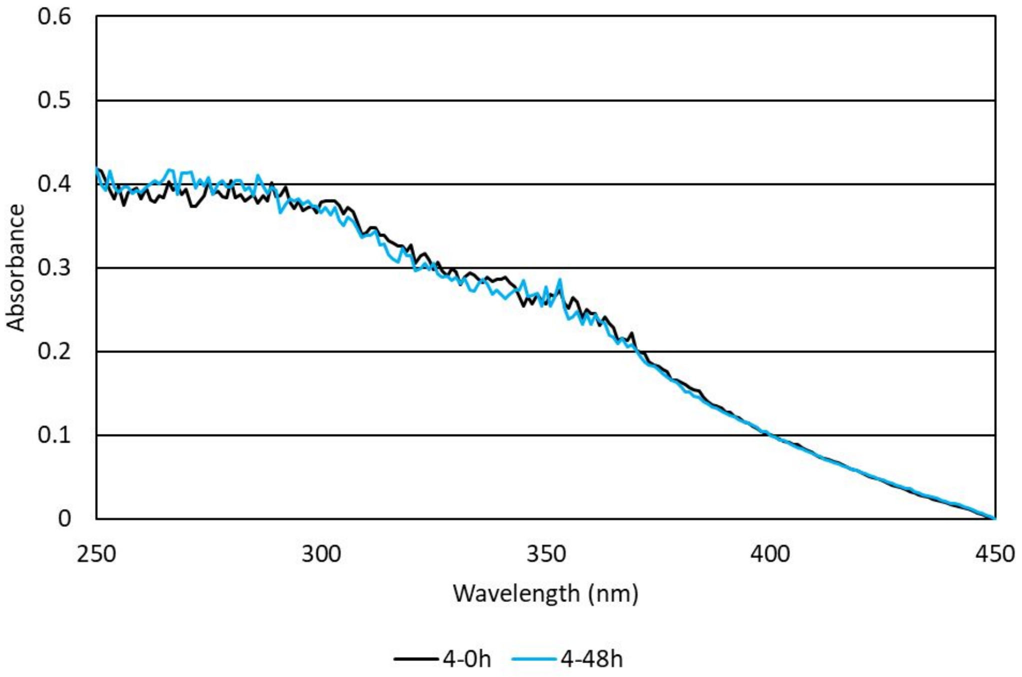

Figure S20. UV spectra of **4** (50 µM) in PBS/DMSO (200/1 v/v) in 0 and after 48 hr.

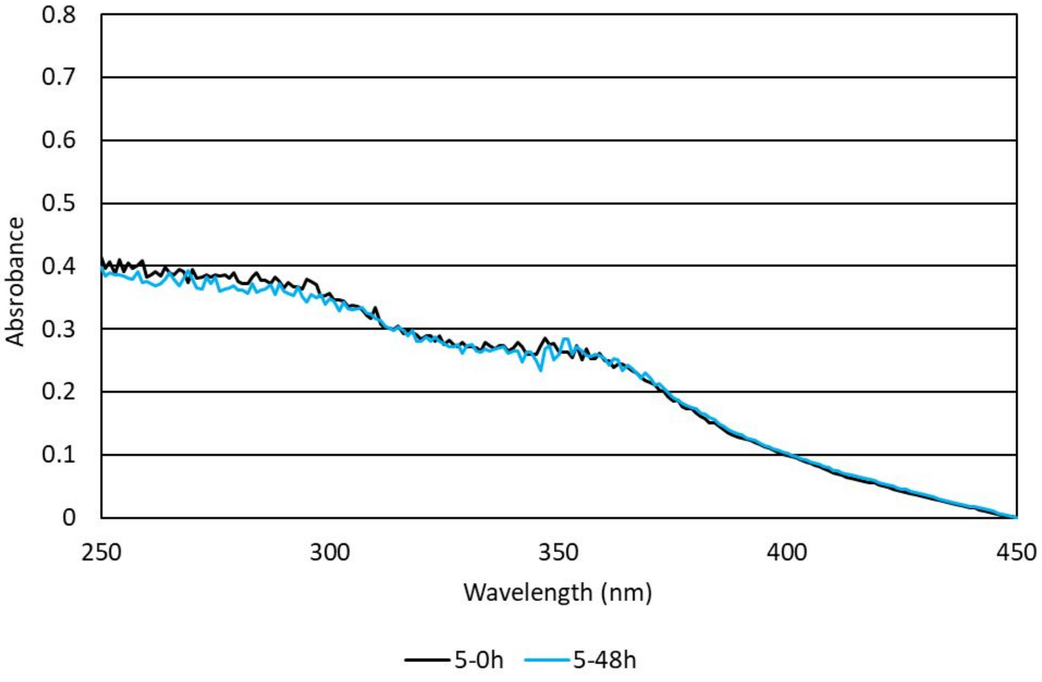

Figure S21. UV spectra of **5** (50 µM) in PBS/DMSO (200/1 v/v) in 0 and after 48 hr.

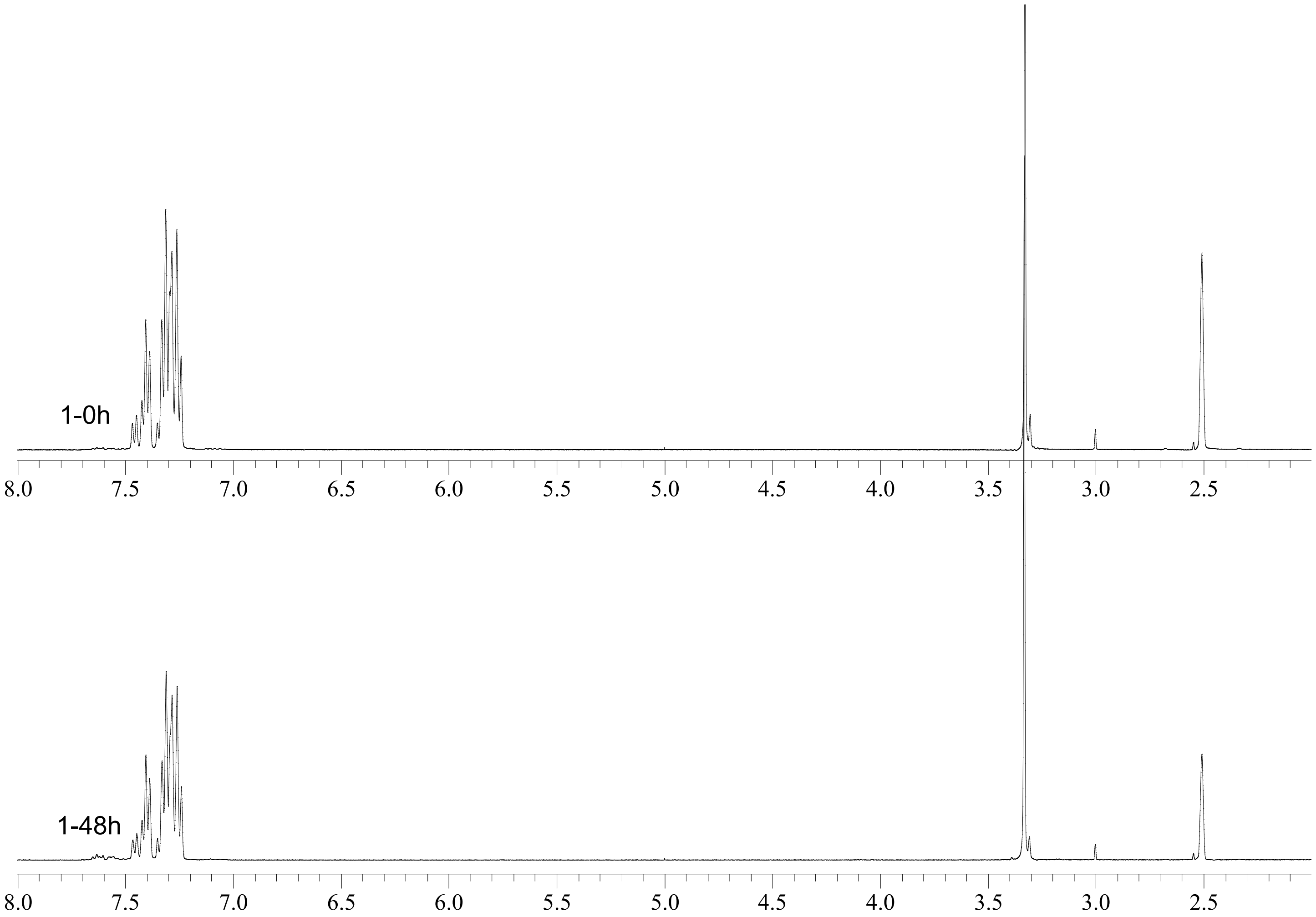

Figure S22. ^{1}H-NMR spectra of **1** in 0 h and the corresponding ones in 48 h, in dmso-d_6

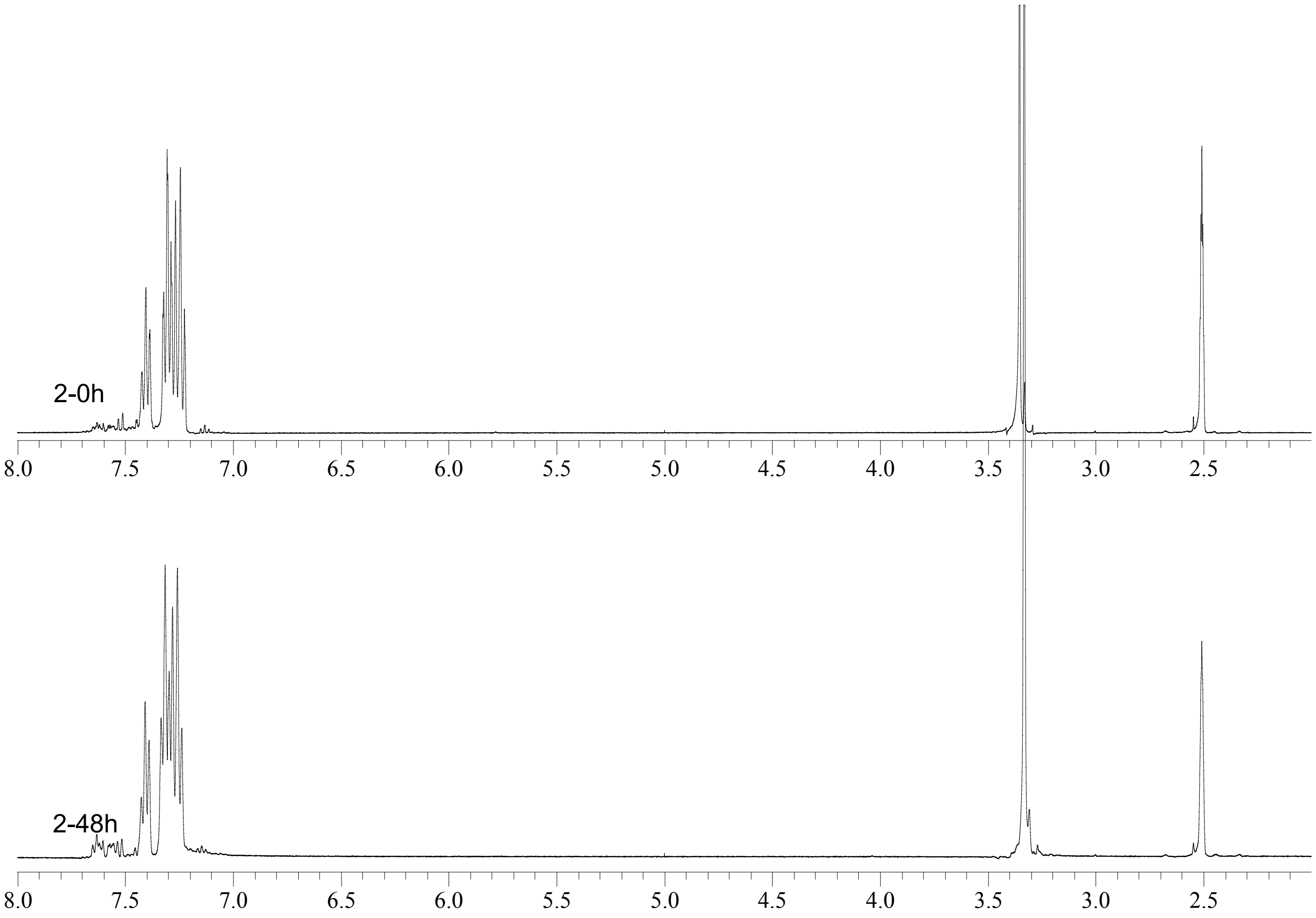

Figure S23. ^{1}H-NMR spectra of **2** in 0 h and the corresponding ones in 48 h, in dmso-d_6

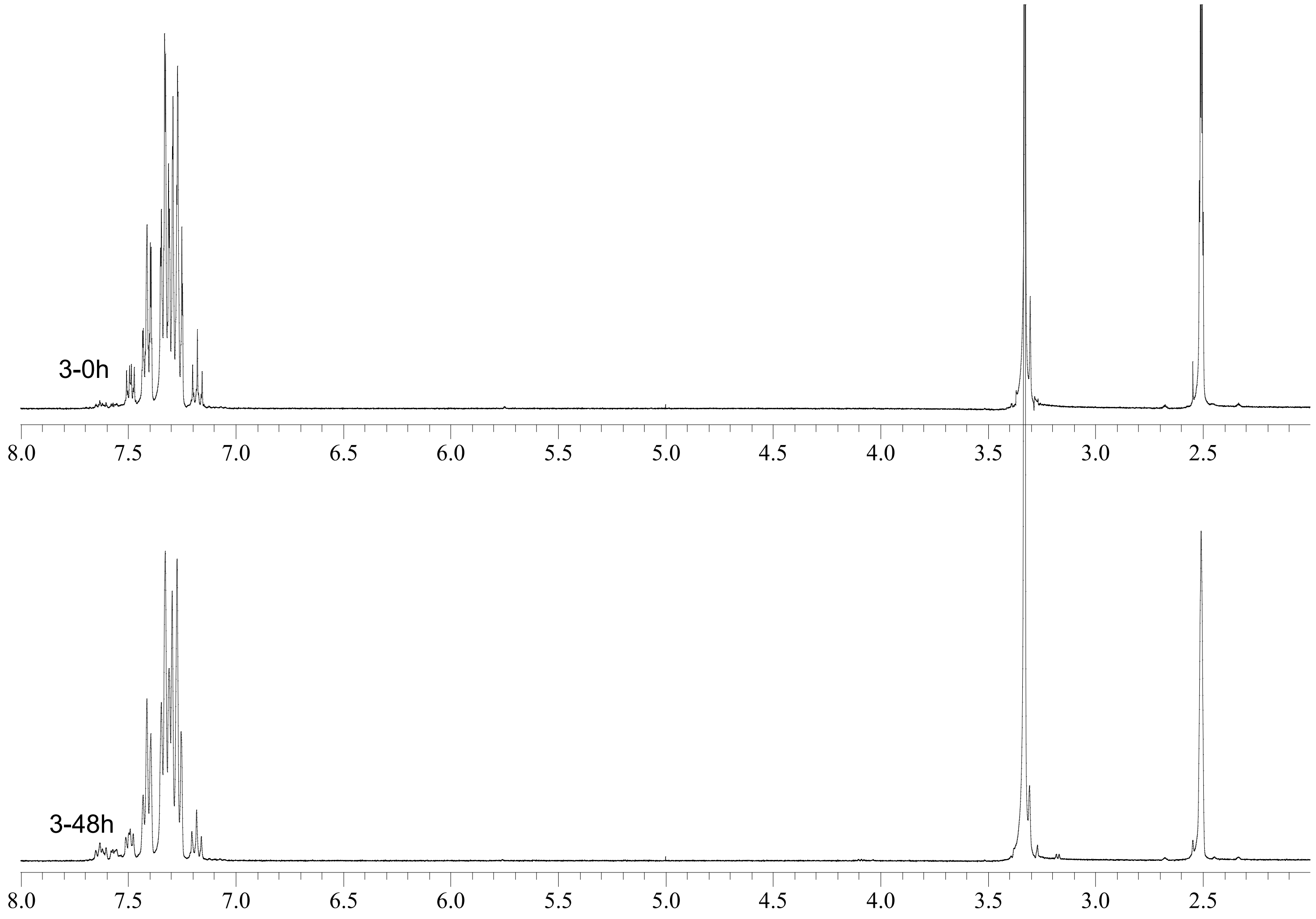

Figure S24. ^{1}H-NMR spectra of **3** in 0 h and the corresponding ones in 48 h, in dmso-d_6

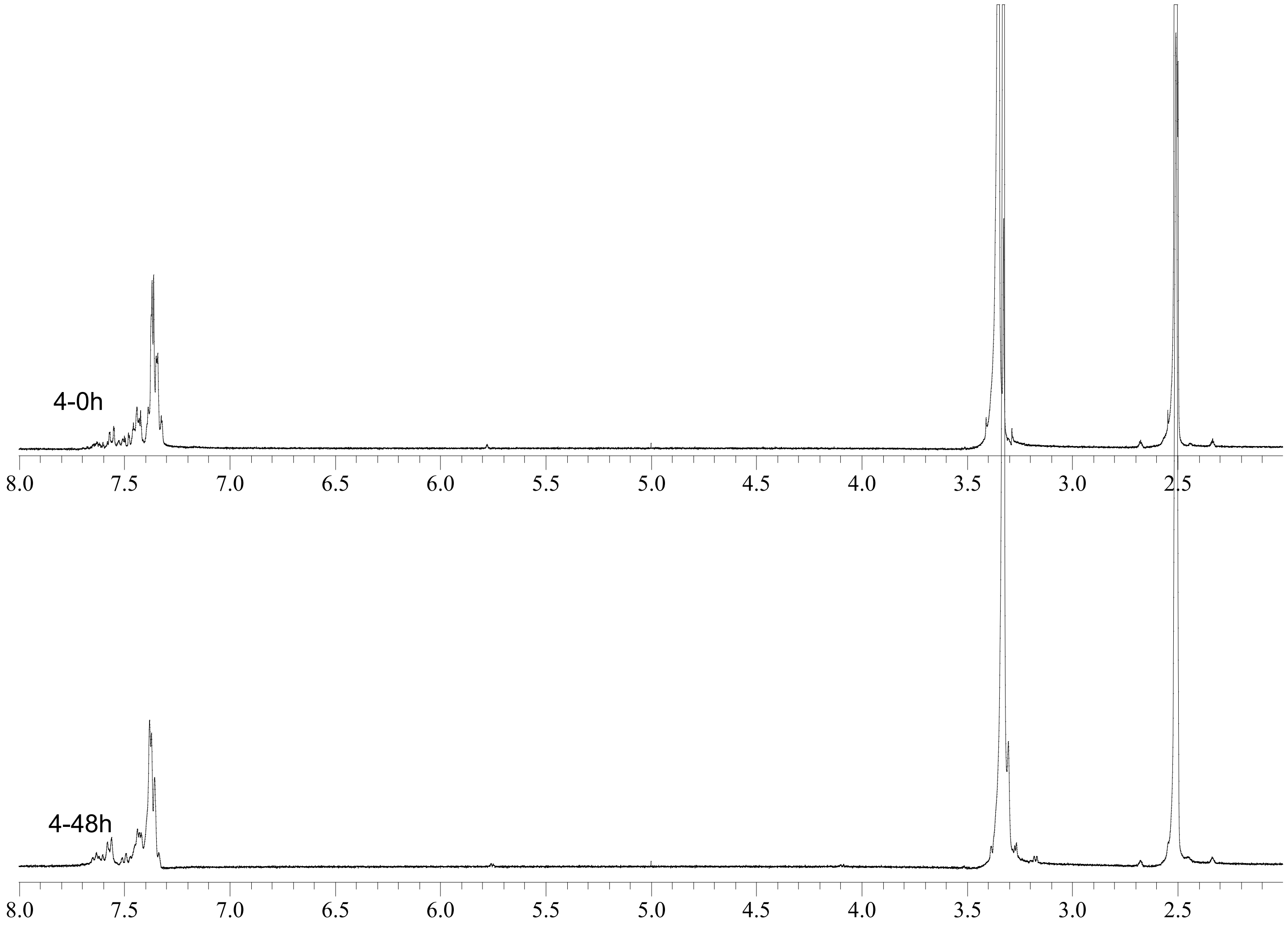

Figure S25. ^{1}H-NMR spectra of **4** in 0 h and the corresponding ones in 48 h, in dmso-d_6

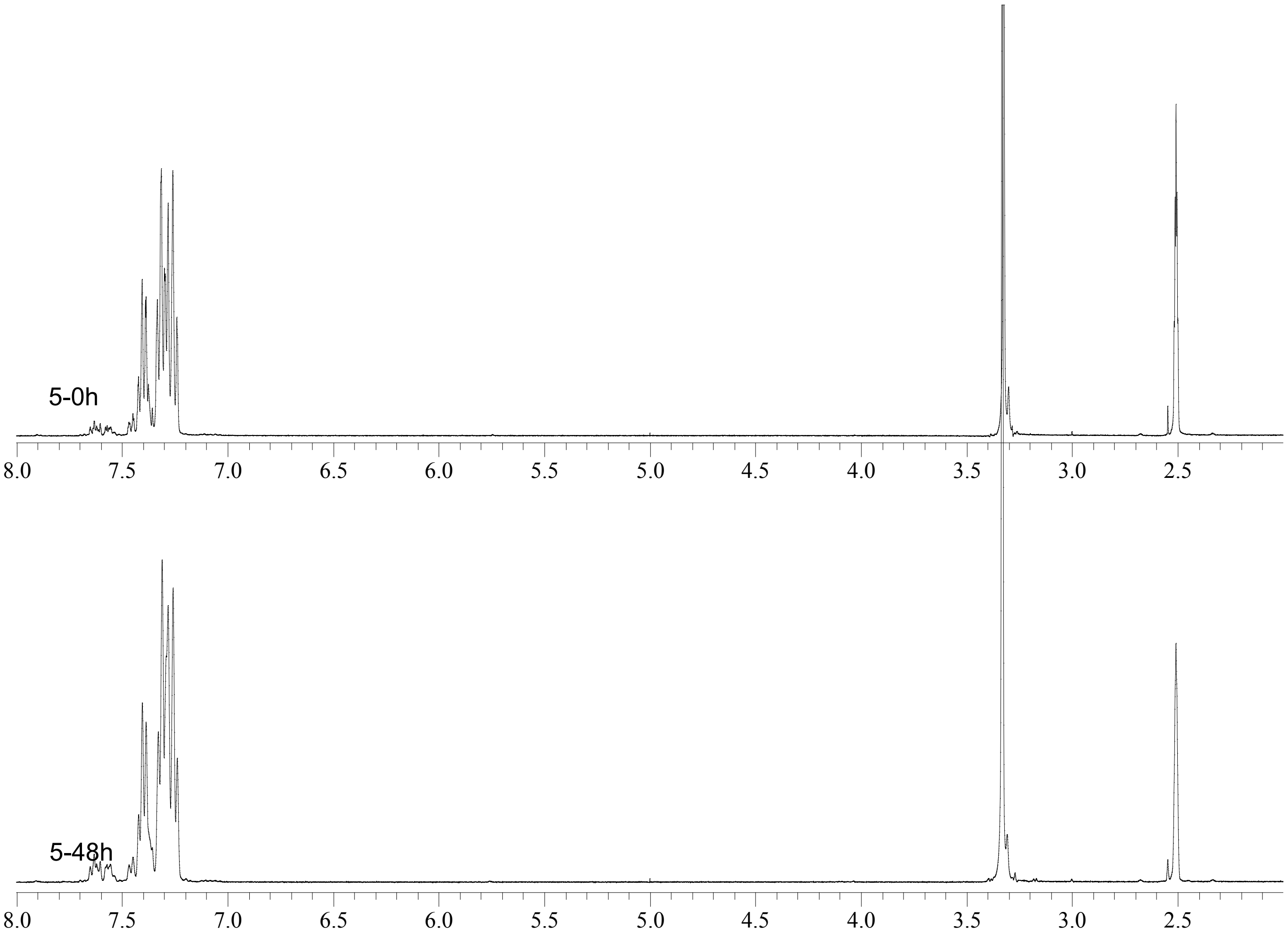

Figure S26. ^{1}H-NMR spectra of **5** in 0 h and the corresponding ones in 48 h, in dmso-d_6

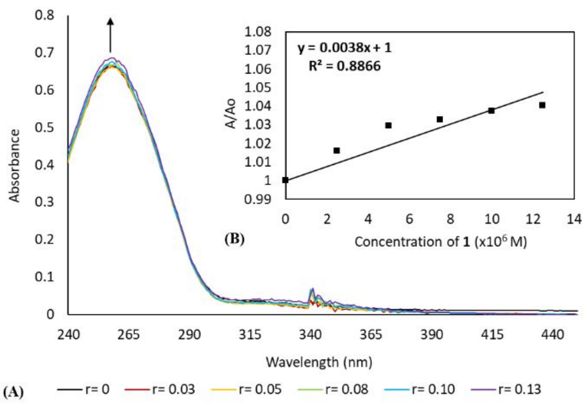

(A)

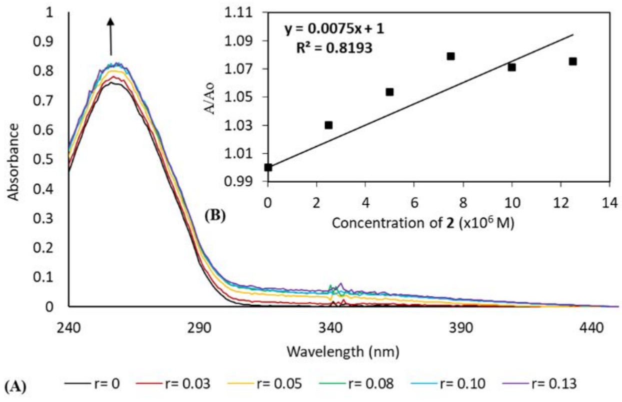

(B)

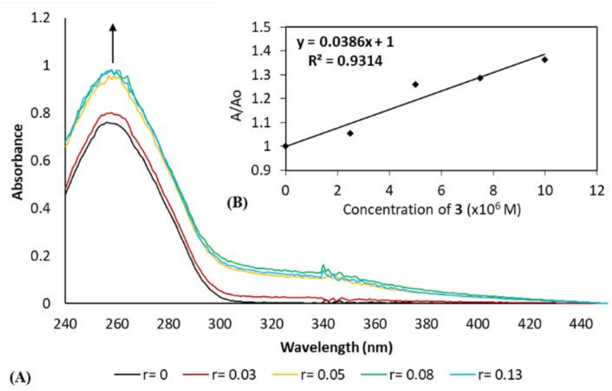

(C)

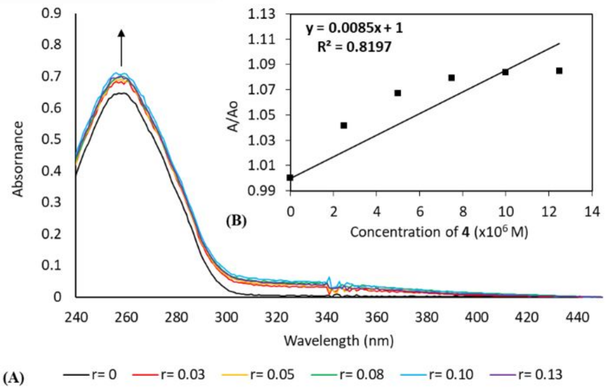

(D)

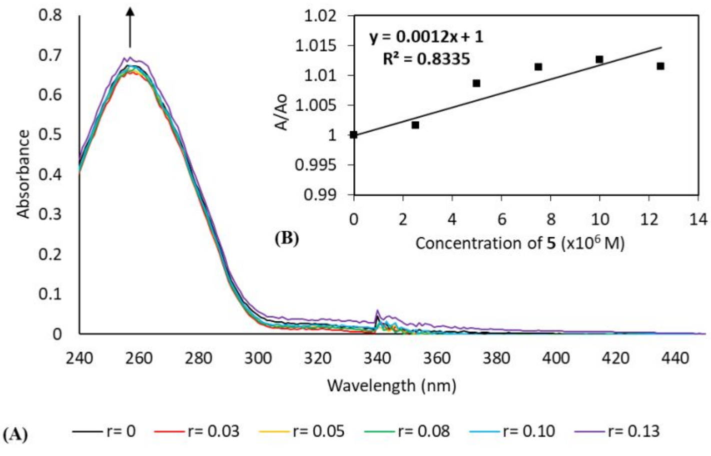

(E)

Figure S27. (A) UV spectra of CT-DNA in buffer solution in the absence and presence of **1** (A), **2** (B), **3** (C), **4** (D) and **5** (E) at r values of 0, 0.03, 0.05, 0.08, 0.10 and 0.13; (r= [complex]/[DNA], [DNA]= 10^{-4} M). The arrow in each panel shows the absorption intensity order with the increase in the r value. (B) plot of A/Ao vs. [complex] at λ_{max}= 258 nm.

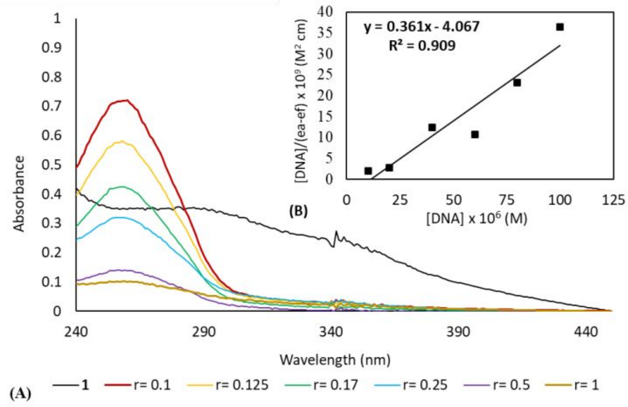

(A)

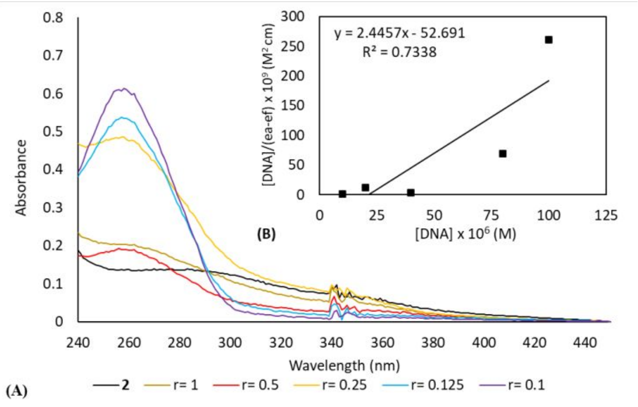

(B)

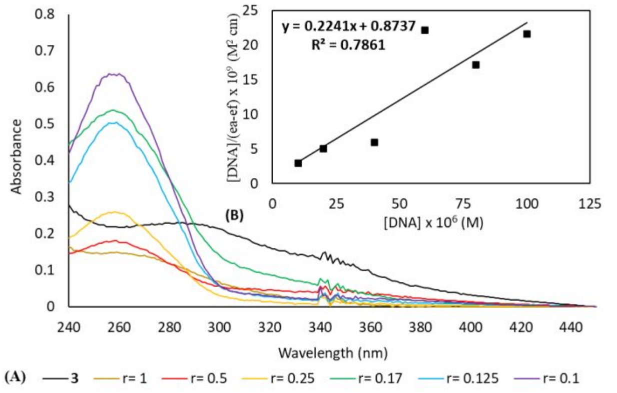

(C)

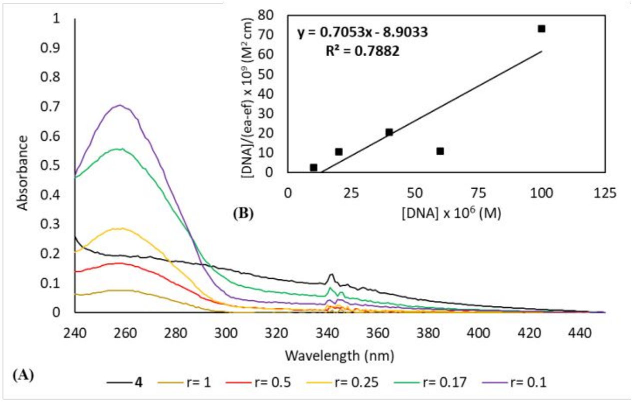

(D)

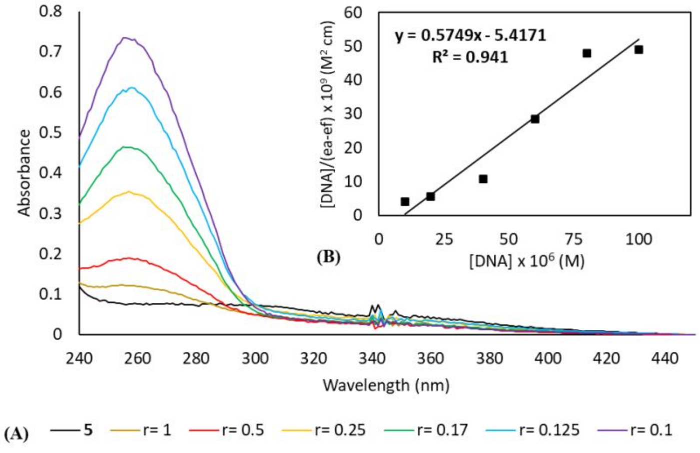

(E)

Figure S28. (A) UV spectra of **1** (A), **2** (B), **3** (C), **4** (D) and **5** (E) in the absence and presence of CT-DNA at r values 1, 0.5, 0.25, 0.17, 0.125 and 0.1 (r= [complex]/[DNA], [Complex]=10 μM, [CT-DNA]=10-100 μM). The spectrum of **1-5** (black solid line) is labelled in each panel. (Insets) Graphical plot of [DNA]/(e_a- e_f) vs. [DNA]

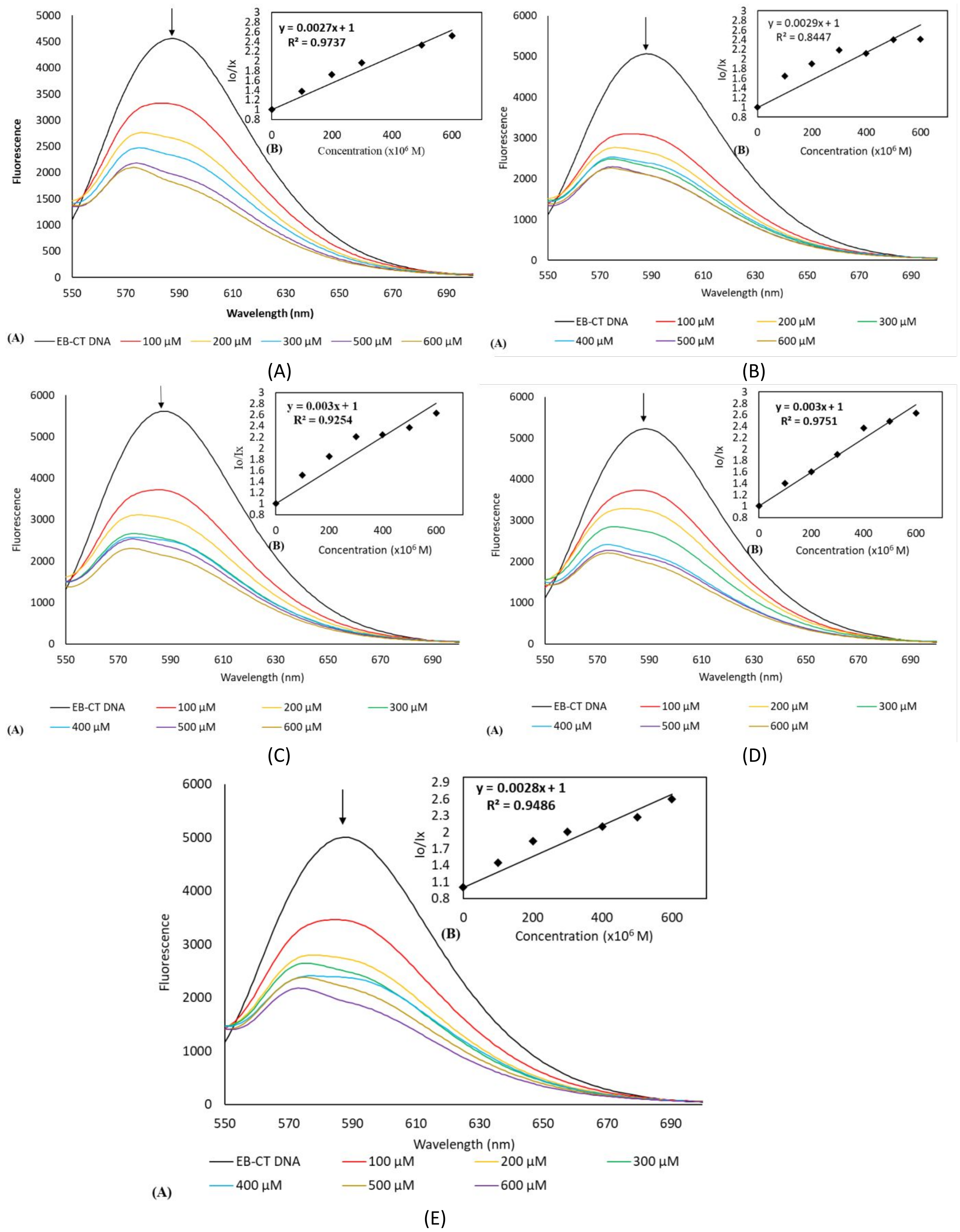

Figure S29. Emission spectrum (A) of CT-DNA-EB complex when undergoes excitation with λ_{exc}= 527 nm, in the presence of **1** (A), **2** (B), **3** (C), **4** (D) and **5** (E), ([EB]= 2.3 µM [DNA]= 26 µM, [complex]= 0-600 µM)). The arrow shows the intensity changing upon increasing complex concentration. Inset (B) shows the plots of emission intensity I_0/I_x vs. [agent].
